# Climate variability, perceptions and political ecology: Factors influencing changes in pesticide use over 30 years by Zimbabwean smallholder cotton producers

**DOI:** 10.1371/journal.pone.0196901

**Published:** 2018-05-10

**Authors:** Cliff Zinyemba, Emma Archer, Hanna-Andrea Rother

**Affiliations:** 1 Environmental Health Division, Centre for Occupational and Environmental Health Research, School of Public Health and Family Medicine, University of Cape Town, Observatory, Cape Town, South Africa; 2 Council for Scientific and Industrial Research, Natural Resources and the Environment, Johannesburg, South Africa; 3 Global Change Institute, University of the Witwatersrand, Johannesburg, South Africa; US Department of Agriculture, UNITED STATES

## Abstract

Pesticides represent a potential public health hazard of note in farming communities. Accumulating evidence indicates that some pesticides used in agriculture act as hormone disrupters, with the potential to result in chronic health effects. Despite such a growing evidence base, pesticides remain the preferred method of pest control in agriculture worldwide. In many parts of Sub-Saharan Africa, usage is on the increase. This qualitative study assessed changes in the usage of pesticides by Zimbabwean smallholder cotton farmers in the past 30 years. Farmers reported an increase in the usage of pesticides, specifically insecticides, since the early 1980s. An increase in pest populations was also reported. The findings suggested a bi-directional causal relationship between the increase in pest population and the increase in pesticide use. Factors which emerged to have collectively impacted on the changes include climate variability, limited agency on the part of farmers, power dynamics involving the government and private cotton companies and farmers’ perceptions and practices. An Integrated Pest Management Policy for Zimbabwe is recommended to facilitate integration of chemical controls with a broad range of other pest control tactics. Continuous farmer education and awareness raising is further recommended, since farmers’ perceptions can influence their practices.

## Introduction

Pesticides continue to serve as the method of choice for pest control in agriculture throughout the world, despite evidence that their usage does not always result in decreased crop losses [1,2]. In many parts of Sub-Saharan Africa, overdependence on pesticides by smallholder farmers appears to be characterised by unsustainable pest control practices, thereby disrupting natural pest control [3]. Furthermore, overdependence on pesticides provides cause for concern regarding human health risks from indirect and direct exposures. For example, pesticides may enter the environment from air, soil and water contamination [4,5], with a likelihood of indirect human exposure [6]. People not directly involved in the handling of pesticides have been observed to become exposed through pesticide drift [7], contaminated water [4,8], as well as consuming food with pesticide residues [9,10]. Pesticide applicators and field workers face the highest risks of direct pesticide exposures through activities such as mixing [11], spraying [12,13] and washing contaminated spraying equipment [14]. Agricultural pesticides thus represent a potential public health hazard of note, particularly for smallholder farmers and their families.

Increasing evidence shows that certain agricultural pesticides have the potential to cause higher health risk to humans than previously assumed [15]. For instance, certain pesticides have, in recent years, proven to be hormone disruptors [16–18], while several are suspected to be [19]. The implication is that these pesticides pose a high risk of interfering with the ability of cells to communicate hormonally [20], by either mimicking or blocking hormones, thereby disrupting the body’s normal functions [21]. For exposed farmers and their families, this may result in a variety of adverse growth, reproductive [22], developmental [23], neurological [24], metabolic and immune effects [21], as well as certain hormone-linked cancers[25]. Smallholder farmers in Low and Middle-Income Countries (LMIC) are at a particularly high risk of negative health effects, since they experience higher rates of pesticide exposures [12]. Any increase in pesticide use, especially those pesticides which contain hormonally-active ingredients, can therefore, potentially increase smallholder farmers’ and their families’ hormone-related health risks.

Despite growing evidence regarding their adverse health impacts, pesticides are still considered to be necessary inputs to certain farming activities, such as cotton production. In cotton, a range of pests may pose a serious constraint to crop production, particularly for smallholder farmers [26]. In Zimbabwe, for example, where cotton is the most pesticide-intensive crop grown by smallholder farmers, up to ten different types of cotton pests are encountered [27,28]. Farmers rely almost solely on pesticides to control such pests during each growing season. Despite such a diversity of pests encountered, there have, however, been assertions that there is, in general, more dependence on pesticides by Zimbabwean farmers than is really required[29,30]. It is likely, therefore, that apart from pest occurrence, there may be other significant factors also influencing the usage of pesticides.

Studies have shown that in addition to the occurrence of pests, several other political-economic factors significantly impact patterns of pesticide use by smallholder farmers in LMICs [31]. Political ecology, which examines the impact of broad scale political-economic factors on local level pesticide use [31], may provide a useful framework for examining these factors. Research shows that increased pesticide use patterns by smallholder farmers are not simply the result of careless and indiscriminate use of pesticides, but are also affected by other factors beyond farmers’ control [10,32,33]. For instance, a study in Tanzania showed that the pesticide industry influences pesticide use by deploying pesticide vendors to farming communities in order to achieve high sales volumes [34]. Research has also shown that agricultural liberalisation in many parts of Africa during the early 1990’s led to easier access to pesticides through the development of illegal and informal trading of pesticides in countries such as Senegal and Benin [35]. In Zimbabwe, the usage of pesticides by smallholder cotton farmers may have been influenced by both government and the cotton industry [36–38], among other factors. During the mid-1990s, when the cotton sector was liberalised, it was observed that about 80% of insecticides used by rural farmers in Zimbabwe were applied in cotton [27]. A key factor impacting high rates of pesticide use in cotton may be linked to the increasing numbers of young people engaging in cotton production as a livelihood due to limited opportunities of employment in the shrunken formal economy. Furthermore, Zimbabwe’s biotechnology policy, which bars the adoption of genetically modified organisms in agriculture, could be indirectly contributing to high rates of pesticide use in cotton production, which could be avoidable. The policy framework constrains the adoption of a genetically modified cotton crop containing genes from the soil bacterium *bacillus thuringiensis*, Bt cotton, which has been credited for reduced pesticide use intensity in certain cotton producing countries (such as India and China) [39,40].

This article uses a political-ecologic lens to describe and explain factors identified to have collectively impacted on changes in pesticide uses by Zimbabwean smallholder cotton farmers of Rushinga district over a period of 30 years. A criticism of political ecology is, however, that focus is limited to the influence of broad-scale structural forces, thus potentially ignoring other influences such as farmers’ behaviours [31]. Farmers have been widely reported to change their pesticide use patterns due to their practices and misperceptions about pesticides [1,34,41]. There is, further, growing recognition that climate variability and change may result in more pests and increased usage of pesticides in agriculture [42]. There have been suggestions that changes in the climatic parameters such as rainfall and temperature may encourage pests which could, in turn, influence increased application of pesticides in agriculture [43–46]. Farmers’ pesticide-use decisions are, therefore, as mentioned earlier, impacted by a multitude of factors, both within and outside their control. This article thus presents Zimbabwean farmers’ perceptions, farming practices, as well as short term climate variability identified to may have impacted on changes in pesticide use, in addition to broad political-economic drivers. By understanding these factors, efforts can be made to minimise dependence on pesticides for pest control by Zimbabwean smallholder cotton farmers. A key objective would be to ensure that policies and practices are not promoting overuse, and that there are mechanisms to effectively address and improve farmers’ pest control practices.

## Materials and methods

The findings presented in this paper form part of a larger study examining whether climate change is a key factor perpetuating health risks associated with human exposures to hormone disrupting agricultural pesticides. As a result of the climate change component in the larger study, 30 years [47] of consistent cotton farming were used as the benchmark for participant inclusion in the study. This study conforms to the consolidated criteria for reporting qualitative research (COREQ-32), comprising a formal 32-item checklist for explicit and comprehensive reporting of qualitative studies [48]. As a way of improving transparency, rigor and credibility, COREQ was used to guide developing aspects of reflexivity, study design, data analysis and the reporting of findings (S1 Table).

### Research design and study area

A qualitative case- study was conducted in the rural cotton farming district of Rushinga, located in North-Eastern Zimbabwe. This research design was used since no pesticide use records were available to allow for longitudinal quantitative analyses. In the absence of these records, assessments based on observations and perceptions by cotton farmers were used in this study.

The population of Rushinga district, based on the 2012 national population census, is 74, 040 people (48% males and 52% females) and 17, 125 households, [49]. Rushinga district lies in Zimbabwe’s natural agro-ecological region IV, which is characteristically hot and dry [50,51]. Approximately 90 percent of households are involved in the production of cotton, which is affectionately known as *white gold* [38]. This district was selected as cotton, a pesticide intensive crop, has formed the main source of income since its introduction in the district during the 1980s.

### Sample selection

Snowball sampling was used for participant recruitment. Those who were recruited had been consistently involved in cotton farming during the past 30 years. As a starting point, names of seven potential participants were proposed by the Rushinga District Agricultural Research and Extension Services Office. Of these, three who met the inclusion criteria were recruited in the study. The other four were no longer involved in cotton production, and were thus ineligible to participate. All initial seven farmers provided 17 names of other potential participants. Throughout the data collection exercise, all farmers contacted were asked to propose names of other potential participants, with a final total of 121 names being provided. Of these, 68 fulfilled the inclusion criteria, with 53 recruited to take part in the study until data saturation [52] was reached. Having reached saturation, it was not necessary to interview the remaining 15 farmers. Interviews were conducted with self-identified heads of households. Participation in the study was entirely voluntary. All participants signed informed consent forms written and read out in their vernacular Shona language prior to the interviews.

Four key informants were recruited to take part in this study. One, a long-serving employee with a cotton company, was interviewed as a cotton industry key informant. This key informant had an educational background in agriculture and had worked for the cotton industry in Rushinga district since 1997, responsible for marketing, pesticide distribution and extension work. Three Rushinga District Agricultural Extension Officers were interviewed as agricultural key informants. For recruiting, the District Agricultural Research and Extension Services Office were initially requested to provide names of officers who had served for up to 30 years in the district. None could be identified. Names of those who had served for up to 20 years were thus requested and five names were suggested. All of them were approached to take part in the study, and three (one female and two males) agreed. The other two declined for personal reasons. In this paper, we present findings from both in-depth interviews with cotton farmers and key informant interviews focusing on pesticide use factors.

### Data collection

Data collection was conducted in two phases, starting with face to face in-depth semi-structured interviews with cotton farmers between July 2015 and December 2015. As mentioned earlier, these interviews were stopped when saturation was reached [52]. In-between data collection, farmers’ interviews were transcribed during January and February 2016. Thereafter, transcribed texts were provided to participants during the months of March and April 2016 for verification and confirmation. All 53 interviews were conducted at farmers’ homesteads by a three-member research team consisting of CZ and two experienced research assistants (female and male) who spoke the local dialect (Shona Kore-kore). Research assistants were first trained by CZ on the aim of the study and the ethics of the study, including the importance of confidentiality of information gathered, participants’ rights and reading out and explaining the informed consent. Farmers were asked questions concerning their experiences with and observations about changes in pest populations and pesticide-use practices in the past 30 years (S2 Table). All interviews were audio-recorded with participants’ written consent.

Phase two of the study involved face-to-face interviewing of four key informants in April 2016. These were conducted after the initial analysis of farmers’ interviews as a way of explaining, corroborating and triangulating themes in the data, as well as collecting sector insights. Questions for the agricultural key informants were designed based on emergent patterns in the data collected from the farmers. These included their knowledge and perceptions about increases in pest populations and increases in pesticide use by farmers. Key informants were further questioned about their roles as extension workers and the role of their parent ministry, which enabled making inferences about their respective influences in the usage of pesticides. The cotton industry key informant was interviewed relating to the role of the cotton industry, such as what factors they consider to be important when drawing-up contracts with farmers, the role of the cotton industry in managing pests and pesticide resistance, and the nature of training which they give to farmers.

### Data analysis

Three participating farmers’ interviews were excluded from analysis after quality control, due to inconsistencies which could not be resolved. Of the remaining 50, there were 36 male heads of households, nine female heads of households and five couples who chose to be interviewed together. All 50 interviews were transcribed *verbatim* and then translated into English (S1 Text). Upon completion, transcripts and their corresponding audios were analysed by a professional translator for consistency and accuracy. A qualitative data analysis software, NVivo (version 11), was used for transcript management and aiding data analysis, particularly coding. Coding involved a four stage process starting with attribute coding, followed by structural coding, magnitude coding and descriptive coding techniques [53]. Guided by magnitude questions asked during interviews, structural coding was used to code entire transcripts into five broad data categories (structural codes)—namely: pesticide-use, pesticide efficacy, pesticide sufficiency per hectarage, pest populations (Table 1) and participant demographics. Thereafter, attribute coding technique was used to assign codes for participants’ attributes such as age, gender, and years of cotton farming.

**Table 1:** Some interview questions and codes developed from data.

Questions	Structural codes	Magnitude codes	Descriptive Codes
1. Would you please describe how the quantity of pesticides which you use on your farm has changed over the past 30 years?	pesticide use	(+) increase	n/a
		(=) no change	
		(-) decrease	
2. Would you please describe how the population of all pests which you encounter on your farm has changed over the past 30 years?	Pest populations	(+) increase	n/a
		(=) no change	
		(-) decrease	
3. Do the pesticides which you currently use on your farm kill insects when you use them according to instructions?	Pesticide effectiveness	n/a	Effective
			Not Effective
4. Are the quantities of pesticides which you currently receive from contractors to use on your farm enough to control pests which you encounter throughout the season?	Pesticide sufficiency	n/a	Sufficient
			Not sufficient

The remaining four categories were analysed in more detail, using magnitude coding and descriptive coding techniques. Magnitude codes are qualitative, quantitative and/or nominal indicators which are used to indicate intensity or frequency of phenomenon [53]. In this study, magnitude codes indicated changes that happened in the past 30 years in as far as pesticide use and pest populations are concerned. Mathematical symbols were used for magnitude codes; ‘‘+’’ indicating an increase, ‘‘—’’ indicating a decrease, and ‘‘=’’ indicating no change. Descriptive coding technique was then used to code details pertaining to current pesticide efficaciousness and current pesticide sufficiency (Table 1). The rest of the interviews which were not analysed using the aforementioned coding techniques, together with all the key informant interviews, were analysed by thematic analysis [53]. By this process, data were analysed with thematic statements, rather than short codes (S3 Table). This led to the emergence of three themes from the data, namely: (i) climate variability, (ii) political ecology and (iii) farmers ‘perceptions and practices.

### Ethical statement

Ethical approval was granted by the Human Research Ethics Committee of the University of Cape Town’s Faculty of Health Sciences (HREC Ref: 300/2015). In Zimbabwe, further approval was granted by the Ministry of Health and Child Care’s Epidemiology and Disease Control Directorate and the Ministry of Home Affairs through Provincial Minister for Mashonaland Central Province, the Rushinga District Rural Council and the Rushinga District Administrator.

## Results and discussion

Participants’ ages ranged between 54 to 73 years. Cotton farming experience in years ranged between 30 and 43, with 33 years being the average, as most farmers started growing cotton in 1982. All 50 farmers reported consistently using chemical insecticides to manage insect pests for cotton farming. Asked about the methods of pest control which they used upon detection of pests, 76 percent of farmers who responded to the question (or 64 percent of all participating farmers) indicated that they exclusively relied on chemical pesticides (Table 2).

**Table 2:** Methods of pest management.

Pest control method	% farmers (n = 42)
Exclusively pesticides	76
Integrated—pesticides and other methods such as hand-picking worms, spraying aphids with crashed chillies, crashed herbs, or a washing powder mixture.	24
Exclusively other methods (no pesticides)	00

To control weeds, only a few farmers (n = 11) had ever used herbicides in their three decades of cotton farming. All farmers indicated that they controlled weeds physically by using hand hoes, while none relied on herbicides exclusively. Due to relatively low usage of herbicides, farmers generally used the word ‘pesticide’ to refer to insecticides, except where a distinction was made between herbicides and insecticides. Likewise, in this study, the same terminology is adopted.

### Increase in pesticide use

**Fig 1:**
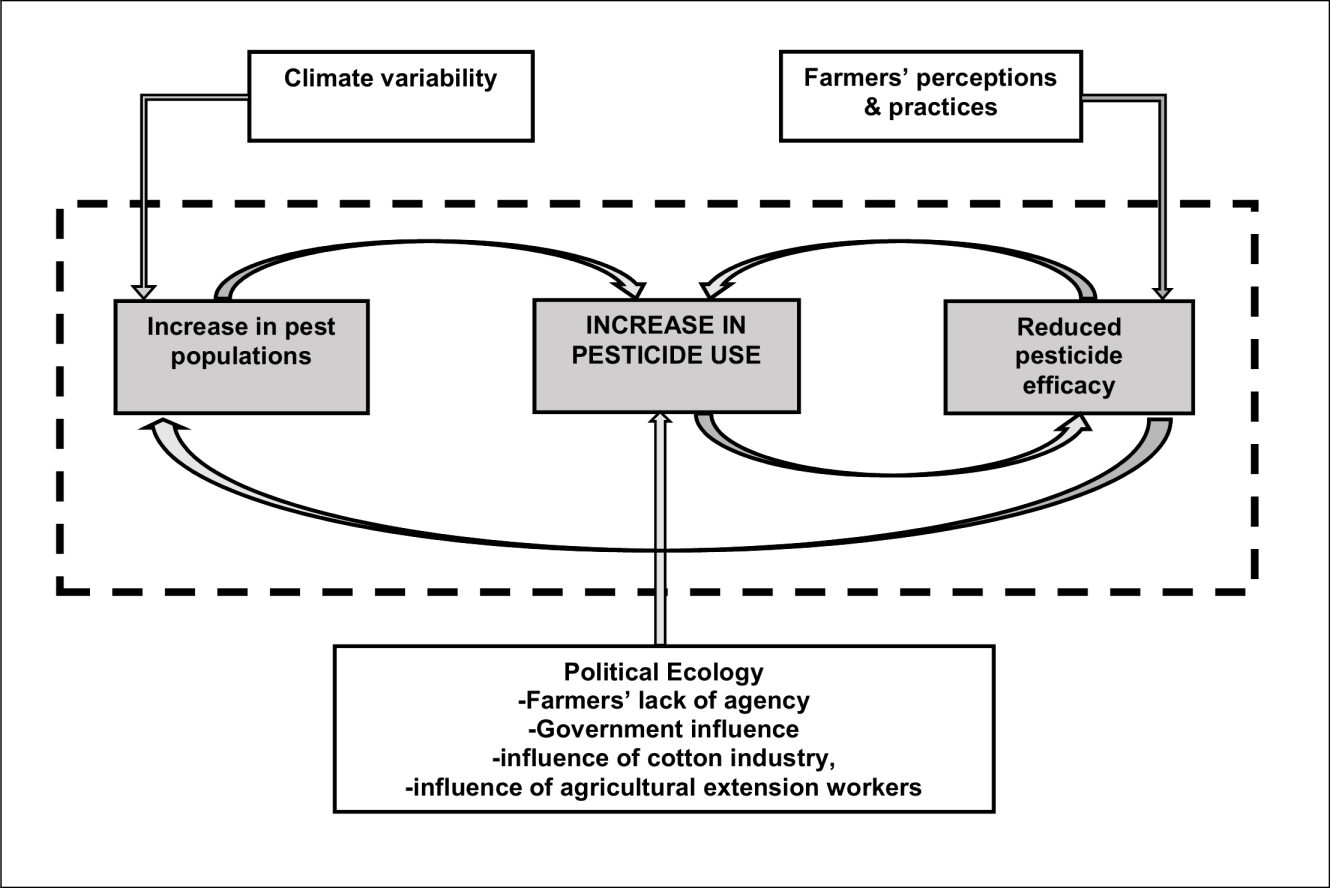
Changes in pesticide use and pest populations.

Responding to the first substantive magnitude question, most farmers (60%) reported an increase in pesticide use from the time each farmer started growing cotton up to the 2014/2015 season. Furthermore, 84 percent of the farmers perceived an increase in pest populations on their farms during the same period (Fig 1).

Reported increases in pesticide use ranged from double, on average, to several times in outlier cases. The following farmer’s comment illustrates:

*‘‘The pesticides that we used in the past were very strong*. *We used to get 200ml*, *and we would use the same small bottle till we harvested*. *But*, *nowadays we have the types of Lambda which we get in 500mls*. *Just that one 500ml bottle is not enough*. *You need four or five of such bottles for you to be able to harvest*, *which means the pesticides that we are getting nowadays are very weak*, *they do not have power”* (Respondent: CZ 04).

It is of interest to note that 22 percent reported a decrease in pesticide use, while 18 percent indicated that there had been no change in pattern and quantity of applications. However, nearly all farmers had the perception that the total amount of pesticides they were using were not commensurate with changes in pest populations on their farms. Ninety four percent of farmers believed that the amounts of pesticides they were using on their farms were not sufficient for their pest control needs.

### Factors influencing pesticide use increases

**Fig 2:**
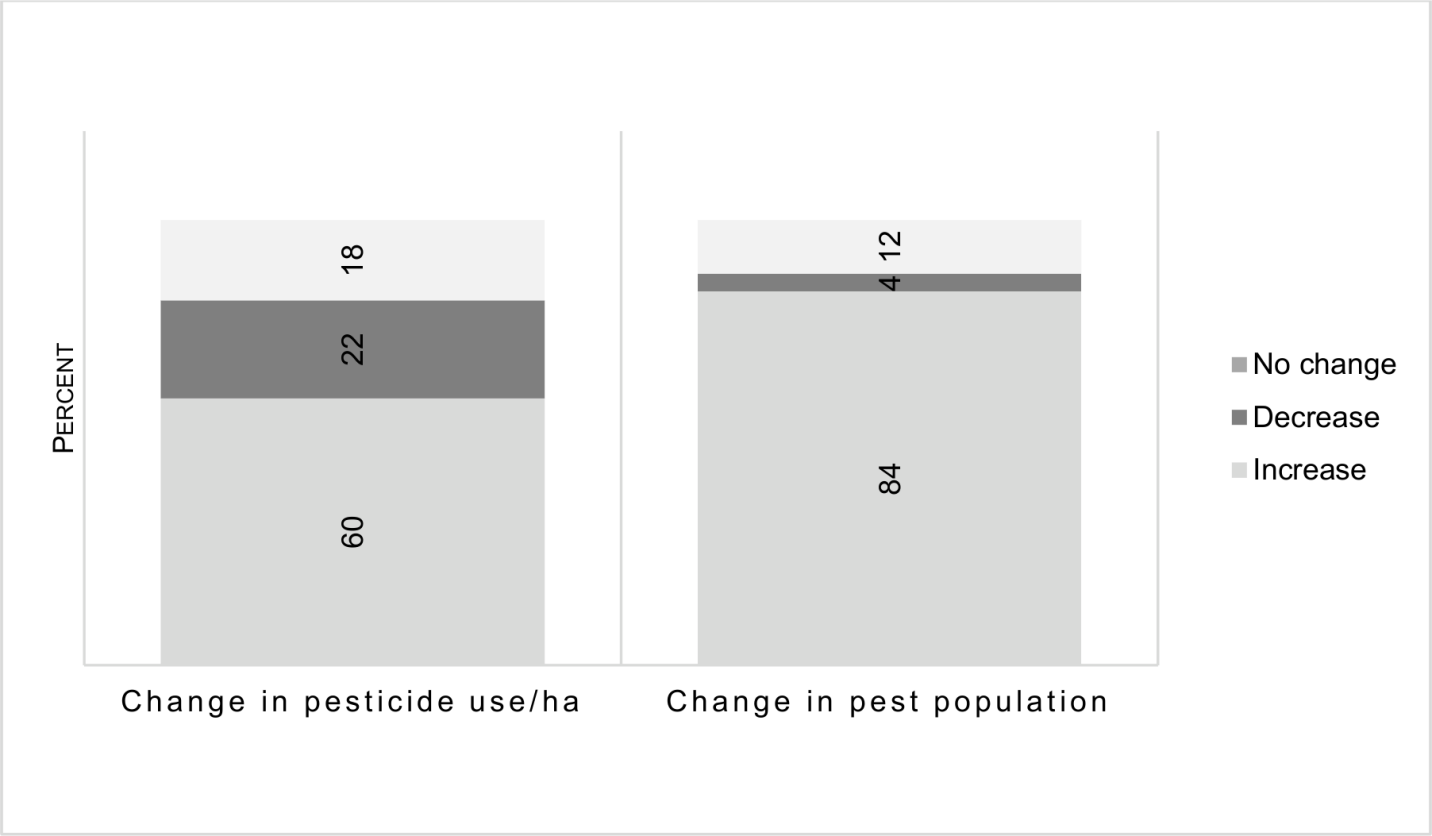
Influence of climate variability, perceptions and political ecology on Zimbabwe farmers’ pesticide use.

Increases in the usage of pesticides in Rushinga appear to be impacted by a combination of, amongst others, three key influencing factors explored during thematic analysis (shown outside the dash triangle in Fig 2). The first of these factors was developed through a political ecology lens, focussing on power dynamics between farmers and agricultural institutions which impact on pesticide-use decision making. Political ecology, while directly impacting on the usage of pesticides, further has an indirect impact on pest populations and pesticide efficacy. The second key influencing factor pertains to farmers’ perceptions, including their practices, which have a direct impact on the efficacy of pesticides, while indirectly impacting on pest populations and, thus, the usage of pesticides. The third key influencing factor, over which farmers (and institutions) have no direct control, but appear to be responding to, is climate variability. Climatic variation in Rushinga District seems to have a direct impact on pest populations, while indirectly impacting on pesticide use and pesticide efficacy. These three influencing factors are discussed in the following sections in detail.

### Political-ecological impact

The findings in this study concur with findings from other parts of the world which attest that there are political-economic forces which may significantly influence patterns of pesticide use in agriculture [31]. In Rushinga district, usage of pesticides by smallholder cotton farmers has been, to an extent, influenced by institutions and actors such as the government, cotton companies and agricultural extension workers. All participating farmers indicated that they had always grown cotton under contract farming arrangements; which meant they received inputs (e.g., pesticides, chemical fertilisers and seeds) from the government and the cotton companies on credit. This dependence on credits effectively limits their agency–that is, they have never been fully in charge of their pesticide use decision making process. Instead, decisions made by the government, cotton companies and agricultural extension officers seem to have significantly influenced farmers’ pesticide use. The roles of these institutions and actors are expounded on below.

Key informants indicated that cotton was officially introduced in Rushinga district in 1982 by the government. For 12 years thereafter, the government held a monopoly over the cotton sector, being the sole supplier of inputs such as seeds, fertilisers and pesticides. During this period, the government made limited effort to provide farmers with the know-how to farm with an integrated pest management approach that does not rely solely on chemical control (e.g. cultural and biological pest control methods).

The sector was liberalised in 1994, paving the way for private companies to become involved in cotton production and marketing [38]. However, two decades into liberalisation, the legacy of exclusive use of pesticides for pest control persisted. According to the cotton industry key informant, cotton companies have largely been unsuccessful in their quest to break the culture of exclusive pesticide use through their pest management training programmes. It appears, thus, in Zimbabwe, that government support of the usage of pesticides in cotton farming is precedent. This is, however, not peculiar to the Zimbabwean situation. It has been observed that a number of governments in Africa significantly influence pesticide use patterns through extension programs which encourage the usage of chemical pesticides [34,54].

Despite liberalisation, the government of Zimbabwe remains responsible for key research and regulatory issues which impact on the usage of pesticides through the Ministry of Agriculture’s Cotton Research Institute (CRI). The CRI is mandated by the government to, among other functions, develop pesticide rotation and spraying calendars for different regions of the country as a way of managing pesticide resistance by limiting the period during which broad-spectrum pesticides may be used [28,55]. However, spraying calendars tend to encourage increased use of pesticides, as they base spraying on calendar dates rather than scouting practices and observations of pest incidences.

The contribution of the cotton industry in the increasing usage of pesticides in Zimbabwean smallholder cotton farming has been through its unequal power relations with farmers. These unequal relations are summarised in Table 3.

**Table 3:** Contribution of cotton industry to pesticide use increases.

Theme	Explanation
Disregard of contracts	Pesticides are often distributed late, when farmers would have already bought contingency supplies, resulting in pesticide accumulation.
Power and control	The industry determines the pesticide brands and the quantities of pesticides to be distributed to farmers. Farmers have little or no agency.
Rigid contracts	Contracts empower industry to attach farmers’ property, resulting in fear of losing property providing motivation for excessive pesticide use.
Industry standards	Lowly-grade cotton due to pest attack is poorly remunerated both on the local and global markets.
Pesticide market	Presence of a thriving and unregulated local pesticide market in which the cotton companies are players of note.

The local cotton industry supplies inputs, and then guarantees purchase of farmers’ produce, paying them the difference between the market price at the time of selling and the cost of inputs [37]. When agreements are made under contract farming, farmers sign for three distinct classes of pesticides. However, it is the companies which determine the specific brands and quantities per hectare of pesticides to be issued to farmers, as well as the timing of delivering these pesticides to farmers. This effectively limits farmer agency, with limited ability to make important decisions governing their pesticide use patterns.

The supply of pesticides constitutes a critical component of contract farming in the Zimbabwean cotton industry. Companies have, however, often been implicated for failing to honour their contractual obligations [56]. For instance, the pesticide distribution system was described by both farmers and key informants as inefficient. Farmers, on occasion, receive pesticides late when they would have already bought some on a cash basis—ironically from the cotton companies themselves, if not from the agricultural input stockists. One farmer remarked:

*‘‘The companies come and make us sign contacts and just give us seed*. *They promise to supply the chemicals and fertilisers later*. *Hoping the companies would honour their contractual obligations to supply the said inputs before the appearance of pests*. *However*, *they tell us later that they cannot supply us with anything else”* (Respondent: CZ 17).

When they finally receive their contractual pesticides, some farmers make use of them, despite no apparent need as a way of simply getting rid of them before the end of the season due to safety concerns [57]. A few farmers, however, noted that when pesticides are disbursed late, they store them for use the following season, even when pesticides, such as acaricides, would be rotated. Zimbabwe cotton farming regions follow a three-year acaricide rotation scheme aimed at managing resistance [28]. Failure to rotate may be seen to promote resistance due to lengthy exposure to increasing amounts of the same pesticide.

The terms of contracts signed between cotton companies and farmers appear to contribute further to the need for farmers to increase their usage of pesticides. The cotton industry key informant noted that when farmers sign for input credits, they sign off their livestock, or any other movable properties they possess, as surety. Farmers are fully informed that when they fail to adequately control pests they risk having their property attached by the cotton companies. The increase in the usage of pesticides is, therefore, partly influenced by a secondary motivation to protect assets against attachment.

It also appears that stringent global market demands for high quality cotton play a crucial role in making pesticides critically important in cotton farming. Low quality cotton, compromised by pests, is poorly remunerated, and it often fails to attract break-even purchase prices for farmers. Market demands for cotton of high quality which can compete on the export market may be pressuring farmers to use higher quantities of pesticides in controlling pests. This pressure put on farmers has resulted in the industry seeing an opportunity to develop a pesticide market targeting smallholder farmers. According to both farmers and key informants, before liberalisation, no pesticide dealers were present in Rushinga district, and the local agricultural input stockists did not sell any pesticides. Thus, without anywhere to purchase additional pesticides, pesticide quantities used did not exceed government controlled limits. After liberalisation, however, both agricultural input stockists and cotton companies commenced selling pesticides to any farmers who needed additional supplies. Observations by the research team revealed that several agricultural input stockists in the district stocked pesticides throughout the year. Liberalisation thus opened a cash-basis pesticide market which may be contributing to higher usage of pesticides by affording farmers.

All farmers indicated that they had been educated about cotton farming, identification of pests, as well as the safe handling of pesticides by the agricultural extension workers whom they regarded to be the primary source of expert information. The extension workers, however, appeared to advise farmers in ways that reinforce a paradigm of pesticide reliance. All three agricultural extension workers noted that their core responsibility is teaching farmers about good farming practices, including proper usage of pesticides. They believed that their influence on farmers’ farming practices, including the usage of pesticides, was significant. They remarked that they encourage farmers to use both insecticides and herbicides. They also argued that the advantages for farmers of using herbicides, for example, include health benefits as illustrated by the following key informant’s comment:

*‘‘The herbicides are very good*. *It is unfortunate that these companies do not give any to our farmers here*. *We would want our farmers to have access to cheap supplies because herbicides will make their workload lighter*. *You have seen them*. *Don’t they look old*? *They are still very young; they look old because weeding manually with the hoes makes people age faster*. *When they spend several hours*, *each week*, *weeding it’s not good for their health”* (Key Informant: CZKI 01).

Even though this study observed that farmers generally regarded the extension workers as experts, most farmers had not heeded advice to use herbicides. Instead, farmers always preferred to weed manually, as this was a cost-effective alternative to herbicides use.

### Farmers’ perceptions and practices

Previous studies have observed limitations of political ecology theory, and shown that its focus on political-economic influences alone ignores how farmers’ perceptions and practices impact on the usage of pesticides [31]. Taking cognisance of this, the current study considered how farmers’ perceptions and practices influence increased usage of pesticides. In Rushinga district, farmers’ attitudes and perceptions about the efficaciousness of pesticides appear to have also contributed to increases in the usage of pesticides.

Certain of the farmers interviewed revealed misconceptions about benefits that can be derived from pesticides, which might have also contributed to increased pesticide use. For instance, certain farmers understood the purpose of pesticides to be more than simply that of controlling pests. Respondent CZ 12, for example, believed that carbaryl 85, a carbamate pesticide whose mode of action is interference with insect nervous systems, has some fertiliser properties.

*‘‘… we use Carbaryl 85*. *This pesticide provides feeding to the cotton*. *The leaves remain green*, *and the crop will be healthy*. *If the leaves are not healthy*, *then we do not have cotton*. *A tree without good leaves is not a tree*. *So*, *we use Carbaryl*, *it gives feeding”* (Respondent: CZ 12).

Such misconceptions by certain smallholder farmers of Rushinga district are consistent with findings of other studies elsewhere, which have shown that perceived benefits from pesticides have an impact on farmers’ pesticide use patterns [1,58,59].

When farmers’ perceptions about pesticide efficacy were sought, only eight percent believed that the pesticides currently supplied by industry were effective in controlling their cotton pests while the majority (92%) believed otherwise.

Pesticides were described as having become *‘‘weak”*, or having *‘‘lost their power”*. Certain farmers believed that the pests, rather than the pesticides had changed. They described pests as having become *‘‘addicted”* or *‘‘used”* to the pesticides. Their perceptions were suggestive of one thing—pesticides were no longer considered to be efficacious in controlling pests. In particular, some farmers interviewed had a negative view of broad-spectrum pyrethroid pesticides, such as lambda-cyhalothrin, which they perceived as no longer efficacious against pests. Bollworms were particularly reported to be surviving pyrethroid applications. Some of the quotations that capture farmers’ perceptions about pests and pesticides possibly contributing to pesticide use are presented in Table 4.

**Table 4:** Farmers’ general perceptions about pests and pesticides.

**Some Perceptions about pests**
• *Maybe it’s because the worms are now used to the pesticides*
• *either the pesticides have lost power or they still have power*, *but the pests themselves have developed resistance*.
• *worms go deep in the ground to the root of the plant as a way of dodging the pesticides*.
• *What happens is*, *if we just spray anyhow*, *those pests will get addicted to the pesticides and in the long run they will not die*.
• *The increase in pest populations is because the pesticides are not able to kill*.
**Some Perceptions about pesticides**
• *I think they (pesticide manufacturers) have reduced the strength of the pesticides*
• *pesticides are no longer powerful*, *as powerful as they used to be*
• *when they expire they never tell us that these pesticides have expired … we are buying pesticides that are expired*.
• *In the past*, *the pesticides were much stronger*.
• *They are intentionally making the pesticides ineffective so that farmers would return to buy more*.
• *I also think it’s because these cotton companies … also give us expired pesticides as they cannot afford to lose money by throwing them away*.
• *It's the same pesticides*, *but these days they use counterfeit pesticides*.

Farmers’ observations about pests, and their perceptions about why pesticides were no longer efficacious, were leading them to engage in practices possibly contributing to increases in pest populations (Table 5). For instance, some of the farmers who perceived that their pesticides were no longer efficacious reported manipulating their pesticide preparation formulae by mixing more pesticides together with the same volume of water than is recommended. For example, one farmer remarked:

*‘‘I double the concentration of the pesticides*. *If the label directions say that I should put only 30 mls in my 15-litre knapsack*, *i simply double and make it 60mls”* (Respondent: CZ 06).

**Table 5:** Selected practices by farmers contributing to increases in pesticide use.

Farmers’ practices
• Mixing several pesticides in the same knapsack to make concoctions
• Calendar-based spraying rather than scouting-based spraying.
• Maintaining ratoon cotton crops.
• Using wrong pesticide to water ratios when mixing pesticides.
• Preventive spraying.

Other farmers reported mixing several types of pesticides in the knapsacks to make cocktails before spraying. Such farmers believed that cocktails would be more efficacious in controlling bollworms which they reported to be unresponsive to pyrethroid treatments. Some farmers, such as Respondent CZ 16, engaged in preventive spraying, under the impression that bollworms would be exposed to the pesticides before they got the chance to infest their cotton. However, such prophylactic treatments and usage of cocktails could risks genetically predisposing pests to be resistant to particular pesticide formulations [2,60]. Resistance is failure of a pesticide to achieve an expected level of control on a pest population, which is caused by repeated exposure of the pest population to a particular pesticide [61,62]. There is, therefore, need for further research in future to ascertain if there is pesticide resistance due to farmers’ practices in the study area.

*‘‘If you spray your cotton with lambda before the worms are present*, *they will die*. *But*, *if you delay and get in to spray after the red bollworm has entered the bolls*, *controlling it is very difficult”* (Respondent: CZ 16).

More than three quarters of farmers (82%) indicated that they used self-developed spraying calendars, which required them to spray at regular intervals, which are essentially weekly spraying time tables. During the 1980s and the 1990s, Rushinga farmers sprayed on average fortnightly, after having done scouting. Previous studies have concluded that because calendar based spraying is not based on field observations, it is not the best approach to pest control [26,63]. For example, in several Francophone African countries, where spraying was entirely based on a calendar schedule until the late 1980s, researchers have inferred that this approach may have contributed to the development of pesticide resistance [63–65].

In Rushinga, the use of calendar spraying rather than scouting and predetermined action levels [61] may have resulted in farmers using increasing amounts of pesticides, particularly pyrethroids, resulting in the unintentional elimination of beneficial insects. As mentioned previously, scouting is important as it aids farmers’ decision-making processes, avoiding application of pesticides when pest populations are too low, thereby allowing the build-up of natural enemies. It has been suggested that elimination of beneficial insects by the overuse of pyrethroids may result in increasing pest population, due to an indirect effect of pesticide-induced resurgence [28,64].

The usage of pesticides may have well increased in Rushinga district due to intentional, but ill-informed, elimination of beneficial insects, which would otherwise play a key role of controlling insect pests. Some farmers reported killing predators, because predator activities were deemed to have a negative effect on the quality of cotton. For example, ladybird beetles were considered undesirable as illustrated in the following comment:

*‘‘There have been a lot of the red ones the ladybird beetles in the recent years*. *They appear at the end*, *when the cotton bolls start opening*. *They discolour the cotton and make it become brown in colour*. *But we spray them with Carbaryl 85 and manage to control them*. *These ones die quickly”* (Respondent: TM 22).

Another group of farmers targeted beneficial insects, such as syrphids [66] which predate on aphids [67], due to their inability to recognise them. For instance, some farmers mistakenly identified predator insects as cotton pests. One farmer remarked that:

*‘‘There are also good insects that are our helpers that we see eating some of the (bad) insects*. *For example*, *there are syrphids which feed on eggs and smaller moths*. *But*, *the problem with the syrphids is that their excreta resemble that of spine* (spiny bollworm). *Therefore*, *we as farmers*, *may not always recognise the difference and start spraying thinking that there is spine”* (Respondent: CZ 11).

Due to these misconceptions, certain farmers use significant amounts of pesticides to eradicate harmless, and, in some cases, beneficial insects. This is indicative of the need for training in cotton pest identification and integrated pest management.

### Climate variability

Climate change in Zimbabwe is characterised by erratic rainfall patterns, warmer temperatures and an increase in the intensity and frequency of mid-season dry spells [68,69]. Local climate data for the region of study were not available to the authors since there was no weather station. Nevertheless, interviewed farmers indicated that inter-annual variability in rainfall was often associated with defined pest activity. For instance, those seasons characterised by low rainfall and long dry spells during the rainy season were often observed to be associated with high pest incidences. Aphids, in particular, were reported to characteristically increase in population following prolonged periods of dry weather during the rainy season. One respondent noted that:

*‘‘Aphids are now a genuine problem for us unlike during the years when we started growing cotton in the district*. *Then*, *our seasons were still clear and predictable as it rained normally*. *Nowadays everything is different*. *The aphids are mainly promoted by lack of continuous rainfall during certain seasons*. *If there is a lengthy dry spell*, *for example three weeks*, *there will appear a lot of aphids*. *But*, *during those seasons when we get normal rains for this area*, *without those long dry spells which waste our money on pesticides*, *the populations we encounter will be normal*, *and we also do not use above normal amounts of pesticides to control them*. *The pesticides which we receive from the companies will be sufficient*, *but if the season is bad*, *we are obliged to purchase more pesticides”* (Respondent: TM 06).

There is an indication that during drought conditions, farmers use significantly higher amounts of pesticides to control aphids, as compared to seasons with normal rainfall patterns. Using increasing amounts of pesticides appears to be farmers’ own chosen way of adapting to climate variability in order to continue with cotton production. This approach is indicative of constrained adaptation capacity.

Another climate adaptive strategy which also appears to be contributing to increases in pest populations is the practice of keeping ratoon crops. These are regrown from the remains of root stock from the previous season. Very few farmers admitted to keeping ratoons because the practice is an offence in Zimbabwe[28]. However, several farmers indicated knowledge of ratoon cotton being maintained by other farmers. Field observations by CZ revealed cotton stalks in some fields during the month of November, three months after the deadline for cotton stalk destruction, possibly being kept for regrowth.

Certain researchers have shown that Zimbabwean smallholder farmers find ratoon cotton attractive for economic and climatic reasons [70]. It is cheap, as it saves seed costs, whilst it also ensures an early crop that establishes from the first rains of the season. Furthermore, the ratoon crop has a well-established root system, which ensures survival during seasons marked by low rainfall or severe mid-season droughts. In Rushinga, some interview responses, such as the following, indicate that perceived changes in the rainfall pattern are contributing to this practice:

*Yes*, *there has been a big change; pests have increased in their population*. *This is because of the changes in rainfall patterns*. *People are no longer cutting and burning their cotton stumps*. *“Those farmers who do not cut and burn these*, *end up maintaining their ratoon crops which are pests infested”* (Respondent: CZ 15).

The major concerns regarding the keeping of ratoon cotton crops relate to pests and diseases. Farmers who keep ratoons in Zimbabwe tend to control insect pests by using broad-spectrum pyrethroid pesticides early in the season. Pyrethroids in Rushinga district are regulated to be used between 25 December and 28 February. However, with ratoon crops, bollworms emerge early, prompting farmers to start using pyrethroids as early as in November, several weeks earlier than the recommended dates. When the pyrethroids are used early, biological insect control may be compromised due to the elimination of beneficial insects before they would have had the chance to predate on the pests, potentially causing farmers to respond by using higher quantities of pesticides.

## Conclusion

Study findings suggest a bi-directional causal relationship between increasing pest populations and increasing pesticide use due to a combination of factors including farmers’ perceptions and practices, the roles of government and the cotton industry as well as climate variability. Increasing pesticide use may result in higher incidences of pesticide exposures, which is of great concern for human health in many Low and Middle-Income Countries. Appropriate practices and policy measures for pest management and pesticide use reduction are, therefore, necessary to reduce potential pesticide-related health risks for farmers and their families. An Integrated Pest Management Policy for Zimbabwe is recommended to facilitate integration of chemical controls with a broad range of other pest control tactics such as preventive measures, cultural controls, biological controls, and host plant resistance. The major limitation of this study has been the lack of pesticide use data and local climate data for the area of study to verify participants’ observations and perceptions which inform their practices. As such, there is a risk that misperceptions about pests and pesticide use practices may be assimilated into local knowledge systems, and be passed on to future generations of farmers. There is, therefore, a clear need for continuous farmer education and awareness raising about the importance of scouting, using pesticides according to label instructions, and biological and cultural pest control techniques, including identification of beneficial insects.

## Supporting information

S1 Table Consolidated criteria for reporting qualitative studies (COREQ) checklist. (PDF)

S2 Table Interview guide. (PDF)

S3 Table Quotes exemplifying themes. (PDF)

S1 Text Interview transcript excerpts. (PDF)
