# Supplementary material for: Climate variability, perceptions and political ecology: Factors influencing changes in pesticide use over 30 years by Zimbabwean smallholder cotton producers
Source: PLoS One. 2018 May 10;13(5):e0196901. doi: 10.1371/journal.pone.0196901 (PMC5944972; doi:10.1371/journal.pone.0196901)
Supplement: S1 Table — (PDF) [file pone.0196901.s001.pdf]

S1 Table: Consolidated criteria for reporting qualitative studies (COREQ) checklist

Item	Guide Questions/Description
**Domain 1: Research Team and Reflexivity**	
**Personal Characteristics**	
Interviewer	Cliff Zinyemba (CZ)
Credentials	PhD Candidate
Occupation	Full time PhD student
Gender	Male
Experience & training	First involvement in questionnaire administration was in 2001. Training in Geography and Human Ecology at bachelor's and master's levels respectively
**Relationship with participants**	
Relationship established	No form of relationship with any of the study participants
Participant knowledge of the interviewer	Participants did not know the interviewer or research assistants prior to the interview
Interviewer characteristics	No characteristics were reported
**Domain 2: Study Design**	
**Theoretical Framework**	
Methodological orientation and theory	Thematic analysis. Themes were developed from data collected during interviews. Political ecology theory was used in the study
**Participant Selection**	
Sampling	Snowball sampling
Method of approach	Face-to-face
Sample size	50
Non-participation	None
**Setting**	
Setting of data collection	Data was collected at participants' homesteads
Presence of non-participants	No
Description of sample	At least 30 years of consistent cotton farming; Males (36), females (9) and couples (5)
**Data Collection**	
Interview guide	The questions were written by the authors. The questionnaire was pilot tested on 6 farmers.
Repeat interviews	No
Audio/Video recording	Audio recording was used
Field notes	Yes
Duration	30-50 minutes
Data saturation	Yes. Data saturation was reached when data categories were repeated in interviews. Coding was independently checked by co-investigators, AR and EA
Transcripts returned	Physical transcripts were not returned, but selected participants were contacted for clarification and interpretation of statements made
**Domain 3: Analysis and Findings**	
**Data Analysis**	
Number of data coders	One (CZ)
Description of the coding tree	Yes
Derivation of themes	Themes were derived from data collected
Software	Nvivo 11
Participant checking	No
**Reporting**	
Quotations presented	Yes
Data and findings consistent	Yes
Clarity of major themes	Yes
Clarity of minor themes	Yes
