# Supplementary material for: Climate variability, perceptions and political ecology: Factors influencing changes in pesticide use over 30 years by Zimbabwean smallholder cotton producers
Source: PLoS One. 2018 May 10;13(5):e0196901. doi: 10.1371/journal.pone.0196901 (PMC5944972; doi:10.1371/journal.pone.0196901)
Supplement: S2 Table — (PDF) [file pone.0196901.s002.pdf]

S2 Table: Interview guide

Discussion Topics	Examples of specific questions and probes
Pesticide use	1. What are the names of the pesticides that you have used on your farm in the past 30 years? (mention all those which you remember)<br><br>2. Has the amount of the pesticides which you use on your farm changed over the past 30 years?<br>i. Would you please explain how?<br>ii. What could be the reasons for these changes?<br><br>3. Are the quantities of pesticides that you are currently using enough to control pests on your farm?<br>i. (If not) what do you think are the reasons for this?<br><br>4. Have you ever used herbicides on your farm in the past 30 years?<br>i. (If yes) Do you remember the names of these herbicides?<br>ii. (If never or stopped using) Can you please tell me the reasons why?<br><br>5. Have you ever received training in alternative ways of controlling pests without using chemical pesticides?<br>i. (alternatively) Have you had any form of education or training about controlling pests?<br><br>6. Other than chemical pesticides, do you use other methods of pest management?<br>i. Would you please describe these methods?<br>ii. if farmer does not use any other methods) Are you aware of any other methods, even if you do not use them yourself?
Pest characteristics	1. What are the names of the cotton pests that are commonly found on your farm<br>i. if you do not know or recall any names, would you please describe them to me?<br><br>2. Have there been any changes in the types of pests on your farm in the past 30 years?<br>i. Would you please describe these changes?<br>ii. Have you noticed any new pests?<br><br>3. With which of these pests would you say you have had the most problems over the past 30 years?<br>i. Would you please describe the nature of problems you have had with these pests?

	3. Have you been noticing any changes in the amount of pests on your farm over the past 30 years?<br>i. Would you please describe these changes?<br><br>4. In addition to the issues already talked about, are there any other concerns you have about pests on your farm?
