# Supplementary material for: Climate variability, perceptions and political ecology: Factors influencing changes in pesticide use over 30 years by Zimbabwean smallholder cotton producers
Source: PLoS One. 2018 May 10;13(5):e0196901. doi: 10.1371/journal.pone.0196901 (PMC5944972; doi:10.1371/journal.pone.0196901)
Supplement: S3 Table — (PDF) [file pone.0196901.s003.pdf]

S3 Table: Quotes exemplifying themes

1. Change in pesticide use

Transcripts\\CZ 01
No, we are using plenty of pesticides.
It's the fault of us farmers. There are some things that we are not doing right. For instance, some farmers grow cotton, say this season, and after harvesting they do not destroy the cotton stalks. Others leave the stalks standing in the fields and they say they are keeping them to re-grow. The problem with these re-growths is that those farmers with these start their season with applying broad-spectrum pesticides, while I, in my adjacent farm start with dimethoate. So, the problem is that i may end up having to use plenty of pesticides because the adjacent farmer did not destroy the stalks and then i will start to encounter a lot of pests. That's a serious problem that we are having in farming. Farmers are not destroying their cotton stalks.
Transcripts\\CZ 02
Yes, we now use more herbicides, mainly because there is now a more serious problem with the herb, chaguduma
Transcripts\\CZ 03
The amount has increased
Transcripts\\CZ 05
There are changes now. These days we are now using more pesticides per hectare than we used in the past.
Transcripts\\CZ 07
But, i also realise that in the past in this field here i would need only two bottles, but nowadays i need up to 8 bottles. The amount of pesticides that i need now to control pests on the same farm has more than doubled.
Transcripts\\CZ 08
Well, we are now growing on a smaller portion. There are some who are now just doing regrows, which need a lot of pesticides. It's because they are attacked by pests early. It's not allowed, but sometimes people are tempted to just steal also.
Transcripts\\CZ 09
No, there is no difference, its only that in the past there were more pesticides made available to the farmers. Nowadays, if you want to get more pesticides the cost of them is rather prohibitively high. So, the pests are now getting their chance.
During the old days we used a lot of pesticides. Nowadays we notice that the amount of pesticides used is getting lower.
Because the price is getting higher and things are getting more expensive.
Transcripts\\CZ 12
No, it's just the same. It's just that changes in pesticide names have changed. In the past, the pesticide which i referred to as Oncol, used to be Rogor. Then it was renamed Dimethoate, Thereafter Oncol, Marshall, and then they gave us that powdered one which i talked about Acetamark.
Yes, the amount of pesticides used has not changed. Because, the dimethoate which we are talking about, we use number 30, the Oncol, we use the same number 30 and then the powdered one, we just use number 30.
Transcripts\\CZ 13
It's mainly because in the past there were fewer cotton farmers, so we could get as much pesticides as we wanted from the cotton companies with whom we had contracts. Nowadays there are now too many of us. We are now being rationed when it comes to receiving pesticides

being told that we do not have to always just spray anyhow, we must first survey the field and only spray when pests are spotted.
In the past, we used to use a significantly higher amount of pesticides as compared to nowadays
Transcripts\\CZ 15
We reached a point when we used a lot of pesticides, also depending on the size of cultivated land. But, it was also because of 'resistance' found in the worms. We started using a lot of pesticides.
Like i said, now the worms are very resistant. It's due to such factors as ratooning the crops. The worms go deep in the ground to the root of the plant as a way of dodging the pesticides. When we plant to cotton the plant will already be diseased. Mainly because of these worms and other infestations. We notice that in the past we would spray fortnightly after scouting for pests. And even after the two weeks the pests would not be too much. Nowadays it's different, after spraying today within three/four days you find there are pests.
It's the same pests that have just been there, only that they would be resisting.
Transcripts\\CZ 16
There have not been any changes; to me the amount i use per hectare has remained stable over the years.
Transcripts\\CZ 17
We now use less amounts of pesticides. The supply that is coming from the companies is now a bit crooked. In the past, once you are contracted, you knew that the company would give you all the inputs needed to produce on your hectare all at once.
Transcripts\\CZ 19
These days we use more pesticides than in the past.
Transcripts\\CZ 20
We are still using the same amount because they are still giving us the same number of bottles per hectare, but we observe that in the rate at which the pests are dying is now different. But, the measurements that we use for the concentration of pesticides in our knapsacks are the same as those which we used many years ago, nothing has changed.
Transcripts\\CZ 21
Yes. We have used less amounts over the years because we have also been receiving less. That is why i have resorted to killing the worms physically.
Transcripts\\CZ 23
Yes. We are now using more pesticides.
I think it's because people are now doing regrows. And these keep hosting pests. If the neighbour's farm has regrowths, pests from their farm will cross over to my own farm. It's because when they do not destroy their cotton stalks, pests will keep surviving in these stalks.
Transcripts\\CZ 24
Yes, in the past we used to apply a lot of pesticides compared to nowadays.
Yes, in the past we used to use many pesticides. Now, we know better. We now know at this stage i use that pesticide, at that stage i use this pesticide, and then towards the end i use Gukurahundi and again that Acetamark again. The reason why we use it towards the end again is that those aphids get into the cotton.
Transcripts\\CZ 25
No, there haven't been any changes. It's basically the same amount.
Transcripts\\TM 02
I think the pesticides are the same; it's only the names that have changed. The pesticides are still strong, it only calls for one's knowledge to use the pesticides.
Transcripts\\TM 04
No, they have not changed. Our only request is that they should make them stronger so that we can be able to kill pests.
Transcripts\\TM 05

These days there seem to be more pesticides
These days there are more pesticides, but they are weak, yet in the past we had fewer pesticides, but they were more powerful.
Transcripts\\TM 06
We are now using fewer pesticides. These days they are giving us just one bottle per hectare, for instance.
Transcripts\\TM 07
Yes, in the past we used more pesticides. The companies gave us our pesticides all at once, and there was no need to go and look for extra pesticides. Nowadays we are receiving fewer pesticides.
Transcripts\\TM 09
No, there is no change. I only see as if it’s the names that are changing. I see that it appears as if Fernkill, for example is simply changing the name, of its changing the percentage of how it kills.
Transcripts\\TM 11
I see that there is a change. In the past, we would receive all types of pesticides, and we would use and not finish it the whole season
Transcripts\\TM 12
Yes, we are now using more pesticides than we used in the past.
Transcripts\\TM 13
Yes, there is a change. We are now using fewer pesticides, and these no longer have power. They absolutely have power.
Transcripts\\TM 19
There are now more pesticides. But they are not effective in controlling the pests.
Transcripts\\TM 25
Yes. Nowadays there are fewer pesticides as compared to the past.
Transcripts\\TM 27
Nowadays there are fewer pesticides. In the past the pesticides had a lot of power.

2. Change in pest population

Transcripts\\CZ 02
Yes, the lady bird has increased its population. During the 1980s we didn’t encounter a lot of these.
No, there isn't any increase in pests, except that i notice more problems with the weeds.
Transcripts\\CZ 03
There seems to be an increase in the population of the red boll worm because some of the pesticides seem to have lost their power.
This is causing the population of the pests to increase. Therefore, if they made the pesticides a bit stronger, i thing we would, at least, be able to control the pests.
It's the increase in the population of worms. They never get completely destroyed, so we tend to continue spraying.
Transcripts\\CZ 04
Pests’ populations are increasing because people are no longer cutting their cotton stalks. Therefore, the pests are developing resistance because they stay there. They do not die because these stalks will not have been cut and burned. The worms will have stayed in the stalk such that when you attempt to kill it using pesticides it will not die.
Why pest types are increasing is that people are not taking care of their stalks. in the past we used to cut and burn our cotton stalks soon after harvest, and by September everyone would have burned their stalks. Nowadays, you find these stalks still standing in the fields. Therefore, the pests are just there. When we spray them, these pests would be very resistant. To say you have killed them, what sort of pesticide would you have used? The pesticides that we have these days we

always say they no longer work; of course, so, how would they if these pests are left to survive through summer growing season, winter and again another growing season?

Transcripts\\CZ 05

I think it's all got to do with the pesticides that we are now using. I think the pesticides are no longer as powerful as the pests which we used in the past. In the past the pesticides were so strong, even the smell was so powerful such that after spraying, one could not get into the field to weed because the smell would be so prohibitively strong. Nowadays it's so different; if you spray today, you may return tomorrow to weed the same field.

The worms are increasing in their population, particularly the red and the green ones.

I think it's because the pesticides are no longer as powerful as they were in the past.

Transcripts\\CZ 06

Right. There are significant changes. Pests are growing in their numbers. The pesticides that we are currently using are not powerful. Maybe it's because the worms are now used to the pesticides, i don't know? Therefore, they are not dying and are simply multiplying.

Not dying, but actually increasing in their numbers.

Transcripts\\CZ 07

Well, these have always been there. It's only when you delay spraying them that you think they are increasing. They need carbaryl to prevent them. So you must not delay. But, in general, these pests have not increased or decreased. I only notice that if there is a delay on my part i tend to encounter more of the pests.

Transcripts\\CZ 08

They are increasing

I think it's because of resistance that we are giving to these pests, because of the many types of pesticides that we use. You hear one farmer say i use this pesticide and another one saying they use that, and so on. But, we are not able to completely kill them.

Transcripts\\CZ 09

If we compare pests in the past with pests now there is a difference. I see that the way we live now is making it different. The pesticides that we use now are different. In the past, we would receive two buckets of cabaryl-85, and one bottle of Thiodan. During that time, i think we were getting more pesticides. Nowadays it seems the amount of pesticides are being reduced. They are now being overpowered by the pests. People nowadays are buying extra pesticides on top of what used to be the standard and what they receive from the cotton companies. Its different from in the past when they were just throwing pesticides at us for use. They didn't have much knowledge.

No, there is no difference, its only that in the past there were more pesticides made available to the farmers. Nowadays, if you want to get more pesticides the cost of them is rather prohibitively high. So, the pests are now getting their chance.

Transcripts\\CZ 10

Aphids are the ones which seem to have been growing in terms of their amount. For your crop to be a good crop, you must first win the initial battle against aphids. However, we do not always get the pesticides against these aphids in time. If you manage to get your pesticides against aphids in time, you know that you are growing your cotton very well, because carbaryl 85 will make your cotton look healthy with very good leaves, but `you will have sprayed for aphids.

Transcripts\\CZ 11

Yes, in the past they were fewer, and now they seem to have slightly increased in their population. Farmers are not getting the pesticides on time, they get them late that is why there end up appearing a lot of pesticides in the fields. The pesticides are not available in time. The issue is, farmers do not have the money to buy pesticides, and they wait for the pesticides which they get on credit from the cotton companies. But, the distribution of these pesticides is not efficient, they arrive late. Therefore, pests end up multiplying in the fields. In the past, when farmers signed for input loans with one company which was operating in the area then, they would receive all their inputs at once, before they started planting. When farmers received their pesticides before they even planted, they would control the pests once they appeared, and this managed pests to low

levels. This time, farmers receive pesticides when the pests are already appearing and normally they would have already lost several bolls to the pests.
Transcripts\\CZ 12
Yes. There are a lot of pests.
Yes, there are changes. The pest population has significantly increased because nowadays people are increasingly lacking money for buying seeds and are therefore resorting to ''regrowths'' of the old plants from the previous season. We are supposed to but slashing and burning these cotton stalks, which others are now growing as their new plants in a new season. Others only destroy their crops too late after the deadline for destroying and burning the cotton stalks. Therefore, these pests are harboured for a long time and they do not completely die. Therefore, they increase their population. When farmers favour regrowths, there is no longer crop rotation in the fields as they continue with the same crops in the same fields. It's now different from what the situation was like in the past when the agricultural extension officers would do inspections, encourage farmers to destroy cotton stalks and even fine those who flouted the regulations.
Transcripts\\CZ 13
The changes that i have seen is that even if you are spraying your crop very well, if there is an adjacent farmer who is not spraying his crop well, worms from his farm may migrate to your own crop. But if you are both spraying very well, the worms will be controlled.
Transcripts\\CZ 15
Yes, there has been a big change; pests have increased in their population. This is because of the changes in rainfall patterns. People are no longer cutting and burning their cotton stumps. Those farmers who do not cut and burn these end up maintaining their ratoon crops which are pests infested. By the time the rains come these worms will already be in the plant. Worse still, the type of pesticides which we were using the previous years are so ineffective, they just do not work and the cotton all gets eaten by the pests. Sometimes we do not get the pesticides in time, such that when we finally receive our pesticides it will be impossible to control the worms.
Transcripts\\CZ 16
I think pests are not increasing in their population. If you spray your cotton with lambda before the worms are present, they will die. But, if you delay and get in to spray after the red bollworm has entered the bolls, controlling it is very difficult. You will have to return next week and you might find it outside after it has finished consuming one plant. As long as it is inside the boll, it will not die because it will enclose itself inside.
Transcripts\\CZ 17
The increase in pest populations is because the pesticides are not able to kill.
The concern that i have is that the pesticides are not controlling the pests, so the population of pests is increasing.
Transcripts\\CZ 19
Yes, these have been increasing steadily over the years. The past two seasons have been the worst. They even do not respond to the pesticides.
I think it’s because the pesticides no longer have power.
I also think it’s because these cotton companies when they give input credits to us they also give us expired pesticides as they cannot afford to lose money by throwing them away. When we use these Pesticides will not work because they would have expired.
The long ones, the green ones and the yellowish ones are the ones that seem to be increasing in their numbers.
Transcripts\\CZ 21
In the past they were much fewer. In the recent years the pests have been increasing in population. It’s mainly because we haven't been receiving enough pesticides from the cotton companies. We have then resorted to moving in the field to physically capture the worms and then kill them using a wire. If you do not see the worm it can eat all the cotton bolls in the vicinity.
Transcripts\\CZ 22
It seems there have been increasing numbers of the red ones.
Transcripts\\CZ 23

Yes, in the beginning the red bollworm was not as much as it is now. The population numbers have significantly increased in the recent years. And there has also been changes in the types of pesticides used against it.
I think its because people are now doing regrows. And these keep hosting pests. If the neighbour’s farm has regrowths, pests from their farm will cross over to my own farm. It’s because when they do not destroy their cotton stalks, pests will keep surviving in these stalks. Last year but one, for example, we ended up observing that some plants that had already developed several bolls started to develop a disease that appeared like mutation of plant cells. After five days to a weak the plant would wilt and eventually just dry. Some say it is caused by the worms which would have survived in the ground. When we cut the plants right where there appeared to be diseased, we found that there were some small white worms.
Transcripts\\CZ 24
Let me take you back, in answering you. In the past when we were growing cotton, there was no talk of having to make cotton cuttings. Which they are also calling ''regrowths''. In the past, it was less prevalent. We would harvest our cotton, then cut the cotton stalks and burn them. Nowadays, people no longer cut their cotton stalks. They are now just having regrowths. The worms are now remaining in the soil. When it rains, the cotton stalks immediately shoot, and the worm would be there, already having laid some eggs. That is the reason why the red bollworm is increasing in its population. It is no longer responding to pesticides. It is now surviving during both the planting season and the off season.
Transcripts\\CZ 25
The American seems to have significantly increased.
Transcripts\\TM 01
The red spider, the American bollworm, and the red bollworm have increased their population.
Transcripts\\TM 02
I have not really noticed any changes
Transcripts\\TM 04
Yes, they are growing in their population. In the past they used to accuse us of destroying cotton stalks late, but in the recent years, I am always destroying my stalks even before I have sold my cotton, but I do not see any decline in population sizes. In fact, they are actually multiplying their numbers. They are now used to the pesticides that we use.
I wish we could receive our inputs timeously so that we can see if these pests are just there because they are not being targeted for destruction timeously, or because they have developed resistance.
Transcripts\\TM 06
There aren’t any significant changes as long as pesticides are available to kill them. But, if there are fewer pesticides the pests can increase.
Transcripts\\TM 07
I think they are getting fewer.
I think it’s because of the pesticides that we are receiving.
They are stronger, they are trying.
No, in the past they were much fewer, and i would always spray them and they would all go.
Transcripts\\TM 08
I see that the aphids have increased. The red bollworm has also increased. It’s because there are some, those of us who do not cut and burn their cotton stalks. The bollworm goes down that stalks and lays its eggs in the roots of the plant. When it rains these eggs will hatch, and it will become impossible for us to control it once it hatches.
Transcripts\\TM 09
The pests are actually increasing in population size.
Transcripts\\TM 10
Never the less, red bollworm is the one that seems to be increasing in population.
Transcripts\\TM 11
I see the green worms as having increased in population. These also eat the leaves of our crop.
Transcripts\\TM 12

My observation is that there is an increase in the population of the worms.
Yes, these days there are generally more pests in cotton than what it used to be like in the beginning.
Transcripts\\TM 13
Those that have significantly increased are those that are green. These have increased and they do not even respond to the chemical pesticides when sprayed on.
They have significantly increased, and they do not respond to the pesticides. They don't.
Transcripts\\TM 14
Yes, there are changes. I see that the aphid population is becoming lower. But, the worms that affect the bolls, i have realised that if you find the pesticides for them, they can be controlled, but if there is not enough pesticides, there can be a serious problem, cotton may not reach good harvests
Transcripts\\TM 15
There has been an increase in the population of the red ones
From the way i see it, i think they have not increased in population because there are now a lot of people who are now growing cotton.
Transcripts\\TM 16
The red bollworms, the American and the red spiders are increasing in population every year.
Yes, i would say over the past twenty years the pest populations have increased, and if we use the type of pesticides that we are receiving to control them they are not getting killed by these pesticides.
Transcripts\\TM 17
I have observed that there are more Red boll worms because people are not slashing and burning their cotton stalks as they should. That's the problem. If a worm is left alive this season, it will be impossible to control it next season because it would have developed resistance. It would not die when sprayed on. What i would want to encourage all farmers is to destroy their cotton stalks just the way tobacco farmers destroy theirs and burn them. Just the way our agricultural extension officers encourage.
There are now more pests as compared to in the past. They are actually keeping on growing in their numbers.
Transcripts\\TM 19
Yes, nowadays there are more pests
Transcripts\\TM 20>
These green and red worms are the ones which are increasing in their population.
Transcripts\\TM 21
Yes, since we started i am noting that the pests are increasing. This is because the pesticides which they are giving to us do not have power
Transcripts\\TM 23
The amount of pesticides is increasing because there are some farmers who are not taking care of their cotton stalks. The worms that result as a result of the ratoon crops are very deadly. They easily destroy the bolls.
No. there is a difference. In the past there were fewer red bollworms. These days there are now more of such because of the increasing practice of people not cutting the cotton stalks.
Transcripts\\TM 24
Well, in the past there were fewer pests, but there were many people who were growing cotton. Nowadays there are fewer farmers still growing cotton, and those growing are now growing on a smaller scale, but the pests are always appearing in larger quantities as soon as cotton is planted.
Transcripts\\TM 25
Aphids and red bollworms have increased in population.
Yes, they are increasing.
Transcripts\\TM 26
Yes, there are those which have increased in their populations over the past couple of years. They appear like grasshoppers, but they do not fly, they feed on the cotton (jassids).

Yes. Nowadays there are more pests because there is too much sun, and it is too warm.
Transcripts\\TM 27
Yes, there are more pests now than there used to be in the past.
Yes, there seem to be some changes.
In the past we would only spray with Rogor after our cotton had grown in height by several centimetres. But, nowadays, we need to spray it while it will still be very short near the ground.

3. Change in Pesticide effectiveness

Transcripts\\CZ 02
With insect pests the problem is with the pesticides. It's hard to understand if the the pesticides are still effective. I think they have reduced the strength of the pesticides. In the past we would spray this week, and then get back to the field next week. Nowadays if we spray today, we will return to spray the day after tomorrow. If you do not go back to spray within the same week you will find your crop totally destroyed when you go back.
I really think that the power of the pesticides has been reduced.
We used to use Carbaryl 85, then Rogor, then Thiodan and then *Gukurahundi*. These pesticides had their full strength! They were very strong; they could really kill. Malbak was really effective. Thiodan was banned completely.
Transcripts\\CZ 03
Well, its relatively effective, but at a very low rate.
I am referring to the power of the pesticides. The pesticides have low power. The pests die, but the ones that die are the ones that are sprayed while outside the cotton bolls. Those that manage to enter the cotton bolls are not easily killed.
We do not rotate pesticides. The ones we use year after year are all the same. As long as the pesticides have not yet expired one can always keep for the following season.
Transcripts\\CZ 04
The pesticides that we used in the past were very strong. We used to get 200ml, and we would use the same small bottle till we harvested. But, nowadays we have the types of Lambda, that's the pesticide whose name i had forgotten, the one that itches a lot; we get 500mls of it. But, just that one 500ml bottle is not enough. You need four/five for you to be able to harvest, which means the pesticides that we are getting nowadays are very weak, they do not have power.
I always keep for use the next season.
Transcripts\\CZ 05
Because the pesticides are no longer powerful, as powerful as they used to be. Under normal circumstances, if i spray today i should return for another round in two weeks time. However, these days it's taking only one week. If i go back to inspect my cotton next week i will find pests, so i will have to spray.
Yes, it is effective if you are not hesitant to use as much as you can. If you are hesitant and less generous with it you will lose your crop to the pests. You have to spray every week. If you skip just one week when you return the following week you will find all your crop destroyed.
ranscripts\\CZ 06
The pesticides that we are currently using are not powerful. Maybe it's because the worms are now used to the pesticides, i don't know? Therefore they are not dying and are simply multiplying.
In the past, we would only spray every other week, two weeks apart between spray periods, and you would go and scout and see very few pests present. But, the past few seasons, we have been spraying, and then on return the following day we would find pests still present. Not dying, but actually increasing in their numbers.
It is the power of the pesticides. That is what we have observed. We tried to maintain our measurements that we have always used in the past, but we are observing that either the pesticides have lost power or they still have power, but the pests themselves have developed resistance.

Were sometimes there may be changes, because the following day when i go and inspect my field i find some dead pests, but still, there will also still be those resistant ones which will still be alive.
Transcripts\\CZ 08
Yes, there are now many types, we hear there is this type and that type. But, from my experience Fernkill is the only one that is very helpful. The other ones do not help us in any way.
Transcripts\\CZ 09
They are now being overpowered by the pests.
The only problem that is see with regards to the pesticides that we buy is that when they expire they never tell us that these pesticides have expired. They just market knowing that their pesticides have expired. I only know that it's expired when i spray. Even when i try to increase the concentration of the pesticides, they don't work. It's only after close examination that i realise that we are buying pesticides that are expired. For example, that is the case with those companies from which we get loans. They never explain to us that the pesticides are expired. When we use the pesticides the worms will damage our crops because the pesticides will have no effect on them. There, that is where i have seen that there is a problem. They do not tell us that their pesticides are expired.
Transcripts\\CZ 10
Yes, its enough because the bottles of pesticides these days are a bit bigger; but the pesticides are not as strong as the ones which we used in the past. In the past the pesticides were much stronger. Even Thiodan which is now called Thiamex; there is no longer the Thiodan that used to be there in the past. That one was quite strong; if you used it there was never going to be a single worm found on your cotton.
Definitely, even a lot of other farmers agree that these days the pesticide manufacturers are 'limiting' the strength of the pesticides. Even the moths no longer have 'excuse' with the pesticides. We may wrongly say that they have now become resistant, and yet the pesticides are the ones which are now much weaker.
Transcripts\\CZ 11
Eh, yea. All the pesticides that we have talked about, especially the Gukurahundi which control the worms are in the same group; they work, they are effective. There are no pesticides that are ineffective.
Transcripts\\CZ 13
It's not enough because the strength of these pesticides is limited.
Transcripts\\CZ 14
It's to do with marketing. You see, if i was telling you this in business, i would tell you the truth, but not the whole truth, knowing that tomorrow you would return for the rest of the truth.
It's a deliberate thing. They are making the pesticides ineffective, so that farmers would return to buy more. But, the standards association of Zimbabwe would have tested and endorsed something else.
Yes, i am talking about the standards association of Zimbabwe, if things are not standardised they are subject to manipulation.
Yes, but they are subject to manipulation.
It's just similar to the bottled water that you buy and drink in Harare. It's got a standards association of Zimbabwe logo, but it is bottled straight from *Mukuvisi* River. If you drink it you will be struck by diarrhoea before you even reach Mazowe. Isn't it standardised. This is Zimbabwe.. Chuckles.

Transcripts\\CZ 15
The change is that they resist if we use the same type of pesticides. So, what we ought to do is that if we use Fernkill for two seasons, for example, we must change to Fenvalerate. If we use the same type of pesticide they will resist and not die.
Yes, at other times due to our ignorance and also just using the pesticides that are available to our disposal; but the consequence is that the pests will grow in their population.

We reached a point when we used a lot of pesticides, also depending on the size of cultivated land. But, it was also because of 'resistance' found in the worms. We started using a lot of pesticides.
Like i said, now the worms are very resistant. It’s due to such factors as ratooning the crops. The worms go deep in the ground to the root of the plant as a way of dodging the pesticides. When we plant to cotton the plant will already be diseased. Mainly because of these worms and other infestations. We notice that in the past we would spray fortnightly after scouting for pests. And even after the two weeks the pests would not be too much. Nowadays it’s different, after spraying today within three/four days you find there are pests.
Transcripts\\CZ 16
If you spray your cotton with lambda before the worms are present, they will die. But, if you delay and get in to spray after the red bollworm has entered the bolls, controlling it is very difficult.
No. What happens is, if we just spray anyhow, those pests will get addicted to the pesticides and in the long run they will not die. So, we first assess in the field for the presence of the eggs of the red bollworm. If we find them we then spray. If the eggs are sprayed they will be affected by the pesticides and they will not hatch.
Transcripts\\CZ 17
No, it’s not, particularly this one that they call lambda. It does not do anything to the worms.
No, it is something to do with the pesticide.
The increase in pest populations is because the pesticides are not able to kill.
The concern that i have is that the pesticides are not controlling the pests, so the population of pests is increasing.
Transcripts\\CZ 19
I think it’s because the pesticides no longer have power.
I also think it’s because these cotton companies when they give input credits to us they also give us expired pesticides as they cannot afford to lose money by throwing them away. When we use these pesticides they will not work because they would have expired.
I think the pesticides have lost their power.
Transcripts\\CZ 20
It's the same pesticides, but these days they use ‘’*zhing-zhong*’’ pesticides. The smell is the same, but the names have changed, and the power of the pesticides has also changed. Nowadays these pesticides do not have power.
we observe that in the rate at which the pests are dying is now different. In the past the pests died completely, but with the types that we are now using we still struggle because the pests always remain on the fields. That is why we often ask whether the new pesticides are now less powerful compared to the ones that we used in the past.
Transcripts\\CZ 22
The pesticides no longer have power. We had the pesticide called Thiodan, that one had so much power.
Transcripts\\CZ 24
Then we spray. The pesticide we used in the past seems to be outdone by the Acetamark in killing aphids.
In the past the pesticides were powerful, but the pests and diseases were also very few. Nowadays, the pesticides are powerful because they are changing the pesticides each year. The pesticides which we use this year are not the same ones that we will use next year.
I think we really need new pesticides that can effectively the red bollworm because the pesticides that we currently have are no match to this worm. When we spray, and then when we return to the field to scout, we find it, and then we end up looking for its presence and killing it manually.
Transcripts\\TM 01
I have observed that diseases are increasing due to the fact that the chemicals are now very weak on aphids.
Yes. In as far as i have observed, i am convinced that the chemicals that we are now using have lost their power. The chemicals are no longer suitable for the worms that are there. Could it be

resistance? I wonder! But, i have observed that even when i tried to grow cotton in a field that i had never before used for cotton, i realised that when i sprayed for pests expecting to return after 14 days, within three days i would notice the presence of pests, clearly meaning that they never died. And, that means that the pesticides are weak, they are not effective in killing these pests at all. Yes, there have been changes? The pesticides are now so weak.

Transcripts\\TM 02

Yes, they are. They are very strong. If you, as a farmer, think that the chemicals are weaker or not enough, then do not grow your crop on a large scale so that your chemicals can be sufficient.

Transcripts\\TM 05

These days there seem to be more pesticides, but these have no power to kill pests. We may spend the whole week spraying, but the pests will always remain. If i spray today and go back to my field tomorrow i will still find the worms present.

We are receiving sufficient pesticides in terms of quantity, but the pesticides are insufficient when it comes to their effectiveness to kill pests. They just don’t have power at all.

Transcripts\\TM 09

Yes, it appears these pests are now resistant to the type of pesticides that we use to control them. I used to think that the type of pesticides that we are receiving to control them do not have power any longer.

Transcripts\\TM 11

I observe that the pesticides that we used in the past had power. The current pesticides are less powerful.

Transcripts\\TM 12

It’s difficult really to know. In the past there were few worms, so maybe they were easier to control. So for us to really see if the pesticides are working or not is difficult.

Transcripts\\TM 13

Tthese no longer have power. They absolutely have power.

Transcripts\\TM 15

There is a difference, the current pesticides are effective, but not as much as the ones which we used in the past.

Transcripts\\TM 16

Lambdar is the pesticide which we are receiving these days, and my personal assessment as far as its effectiveness is concerned it that it is failing to control worms. But, if you make efforts to look for another type you see changes.

It’s changing. In the past we used strong powerful pesticides such as Thiodan. The pesticides which we are now using are less powerful, and they are failing to effectively control pest. In order to control the pests, it is best to control the eggs. If we fail to control the worms while they are still eggs, we may control to a certain extent, but not 100 percent.

Personally i have used an herbicide called Cotguard. I noticed the results that it is a very effective herbicide.

Transcripts\\TM 17

This is so because Lambdar is no longer effective in controlling these pests. It’s because we are using the same Lambdar on the same worms over many year that is why it is now resistant.

In the past the pesticides had so much strength. Those were real pesticides. If you sprayed with Karate, nothing would be left alive in the field. Karate was deadly.

Well, the type we are getting such as Lambdar are delivered to us already expired.

Transcripts\\TM 19

There are now more pesticides. But they are not effective in controlling the pests. It’s mainly because we are receiving our pesticides late after pests have already attacked our crops.

Transcripts\\TM 20

Yes, there are differences. We now use more pesticides than we used in the past. In the past we used only two types of pesticides which were very strong. Nowadays there are more pesticides which are no powerful at all.
Yes, they are killing, but they are not as effective in killing worms. The pesticides have not enough power to kill the worms.
Transcripts\\TM 21
Yes, since we started i am noting that the pests are increasing. This is because the pesticides which they are giving to us do not have power
Yes, these pests feed on the crops a lot. If you spray, say today, and go back to inspect tomorrow, you will still find them alive. They don't die.
Transcripts\\TM 23
In the past we used to receive enough, but these day there is just more but they are not working.
Transcripts\\TM 24
Nowadays there are more pesticide varieties, but the way they work or their effectiveness is different.
The pesticides may be enough, but their problem is they ineffectiveness when it comes to killing the pests. Some pests die, others do not.
Because the pesticides will be without any power; they will be weak.
Transcripts\\TM 27
In the past the pesticides had a lot of power.

4. Change in pesticide sufficiency

Transcripts\\CZ 01
Well, they tell us to use a single bottle of pesticides on one hectare. They give us very little pesticides. We are not getting enough pesticides from the cotton companies where we have contracts. So, we end up buying extra bottles of pesticides. If i just relax and rely on the pesticides that i receive from the cotton companies i will lose everything.
Transcripts\\CZ 04
The pesticides that we used in the past were very strong. We used to get 200ml, and we would use the same small bottle till we harvested. But, nowadays we have the types of Lambda, that's the pesticide whose name i had forgotten, the one that itches a lot; we get 500mls of it. But, just that one 500ml bottle is not enough. You need four/five for you to be able to harvest, which means the pesticides that we are getting nowadays are very weak, they do not have power.
No, it’s not sufficient. We really need more pesticides. At times when we scout and see that there are pests, we just ignore and pretend as if we do not see anything so that we conserve our pesticides so that they can last us the season. However, under normal circumstances, cotton should always be sprayed at the sighting of pests. This is because pests do not stop eating our crop, day and night. But, because the pesticides are not that plenty, we have no other choice than to just pretend as if we do not see that there are pests.
Transcripts\\CZ 07
It’s actually not enough because i cannot completely destroy the pests.
Transcripts\\CZ 09
The pesticides that we use now are different. In the past, we would receive two buckets of cabaryl-85, and one bottle of Thiodan. During that time, i think we were getting more pesticides. Nowadays it seems the amount of pesticides are being reduced
People nowadays are buying extra pesticides on top of what used to be the standard and what they receive from the cotton companies. Its different from in the past when they were just throwing pesticides at us for use. They didn’t have much knowledge.
Well, the pesticides are enough, if you have your money. Because there can never be a shortage of pesticides. There is plenty of pesticides.

Transcripts\\CZ 10
There is a change. In the past, when we were being loaned, we would get enough pesticides for the whole acreage, if you decided to buy on your own that is when you would notice that your crops would be attacked by pests. That is why we would loan. We would get the whole kit. If, for example you got a loan for two hectares, per hectare they would give you two bottles of Gukurahundi' Rogor two, and two packs of Carbaryl 85.
These days the quantities have declined. They no longer give a full set.
Transcripts\\CZ 12
Yes, it is enough. It's similar to overdosing a goat that you want to treat for worms. If you overdose what do you expect from it?
Transcripts\\CZ 16> - § 2 references coded [1,39% Coverage]
But, the cotton companies are the ones that tend to limit the amount of pesticides that they give to people.
Yes, i always find it sufficient.
Transcripts\\CZ 19
Usually the amount of pesticides that they give us are not enough, so we end up going to buy extra bottles. In the past where we used three bottles we are now using double that amount, but the worms are still not getting finished.
Transcripts\\CZ 21
It's mainly because we haven't been receiving enough pesticides from the cotton companies.
Transcripts\\CZ 22
In the past when we got input loans, we would get enough pesticides. If i got a loan for 2 hectares and then decided to grow three, then it would not be enough. In the past, they would give us enough. Nowadays they no longer give enough pesticides.
Transcripts\\CZ 24
Yes, but if used properly. The problem that we face is that we grow cotton on 2 hectares, and then receive only two or three bottles of pesticides which are not sufficient.
No. But, if you follow instructions and use the pesticides properly everything will go well because on 1 hectare you must use 2 packs of Acetamark, then 2kgs or Carbaryl 85, and three bottles of gukurahundi. That should be sufficient, if used properly.
Transcripts\\CZ 25
Yes, they are enough, if scouting is done well. There are actually even some left overs. Pesticides are not to be sprayed weekly even when there are no pests.
Transcripts\\TM 02
Those with enough pesticides are able to kill and destroy pests. if you do not have enough pesticides you will not win the battle against the pests.
Transcripts\\TM 04
Pesticides have been reduced. These days we are given only two bottles of Fernkill per hectare. But, the proper way to do it is that they should give us three to four bottles per hectare.
Transcripts\\TM 05
We are receiving sufficient pesticides in terms of quantity, but the pesticides are insufficient when it comes to their effectiveness to kill pests. They just don't have power at all.
Transcripts\\TM 06
No, we are not having enough.
Transcripts\\TM 08
No, they are not sufficient because i limit my spraying. I try to skip a week between my spray intervals. But, when i skip a week maybe it rains, and then when it rains my pesticide is washed off, and then the worms will eat my crop.
Transcripts\\TM 09

Since i usually get my inputs on credit under contract farming arrangements, sometimes the pesticides are not enough because the company giving the credit may give not enough pesticides and it may also give after long intervals

Transcripts\\TM 12

They are sufficient if you get a loan contract with a company that supplies enough pesticides. If you get a loan with a company that supplies pesticides that are not enough you end up selling livestock in order to buy extra pesticides.

Transcripts\\TM 13

No, they are not. We actually have to buy extra pesticides because the amounts that we receive are not sufficient.

Transcripts\\TM 14

Yes, they can be sufficient, particularly if you have enough pesticides. Only that, what is happening now is that the companies from which we get inputs on contract are the ones who are messing up because of the way they are giving us pesticides. We are not able to destroy the pests because they are giving pesticides that are not in tandem with the length of the season.

The pesticides are finished before the cotton bolls have even opened up. That is when the worms attack our crops.

Transcripts\\TM 15

No. We usually get our pesticides on credit from the cotton companies; so when i get once the companies may just tell me that the pesticides which i collected are already enough, even though i would still need more pesticides to control the pests on my farm. That is the problem that we face which makes the amount of pesticides used on the fields become insufficient.

Transcripts\\TM 16

No, they are not enough because at times we lack the financial means to buy sufficient amounts of pesticides. In fact, that is the biggest problem.

Transcripts\\TM 21

They have been limiting the amount they have been giving us.

Nowadays we are now receiving fewer pesticides and they are not enough. By the time we harvest all our pesticides would have been finished.

Transcripts\\TM 22

No, they are not enough, particularly if you get your inputs on credit.

Transcripts\\TM 23

In the beginning, we were getting good pesticides. They would give us Rogor, carbaryl, Thiodan and gukurahundi. They would give us about four or 5 different types.

Transcripts\\TM 25

No, they are not enough. For those of us who are on contact farming who receive inputs on credit, we always get pesticides that do not match the acreage. Sometimes we end up having to buy on our own or risk losing the crop to worms.

Transcripts\\TM 26

No, they are not enough. Under normal circumstances, per hectare i should use two bottles of Rogor, two bottles of Fernkill, and two bottles of Carbaryl. But, nowadays they give us only one bottle, and this is not enough, resulting in our cotton becoming susceptible to pest attacks.

Transcripts\\TM 27

No, they aren't enough. You may spray but soon after spraying you will still find pests.

5. Practices increasing pesticide use

Transcripts\\CZ 05

No, we do not use any pesticides on the other crops, except on cow peas which is usually attached by aphids. We spray Acetamark on cow peas before and after they have developed pods.

we use it because we know that it works against aphids.
Transcripts\\CZ 06
Well, it may be enough if i use it following the instructions written on the label. I follow the label directions. However, the pesticides are not effective in killing the pests
I double the concentration of the pesticides. If the label directions say that i should put only 30 mls in my 15 litre knapsack, i simply double and make it 60mls.
Transcripts\\CZ 11
No, i do not use any pesticides on other crops, except only in cotton
Transcripts\\CZ 12
You will use measurements such as Number 30 if it has grown too tall. Say the 1st of February comes but the companies from which loans are applied for have not delivered, and you have Carbaryly-85, you take cup number 30 and mix 10 cups in 50 litres of water. This will work in the place for Fernkill or Fenvalerate.
Well, it's nothing but ignorance. Not knowing the right pesticide to use. You see a farmer getting in the field to spray dimethoate because they may have spotted American bollworms. When they see that the worms are still there after a couple of days, they take the same Dimethoate and Fenvalerate and mix, to make a cocktail, with the belief that if they mix the pesticide will become stronger and kill the worms. They will be doing it wrongly.
Transcripts\\CZ 23
Yes, in groundnuts we often encounter aphids. We spray them with a pesticide.
Transcripts\\CZ 25
All that i can say is that, you see. If you take a drum of red sea and pour one shovel of sugar in it, you will not be able to taste the sugar. What i have observed is that farmers are a bit too ignorant; when the agricultural extension officers tell them to mix a certain amount of pesticides with 15 litres of water, they always make mistakes. But, if a farmer makes the right mixture, the pests will die.
Transcripts\\CZ 16
If you spray your cotton with lambda before the worms are present, they will die.
