# Supplementary material for: Climate variability, perceptions and political ecology: Factors influencing changes in pesticide use over 30 years by Zimbabwean smallholder cotton producers
Source: PLoS One. 2018 May 10;13(5):e0196901. doi: 10.1371/journal.pone.0196901 (PMC5944972; doi:10.1371/journal.pone.0196901)
Supplement: S1 Text — (PDF) [file pone.0196901.s004.pdf]

S1 Text: Interview transcript excerpts

CZ 01

Which pests do you encounter on your farm?

We first encounter aphids when it is hot. These days the pesticides are so different. Dimethoate, or Rogor is no longer effective in killing pests. It no longer has power. In the past we used *Shahsa*, you see! 'Onkoll' and it was very effective in killing aphids.

What other pests do you encounter?

Worms; The red ones, and the other ones! They eat our cotton.

Are there any other pests?

I would say these are the ones, when it comes to those pests that eat our cotton. But, you know, cotton also needs fertiliser.

What is the name of the weed that is most problematic for you?

There is *'chinzungu'*, that one is very difficult. Even when we spray it with pesticides, it does not die easily.

Is there a particular pest which you would say has been most problematic pest in the past 30 years?

It's the Redboll worm because it can easily multiply; it lays plenty of eggs. If it enters the boll, the moment it leaves be assured that it has laid its eggs within that boll.

Would you say there have been any changes in the past three decades in terms of the population of pests?

In the past we used to grow a lot of cotton. Well, i would say our harvest has dramatically been reduced.

What is causing that reduction in your harvest?

I believe it is because they are giving us pesticides which do not have power. I might be able to grow cotton on a very big piece of land spraying very well, but the problem is that the worms are not dying.

Have there been any changes in pest types in the past thirty years?

There are certain flying insects that have a very foul smell. They are also found in tomatoes sometimes.

What colour are they?

They are blue. They fly. If they pee on your white cotton you will not get a good grade.

So, are you saying these pests were not seen in the past, but you now see them often these days?

Yes, in the past this type was not there.

Are there any other concerns as far as pests are concerned?

Yes, the issue is only about spraying these pests, because our cotton will have already turned white, so our task will be to spray them so that they do not affect the grade of our cotton.

And the weed that you referred to, *chinzungu*, did you have problems with it in the past?

No, we didn't have any serious problems with it; it's really just a recent problem.

How do you control the weeds?

The only way to deal with it is to weed it using hoes.

Do you not use any pesticides against it?

No, even if you use any herbicides against *chinzungu*, it will not die. It's very difficult to control.

Are there any other crops which you grow other than cotton?

Yes, I also grow maize, *mhunga* and *mashava.*

Do you use any pesticides on these crops?

No! We only use fertilisers. But, in *mhunga* we do not use any fertiliser.

Has the acreage of cotton changed over the years relative that of the other crops?

In the past we used to dedicate much of the land and resources to cotton. But, nowadays we are growing less cotton and expanding the acreage of the other crops, maize, mhunga, mashava and even ground nuts.

Which pesticides do you use in cotton?

I use Fernkill, Dimethoate, also known as Rogor, and then Carbaryl-85, Thiodan is no longer being used these days. We used to use that powdered Thiodan in the past.
Why is Thiodan no longer in use?
I really do not know.
And, what do you use against weeds?
There is a new type of herbicide, whose name i do not recall.
Do you often change pesticides in different seasons, or you use the same pesticides season after season?
Well, cotton pesticides never change, no matter what. If they decide to change the type of pesticides where we buy them, we will just know that this type is *Gukurahundi*, and then that one is Carbaryl - 85, and this is the one to use first, and so forth and so forth.
So, they normally do not change your pesticides, is that what you are saying?
Yes, they just continue to give the same pesticide, for example Thiodan is no longer being sold, so we now know that there is the Fernkill.
What do you do with left-over pesticides at the end of the season?
I always examine the expiry date of the pesticides. If it has expired, i always throw away. But if it's not yet expired i keep it for use the next season, knowing that it's not yet expired.
Would you say these recent years you are now using more or less pesticides compared to three decades ago?
No, we are using plenty of pesticides.
What might be the reasons for using such a lot of pesticides?
It's the fault of us farmers. There are some things that we are not doing right. For example, some grow cotton, say this season, and after harvesting they do not destroy the cotton stalks. Others leave the stalks standing in the fields and they say they are keeping them to re-grow. The problem with these re-growths is that those farmers with these start their season with applying gukurahundi, while I, in my adjacent farm start with dimethoate. So, the problem is that i may end up having to use plenty of pesticides because the adjacent farmer did not destroy the stalks and then i will start to encounter a lot of pests. That's a serious problem that we are having in farming. Farmers are not destroying their cotton stalks.
Are you finding the pesticides which you are currently using sufficient to kill the pests which you encounter on your farm?
Well, they tell us to use a single bottle of pesticides on one hectare. They give us very little pesticides. We are not getting enough pesticides from the cotton companies where we have contracts. So, we end up buying extra bottles of pesticides. If i just relax and rely on the pesticides that i receive from the cotton companies i will lose everything.
Other than pesticides, do you use any other methods of integrated pest management?
No, i solely rely on pesticides and nothing else.
Have you ever had any training on IPM?
No, never
Thank You for your participation

CZ 02
Q: Which pesticides are commonly found on your farm?
R: There are moths, red bollworms, American bollworms, aphids and the ladybird beetle.
Q: Since around 1982, which, among these pests, has given you the most problems?
R: Worms are the most problematic. Whenever we grow cotton we have to be prepared to deal with these.
Q: Is there a type, among these pests that has changed in its population size?
R: Yes, the lady bird has increased its population. During the 1980s we didn't encounter a lot of these.

Q: Is there one particular pest that was not present when you started growing cotton, but is now being sighted?
R: It's actually these types of beetles. In fact, there are actually two types. The one with the red colour and the other one which is green-like.
Q: And, when it comes to weeds on your farm, is there a type of weed that's problematic?
R: Yes, There is *chidhongi,* which is also known as *chaguduma,* and another type called *chinzungu* because its leaves resemble those of groundnuts and another type called *chenamoyo*.
Q: Are there any other crops which you grow in addition to cotton?
R: Yes, alongside cotton we also grow maize and groundnuts.
Q: Have there been any changes in the acreage of cotton relative the acreage of these other crops over the years?
R: In the beginning we were growing a lot of cotton. We had so much faith in the crop. But now, due to the problems associated with the market price of cotton, we have decided to grow maize for food. It's much better.
Q: Which pesticides do you use on your cotton?
R: We normally use 85, Acertamak, Fenkill, and Lambda
And which herbicides do you use to control weeds?
I use atrazine
Has the amount of pesticides you use per hectare changed since you started farming?
Yes, we now use more herbicides, mainly because there is now a more serious problem with the herb, chaguduma
And as far as pests are concerned …?
No, there isn't any increase in pests, except that i notice more problems with the weeds.
With insect pests the problem is with the pesticides. It's hard to understand if the the pesticides are still effective. I think they have reduced the strength of the pesticides. In the past we would spray this week, and then get back to the field next week. Nowadays if we spray today, we will return to spray the day after tomorrow. If you do not go back to spray within the same week you will find your crop totally destroyed when you go back.
I really think that the power of the pesticides has been reduced.
We used to use Carbaryl 85, then Rogor, then Thiodan and then *Gukurahundi*. These pesticides had their full strength! They were very strong; they could really kill. Malbak was really effective. Thiodan was banned completely.
Why was Thiodan banned?
They say it was very strong such that when domestic animals ate any plant remnants they would die.
Do you use other methods of pest control other than pesticides?
I use herbicides to kill the weed, chaguduma, but i also use my hoe to weed. Where there are plenty of weeds the herbicide, Atrazine, is not effective enough. So we always use our hoes for weeding, a lot.
Have you ever received any training on integrated pest management?
Our agricultural extension officers are the ones who normally teach us.
You said the pesticides are no longer strong..?
Oh, yes, their strength is now greatly reduced
Do you cut and burn your cotton stalks each and every year?
Yes, we do. But the problem is that some farmers are now doing 'regrows' in order to cut costs on buying seeds. In the past nobody was doing regrows because there were lots of inspections and arrests.
By the 15th of August every farmer would have cut and burnt their cotton stalks, and those would not have destroyed theirs by September would be fined.

Thank You

CZ 03

Which pests are commonly found on farm?

I normally come across worms. These are of two types; the American Bollworm and the Red bollworm. There are also aphids, but they are not in large population.

Which pest would you say has given you the most problems over the past years?

Red bollworms are the most problematic.

Have there been any changes in the red bollworm population over the past two to three decades?

There seems to be an increase in the population of the red boll worm because some of the pesticides seem to have lost their power.

In addition, the number of farmers who are now growing cotton seems to have increased. What effect does that have?

This is causing the population of the pests to increase. Therefore, if they made the pesticides a bit stronger, i thing we would, at least, be able to control the pests.

Are there any new pest sightings?

Yes, i would say, there might be new pests because in the past the pests were much fewer. There might actually now be new worms that we just do not know.

But, have you made any sightings of a new pest?

I wouldn't want to lie, i have never come across any new pests that are different from those that we have always known.

So, you said the population is the main change?

Yes, it’s the pest population. No matter how much you spray, they never go.

Are there any other concerns regarding pests on your farm?

No.

Which other crops do you grow?

I also grow maize, ground nuts and *mashava*

Looking at the acreage of cotton relative that of these other crops, have there been any changes in the recent years?

Yes, my cotton acreage has dwindled relative to the other crops. I am cutting down on cotton because there is no water. Water shortage is the main cause. Cotton must be planted early. If you do late planting you will lose all of it to livestock. The rains are no longer reliable. Whan it rains the rainfall is sporadic.

What are the names of the pesticides that you have used on your farm?

There is Fenvalerate, karate, lambda, Acetamark, then Dimethoate, and others that i do not recall at this moment.

Which of these do you use on the worms?

I normally use lambda, Fenvalerate, Dimethoate, Acetamark, and i have also mentioned those that are also used on aphids.

Why do you use so many pesticides?

Well, some of the pesticides that we used in the past are no longer available; we do not see them anymore.

Has the amount of pesticides used on your farm changed from the time you started back in 1986?

The amount has increased

What might be the cause of this increase?

It's the increase in the population of worms. They never get completely destroyed, so we tend to continue spraying.

Now that your amount of pesticides has increased, has there been a corresponding increase in pesticide effectiveness?

Well, its relatively effective, but at a very low rate.

What do you mean by the 'rate'?

I am referring to the power of the pesticides. The pesticides have low power. The pests die, but the ones that die are the ones that are sprayed while outside the cotton bolls. Thos that manage to enter the cotton bolls are not easily killed.
So, if you say you should be spraying every week, does that mean you do not scout?
No, we scout. But, the problem is that these pesticides do not have power any longer. That is why we now know that we should spray, nearly every week.
Do you use other methods of pest control?
No, there are no other methods that can be done.
And weeds?
With the weeds i often do manually, but the past two seasons i have been using herbicides because i have just been able to afford them.
Have you ever had training on IPM?
We are taught how to use our pesticides. There are different measurements that come with different cups. We use different cups for different sizes of the cotton. We start with smaller cups and then use the largest cups when the cotton has grown big and when there are also a lot of pests. The cups are put in 15 litres of water.
Is this something that you were taught, or it's by experience?
This is what our agricultural extension officers teach us.

Thank You for your Participation

CZ 04
Which pests are commonly found on your farm?
The main ones are red spider mite, the American bollworm and the red bollworm.
With which of these have you had most problems over the past years of your cotton growing?
It's the red spider mite because the pesticides to use against it are not readily available. We no longer find the pesticides to use against it. Once it have entered your field you are done.
Oh, so the main issue is that the pesticides are no longer found?
No. They are there, we can find them, but the problem is that they are no longer powerful enough to kill. In the past we would use three bottle of liquid pesticides and one bag in powdered form. Once spotted, we would just carry our knapsack and bomb it. Nowadays, it seems the population is increasing. It's a real problem.
Have there been any changes in the amount of pests on your farm in the past 30 years?
Pests' populations are increasing because people are no longer cutting their cotton stalks. Therefore, the pests are developing resistance because they stay there. They do not die because these stalks will not have been cut and burned. The worms will have stayed in the stalk such that when you attempt to kill it using pesticides it will not die.
Are there any changes in the types of pests?
Yes, there are also new pests. It's only that i do not know some of the names. But, the ones that are known are the likes of red spider mite, red bollworm, and the American bollworm. Some of the newer pests, i don't know their names. There is one that is green-like with yellow stripes. One of the worms is called *kambasha* after the colours of the bus that used to ply our Rushinga route many years ago. There are also new types of aphids that are now colonising our fields whose specific names we are not sure of. Then there are other pests that are striped that cut the cotton, but the names are the problem for me.
What might be the cause of these changes?
Why pest types are increasing is that people are not taking care of their stalks. in the past we used to cut and burn our cotton stalks soon after harvest, and by September everyone would have burned their stalks. Nowadays, you find these stalks still standing in the fields. Therefore the pests are just there. When we spray them these pests would be very resistant. To say you have killed them, what sort of pesticide would you have used? The pesticides that we have these days we always say they

no longer work; of course so, how would they if these pests are left to survive through summer growing season, winter and again another growing season?

Looking at weeds; what are the main types of weeds that are found on your farm?

There is a weed that is whitish in colour. We manually weed it and throw it away. Other common types of weeds that we encounter are *bwerere*, and chaguduma, and *moyochena*. Then there is another one called chinzungu, which is the most problematic for cotton farmers.

What other crops do you grow?

I also grow maize and groundnuts.

Do you ever mix your crops in the same fields?

No, we cannot mix cotton with any other crops because of the pesticides we use. If we mix with cucumbers, for example, children might go and pick them and eat unbeknown to them that there are pesticides on the cucumbers. Our agricultural extension officers always remind us that we should never mix crops.

Has the acreage of cotton changed relative to other crops?

The land needed for cotton is always larger, if you are to realise a good harvest. You need at least 3hectares.

How much do you normally harvest per hectare?

Well, in the past when we used to get enough fertilisers we used to realise as much as 6/7 bales per hectare. Nowadays, because we no longer receive enough fertilisers, our harvests have declined. We are now realising just enough to survive, just about 2/3 bales per hectare.

Which pesticides do you use on your farm?

We first use Dimethoate, or Mospilan. It’s still the same pesticide, only that Mospilan is in powdered form. We use it against aphids.

When the plant starts to flower, we use Carbaryl 85. This is used against red bollworm.

Thereafter, we use karate, or this other one that is itchy. I do not remember the name, but it is in the same family as karate. It is very itchy.

Has the amount of pesticides used over the past thirty years changed?

The pesticides that we used in the past were very strong. We used to get 200ml, and we would use the same small bottle till we harvested. But, nowadays we have the types of Lambda, that's the pesticide whose name i had forgotten, the one that itches a lot; we get 500mls of it. But, just that one 500ml bottle is not enough. You need four/five for you to be able to harvest, which means the pesticides that we are getting nowadays are very weak, they do not have power.

Now that you are using more, would you say you are having sufficient to kill your pests?

No, it’s not sufficient. We really need more pesticides. At times when we scout and see that there are pests, we just ignore and pretend as if we do not see anything so that we conserve our pesticides so that they can last us the season. However, under normal circumstances, cotton should always be sprayed at the sighting of pests. This is because pests do not stop eating our crop, day and night. But, because the pesticides are not that plenty, we have no other choice than to just pretend as if we do not see that there are pests.

Do you use any pesticides to control weeds on your farm?

No, i do not. I always weed manually. I once tried using herbicides, but i realised that i have little knowledge about them.

Do you use any other forms of pest control?

No. I only use pesticides because there is no other way that the pests would die except by the use of pesticides. They are only killed by pesticides.

Have you ever received education of the usage of pesticides?

We receive education from our agricultural extension officers.

Thank You

CZ 05

Which pests do you commonly come across on your farm?
There are aphids, then some round boll worms that get into the bolls, and then there are the American bollworms.
Which is the most problematic?
I think all of them are very problematic.
But i think the aphids are very problematic. But, including the worms as well.
Since 1984, have you noticed any changes in pest populations?
Yes, there are changes. There are now many pests?
What do you think is causing an increase in these pests?
I think it's all got to do with the pesticides that we are now using. I think the pesticides are no longer as powerful as the pests which we used in the past. In the past the pesticides were so strong, even the smell was so powerful such that after spraying, one could not get into the field to weed because the smell would be so prohibitively strong. Nowadays it's so different; if you spray today, you may return tomorrow to weed the same field.
Are there any changes in the types of pests?
Uhmm, there is one green moth. That one cuts the terminal bud of the cotton plant.
Is this the same moth that's got a nasty smell?
No, it's not. This particular one is only found during the rainy season.
Have you communicated to the agricultural extension officers about this moth?
No, i never informed about it since it's a moth that's found everywhere; i just assume that it might just have been missed by the pesticides. It does not always cut the terminal buds, but it just happens that when it gets to a plant it may cut. This is not a fulltime cotton pest, if i may say.
Now, with regards to the population, would you say that the population of pests is changing?
The worms are increasing in their population, particularly the red and the green ones.
What do you think is the reason for the increase in the population of these worms?
I think it's because the pesticides are no longer as powerful as they were in the past.
Which types of weeds do you come across in your fields?
We normally have problems with *chenamoyo*, chaguduma, and *chinzungu*, and *chenamoyo* which prick when you touch or walk on it.
Do you use any herbicides for weeds?
We never afford to buy the herbicides, so we always weed manually.
Which other crop do you grow?
We also grow ground nuts, maize, cow peas, *mashava*, and *mhunga*.
Do you use any pesticides on the rest of the crops?
No, we do not use any pesticides on the other crops, except on cow peas which is usually attached by aphids. We spray Acetamark on cow peas before and after they have developed pods.
Is it the same one that you use in cotton?
So, when you use it on your cow peas, is it because your agricultural extension officers recommend it, or it's simply because you know that it works against aphids?
Laughter ... we use it because we know that it works against aphids.
In cotton, which other pesticides do you use?
There is Fenvalerate, Carbaryl 85, Fernkill, Thiamex, Thiodan,
Is the amount of pesticides used on a hectare the same as that used in the past?
There are changes now. These days we are now using more pesticides per hectare than we used in the past.
Why are you using more nowadays?
Because the pesticides are no longer powerful, as powerful as they used to be. Under normal circumstances, if i spray today i should return for another round in two weeks time. However, these days it's taking only one week. If i go back to inspect my cotton next week i will find pests, so i will have to spray.

So, you now use more pesticides. But, are these pesticides that you are now using more of, effective enough to kill and control the targeted pests?
Yes, it is effective if you are not hesitant to use as much as you can. If you are hesitant and less generous with it you will lose your crop to the pests. You have to spray every week. If you skip just one week when you return the following week you will find all your crop destroyed.
So, are you telling me that you just spray even when you have not scouted and found any pests?
You actually see the pests. Usually, when you see those moths flying in your field, it means that they are laying their eggs. So you should immediately spray.
Have you ever farmed on contract, getting input loans from cotton companies?
I did that a couple of years ago. Nowadays I'm doing it on my own
Alright, when you used to do contract farming, did you ever receive any protective clothing from the companies?
That is the farmer's own business. What concerns those companies is that the farmer has received the inputs that he needs to grow cotton. Finding protection is the farmer's own business. Even the companies themselves, never entertained the discussions regarding protective clothing for farmers.
But, do you realise that is is your right to receive protection if you are getting into a contract to produce them their cotton?
Yes, its our right, but our enemy is our ignorance. We never ask for protection even though we know that we are potentially putting ourselves on the path to the hospital.
Do you get any information on alternative pesticides or on how to use pesticides effectively and protecting your health?
Yes, we get this kind of education from our agricultural extension officers when we go for our meetings.

Thank You for your time

CZ 06
Which pests do you come across on your farm?
We normally deal with red bollworms, American bollworms, aphids, and moths. After these we then face a problem of red spider mite. This results in a disease that affects mainly the colour of the leaves and they start to fall off.
Which of these is the most problematic?
The American bollworm is the most problematic. A single worm can destroy several bolls in a very short space of time.
Over the past two to three decades, are there any noticeable changes in as far as the population of pests is concerned on your farm?
Right. There are significant changes. Pests are growing in their numbers. The pesticides that we are currently using are not powerful. Maybe it's because the worms are now used to the pesticides, i don't know? Therefore they are not dying and are simply multiplying.
Is there a new pest that you have started sighting in the recent years, but was rare in the past?
Uhhm, i haven't seen any new pests. I only see the difference in the stubbornness of the worms to die.
Is there another pest that seems to have disappeared or that seems to be growing smaller in numbers compared to twenty or thirty years ago?
No, there isn't. If anything, the pests are only increasing in numbers, rather than disappearing.
Which other crops do you grow?
I also grow beans, and maize, and sun flowers and mhunga.
Has your cotton acreage changed in the past years relative that of the other crops?
Yes, the cotton acreage has declined mainly because of the crop's market price. I really loved and enjoyed my cotton, but i am now finding a crop that is selling for a better price on the market. A

crop such as beans. I am planning to grow more beans and i have an area where i want to grow them.
Which pesticides do you use against the pests that you have talked about?
Very good! We use Acetamark against aphids. Then for the worms, the red spider and the American, we can use Dimethoate, or *Gukurahund*i, or karate.
And against moths, what do you use?
We use these same pesticides. Once we spray on the leaves they will not flay and land on these. The moths only affect the leaves; they do not have any effect on the bolls.
From your observations, are there any changes in the amount of pesticides used on your farm, over the years?
In the past, we would only spray every other week, two weeks apart between spray periods, and you would go and scout and see very few pests present. But, the past few seasons, we have been spraying, and then on return the following day we would find pests still present. Not dying, but actually increasing in their numbers.
What do you think is the main cause of all these changes?
It is the power of the pesticides. That is what we have observed. We tried to maintain our measurements that we have always used in the past, but we are observing that either the pesticides have lost power or they still have power, but the pests themselves have developed resistance.
In terms of quantity of the pesticides that you are currently using on your farm, are you using enough?
Well, it may be enough if i use it following the instructions written on the label. I follow the label directions. However, the pesticides are not effective in killing the pests
So, when you realise that the pesticides are not effective in killing the pests, what do you do?
I double the concentration of the pesticides. If the label directions say that i should put only 30 mls in my 15 litre knapsack, i simply double and make it 60mls.
When you double it, do you see any effect?
Were sometimes there may be changes, because the following day when i go and inspect my field i find some dead pests, but still, there will also still be those resistant ones which will still be alive.
Do you use any other methods of pest control?
Before we start spraying, we first inspect the plants, as a family. We kill some worms that we find with our own hands. Afterwards, we then spray the chemicals to kill those that would have hidden
Which weeds are common on your field?
Here the main weeds that we find are *chinzungu, bwerere*, and *chaguduma*.
How do you control these weeds?
I often use both herbicides and manual weeding. When i use herbicides, like last year, i use Paraquat.
Do you get any training on the usage of pesticides?
Yes, from our agricultural extension officers

Thank You for your time

CZ 07
Which are the names of pests which are commonly found on your farm?
There are those that eat the cotton, and others that suck the sap. Since i started growing cotton, if you delaying spraying your cotton, it will be attacked by aphids. When it grows reaching the stage of developing bolls you start to encounter red bollworms and the American bollworm.
Which is the most problematic?
Yes, the red bollworm is the most problematic pest.
Are there any changes that you notice in the amount of these pests?
Well, these have always been there. It’s only when you delay spraying them that you think they are increasing. They need carbaryl to prevent them. So you must not delay. But, in general, these pests

have not increased or decreased. I only notice that if there is a delay on my part i tend to encounter more of the pests.
Are there any new pest sightings?
There is one whose name i do not know. It has spikes, it eats on the cotton plant. Its colour is brown. It was there in the past, but it was not common in the cotton. With this one, even if you put pesticides, it won't die.
Do you have any other concerns with pests on your farm?
Scouting in the field is important. If you see the America bollworm, then be assured there are eggs on the undersides of the leaves.
Which other crops do you grow?
I also grow maize, *mashava*, and *mhunga*.
Have there been any changes in the relative acreage of cotton over the past years?
Well, in my case there are always changes.
Which pesticides do you use on your farm?
I use Fenvalerate, cabryl-85, then Acetamark for aphids.
Are there any changes in the amount of pesticides used?
If you start late you use a lot of pesticides. But if you grow your cotton at the beginning of the season you tend to use less because you will have started before the pests have arrived.
But, i also realise that in the past in this field here i would need only two bottles, but nowadays i need up to 8 bottles. The amount of pesticides that i need now to control pests on the same farm has more than doubled.
So, you are now using more, but do you find that the pesticides are enough?
It's actually not enough because i can not completely destroy the pests.
Do you use any other methods of pest control?
There are other methods, but i always use pesticides. For weeds, i normally weed manually.
Have you ever had training on other methods of pest control?
I do scouting, and if there are no moths i do not spray. The agricultural extension officers are the ones who teach us the techniques of scouting
Thank You for your time

CZ 08
What are the names of all pests found on your farm?
These are several pests that are found, such as some worms that when sprayed on we need to find the type of pesticides that kill them
What are the manes of these worms?
There is one that is called round worm, but the extension officers are the ones who know best.
Which is the most problematic?
I can't remember the real name right now
Have you noticed any changes in pest population since your first involvement in cotton farming?
They are increasing
What might be the cause?
I think its because of resistance that we are giving to these pests, because of the many types of pesticides that we use. You hear one farmer say i use this pesticide and another one saying they use that, and so on. But, we are not able to completely kill them.
Which other crops do you grow?
I grow maize, mhunga and mashava.
Have there been any significant changes in the relative proportions of all the crops which you grow over the past years?
Yes. The land used for cotton is getting smaller, because when we sell our cotton we are not getting good satisfactory returns. In the past if i sold a bale of cotton i would buy a cow, but now i cannot

buy even a bar of soap. I they say i should get 50or 60 dollars for the bale, it means i am getting absolutely nothing for my crop and labour.
Which pesticides do you use?
We normally buy from Agricura, and this company supplies us with a lot of pesticides that we use. Fernkill, usually, is the one that kills most of the worms.
And for the weed, what do you do?
Our disadvantage is that for the weeds we do not use any herbicides, we just weed manually. So, we weed three times in the season.
Are there any changes in pesticide uses?
Yes, there are now many types, we hear there is this type and that type. But, from my experience Fernkill is the only one that is very helpful. The other ones do not help us in any way.
Are these pesticides enough?
Well, we are now growing on a smaller portion. There are some who are now just doing regrows, which need a lot of pesticides. It's because they are attacked by pests early. It's not allowed, but sometimes people are tempted to just steal also.
Have you ever done regrowths yourself, no i haven't, but i am hoping that this season, uhhm .. uhmm, those who are doing regrowths are the ones who are actually having good harvests.
Do you use other methods of pest control?
We use pesticides only. There is no other way of dealing with the pests. But, for the weeds, i sometimes weed manually.
Have you ever received any training on the management of pests?
Yes, the extension officers come and teach us.
Thank You for your time

CZ 09
Can you please name all the pesticides which you come across on your farm?
Mainly the red bollworm is the one that i see the most. Then there are also aphids that i see. These feed on the leaves. The red boll worm is the most problematic pest in cotton
Over the past 35 years, have there been any changes in pest populations?
If we compare pests in the past with pests now there is a difference. I see that the way we live now is making it different. The pesticides that we use now are different. In the past, we would receive two buckets of cabaryl-85, and one bottle of Thiodan. During that time, i think we were getting more pesticides. Nowadays it seems the amount of pesticides are being reduced. They are now being overpowered by the pests. People nowadays are buying extra pesticides on top of what used to be the standard and what they receive from the cotton companies. Its different from in the past when they were just throwing pesticides at us for use. They didn't have much knowledge.
Would you say in the past there were more pests?
No, there is no difference, its only that in the past there were more pesticides made available to the farmers. Nowadays, if you want to get more pesticides the cost of them is rather prohibitively high. So, the pests are now getting their chance.
And the types of pests are there any changes?
I do not see any changes. The same pests that were there in the past are the same that are there now.
Which weeds do you have to deal with in your cotton fields?
We now have certain types weed that we are now finding in our fields. Not knowing whether its the result of crops that we receive that come from other places. There is the weed called chinzungu which we didn't know in the past which is now a problematic pest in the area.
Which other crops do you grow?
I also grow maize and ground nuts
How does the size of cotton acreage compare with the acreage of other crops over the past years?

The acreage of cotton is becoming smaller relative the other crops? It's getting smaller because these days for the farmer to buy inputs, let me give an example, in the past when we used to get financial loans; if the loaners say 4 hectares of cotton, they would give you 8 fifty kg bags of D fertilisers, and 4 bags of top fertilisers. That time the prices were very affordable. Nowadays, you just receive just one or two bags. So, it's now different, and people are now growing on small portions that are in line with the amount of inputs they have.
In the past through the 1980s we were growing cotton on a larger scale. But of late things have changed. This is also due to the rainfall which has also changed. The way it is raining these days is different, it's lower. It's stopping much earlier. It's coming on time, but then it goes much early. That's the problem which i see. Maybe the climate has changed, i wouldn't know. But i see that the main problem is that the rain season is coming to an end early.
Which pesticides do you use?
In the past we used pesticides such as Agrithane; nowadays we use the likes of Fenvalerate.
Against which pests do you use Fenvalerate?
There is 85 and Fenvalerate. They work in the same way only that 85 is used first before there is more rain. When the rains have picked, we start using Fenvalerate because when it rains Fenvalerate cannot be washed by the rains. That's the difference.
Which other pesticides do you use?
There are a lot of them.
Which ones do you recall?
There is Fenvalerate, and then there is Thiodan, then 85, and the other ones i cannot recall right now.
Which ones do you use to kill weeds?
Honestly speaking, i have never used any herbicides. I only weed manually. I do not have much knowledge about the herbicides. I only hear other people talk about them. But, i would also want to use them, because when things start, it's just one person who starts with it and then the rest of the people also join until everyone has knowledge.
Since you started cotton growing, would you say there have been any changes in the amount of pesticides that you use per hectare?
During the old days we used a lot of pesticides. Nowadays we notice that the amount of pesticides used is getting lower.
Why?
Because the price is getting higher and things are getting more expensive.
Are the amounts of pesticides that you are currently using enough to control pests on your farm?
Well, the pesticides are enough, if you have your money. Because there can never be a shortage of pesticides. There is plenty of pesticides. The only problem that is see with regards to the pesticides that we buy is that when they expire they never tell us that these pesticides have expired. They just market knowing that their pesticides have expired. I only know that it's expired when i spray. Even when i try to increase the concentration of the pesticides, they don't work. It's only after close examination that i realise that we are buying pesticides that are expired. For example, that is the case with those companies from which we get loans. They never explain to us that the pesticides are expired. When we use the pesticides the worms will damage our crops because the pesticides will have no effect on them. There, that is where i have seen that there is a problem. They do not tell us that their pesticides are expired.
So, you also do contract farming?
Yes, i sometimes do.
Have you attended training on integrated pest management?
I have attended once at *Chimhanda*, but i did not complete the training. So i didn't get a certificate. But our agricultural extension officers always educate us.
Do you use other methods of pest control?

I only use pesticides to kill insect pests. There is no other way. But, when it comes to weed, i always use a hoe to weed manually.
How do you know that it's now time to spray, Is it by following the calendar, or you use other methods?
I scout for insects. When there are no insects i do not spray.

Thank You

CZ 10
Since when have you been involved in cotton farming?
We started just after independence. We started growing cotton in 1982. When we started i harvested 16 bales, and then i became the chairman of *Kuwirirana* cotton farming group.

What is the size of your cotton field?
In the beginning we had 2 acres. We were getting inputs on loan. We would be loaned equipment and chemicals. They would give us 4 containers of chemicals.

Which type of agro-chemicals were you receiving?
We were receiving different types, including Rogor, carbaryl 85, Thiodan and *gukurahundi.*

Has your acreage ever changed from the initial 2 acres?
We have been surpassing that, reaching 5 to 6 acres. We were now enjoying it, and the family has been growing; we needed money to pay for school fees. We used to grow different types of cotton varieties on demo-plots, including FQ, SZ, and others.

6:46,6 - 9:41,2
What are the names of cotton pests that are commonly found on your farm?
We first encounter Aphids. When your cotton has grown 15 centimetres, you first spray with rogor.

Against which pests will you be spraying it?
Against aphids

Do you spray with rogor only because the plant has reached 15 cm, or are there other factors that you consider?
We scout for aphids first. We also used the same scouting technique to identify red spider, and against red spider we used *mospran,* which was very effective against red spider.

Other than Aphids and the Red Spider, which other pests are found?
There is the Red Boll worm, once the cotton has developed bolls. We scout for it. Red boll worm comes first, followed by the American boll worm. If you scout and see there are boll worms, you start spraying the affected area, and then you spray the rest of the field as preventive.

Are there still other pests?
There are other ground pests that are killed using a pesticide that is applied on the cotton seed.

What are the names of these pests found in the ground?
These include certain worms and millipedes.

9:41,2 - 11:16,6
With which of these pests would you say you have had the most problems over the past 30 years?

With the red boll worm we have the advantage that it often comes out of the boll, and when it does so that is when it encounters the pesticides outside. The American boll worm, on the other hand, only comes out of the boll after it has finished eating the whole boll and has laid its eggs inside the boll.

So, is that the reason why farmers are encouraged to?
To slash and burn their cotton stalks. Precisely!
And, when you are spraying your cotton, it is encouraged also to spray on the edges of the field as a preventive measure against the movement o moths.

Oh, so moths are another of the pests to add to the ones which you mentioned earlier on?
Yes, these moths lay their eggs on the cotton bolls which later turn into worms.

11:16,6 - 12:19,6
From around 1982 when you started growing cotton, have you been noting any changes in the amount of pests found on your far?
Aphids are the ones which seem to have been growing in terms of their amount. For your crop to be a good crop, you must first win the initial battle against aphids. However, we do not always get the pesticides against these aphids in time. If you manage to get your pesticides against aphids in time, you know that you are growing your cotton very well, because carbaryl 85 will make your cotton look healthy with very good leaves, but `you will have sprayed for aphids.

12:19,6 - 12:58,6
Have there been any changes in the types of pests on your farm since you started growing cotton back in the early 1980s?
There are beetles, called ladybird that come at the end when you are about to harvest. They fly.

Were these not found during the early days in the 1980s?
No, they were not there.

12:58,6 - 13:46,4
Which other crops have you been growing along with cotton over the past 30 or so years?
We also grow maize for food. We can not only grow cotton while we have nowhere to get food, that is why the acreage of cotton sometimes tend to get reduced when we grow more maize for food.

13:46,4 - 14:39,8
You said earlier on, before we started recording, that your cotton acreage has dropped significantly and you no longer prioritise cotton as much as you used to in the past. Which crops are you now growing in the fields that were once dedicated to cotton?
We now grow maize, sorghum, and rapoko.

What made you to scale down on your cotton production?
We scaled down on cotton because of the market price; cotton is a very labour intensive crop and the market price has been going down. So we see it better to concentrate on food crops.

14:39,8 - 16:01,0
What are the names of pesticides that you have used on your farm in the past 30 years?
The first pesticide that we use is rogor, followed by carbaryl 85. After carbaryl 85 when you start noticing worms you introduce Thiodan, then you use *gukurahundi* at the end.

Which pesticide is *gukurahundi*?

It’s a name used for pesticides such as Fenkill, and so on. These days the names are always changing. 20 ECC used to be

16:01,0 - 19:13,2
Has the amount of pesticides that you use on your farm changed over the past 30 years? Why?
There is a change. In the past, when we were being loaned, we would get enough pesticides for the whole acreage, if you decided to buy on your own that is when you would notice that your crops would be attacked by pests. That is why we would loan. We would get the whole kit. If, for example you got a loan for two hectares, per hectare they would give you two bottles of Gukurahundi' Rogor two, and two packs of Carbaryl 85.

And these recent years, is that the same amount that you are still using per hectare?
These days the quantities have declined. They no longer give a full set.

Now that the quantities are lower, would you say that when you spray your fields you are having enough pesticides against the pests which you encounter?
Yes, its enough because the bottles of pesticides these days are a bit bigger; but the pesticides are not as strong as the ones which we used in the past. In the past the pesticides were much stronger. Even Thiodan which is now called Thiamex; there is no longer the Thiodan that used to be there in the past. That one was quite strong; if you used it there was never going to be a single worm found on your cotton.

So, are you suggesting that the pesticides used these days are no longer as strong?
Definitely, even a lot of other farmers agree that these days the pesticide manufacturers are 'limiting' the strength of the pesticides. Even the moths no longer have 'excuse' with the pesticides. We may wrongly say that they have now become resistant, and yet the pesticides are the ones which are now much weaker.
38:34,5 - 39:58,8
You said, earlier when we had not started recording that you use herbicides to control weeds in both your cotton and your maize. Which herbicides do you use?
Yes, i use atrazine.
43:15,0 - 45:13,2
Other than using chemical pesticides, are there other methods of pest control that you use?
We scout. Scouting is the best method of protecting your crops. There are times when you just waste pesticides when you are spraying even when there are no pests. Scouting is growing cotton! But, at the beginning when your crop is just about fifteen centimetres you must spray your cotton, even when you have not scouted or seen any pests in order to protect against aphids. When it has reached about h40 centimetres you spray with carbaryl 87 so that your cotton can have good leaves.

45:12,0 - 49:14,2
Have you ever attended a course on integrated pest management?
I have never attended any training, but my knowledge has been passed on to me by our agricultural extension officers. The extension officers have been our teachers; always. When i was the chairman of our cotton farming group, i would go to Rushinga and the agricultural extension officers would teach all chairmen who would have gathered. When i returned home i would then call for a meeting with all the members of my group and then i would go into the field of each and every one of them and demonstrate how to scout for pests and teach them all that i would have learned from the extension officers. I used to produce a report about what i observed in every farmer's field, and what i would have advised them.

Thank You

CZ 11

Which pests do you encounter on your farm?

We first encounter leaf eaters, and then we find aphids

Which leaf eaters are these?

These include grasshoppers, the gaggers, and the spodoptera. Then we also encounter aphids, these aphids can be put in the same group as termites that stay underneath the plant in the ground. Then there are some spines, Redboll worms, the American which we call the heliothis. These are the main pests that are found in cotton. But, this season there has been spotted a new pest which we have dubbed *madzibaba*. We have never had serious pests with such moth in the past. In the past we could only spot it after the cotton was already white and due for harvest. That's when we would spot it on cotton. It's brownish, it does not fly away if you approach it, and you can easily catch it. It's like an egg, and it just develops like that.

Why do you call in madzibaba?

Because it is white in colour It looks like an envelope.

Its main effect is that it affects the colour of the cotton. Cotton is white or goldish, but when this pest lands on the cotton it shed the colour leaving it brownish in colour.

Then, among the leaf eaters, there is another one called anomis, this is one among those that feed on the leaves.

There are also good insects that are our ''helpers'' that we see eating some of the insects. For example, there are *twukamba* which feed on eggs (of the worms), and there are also syrphids which also feed on eggs and smaller moths.. But, the problem with the syrphids is that when it feed it feeds its excreta resemble that of the spine. Therefore, farmers may not recognise the difference and start spraying thinking that there is spine.

With which pest have you had most problems over the years?

Yes, as far as I have observed, the American is the most dangerous and not the red bollworm as many people often believe. The American can destroy up to 60 bolls in one day because it just feeds on a bit of the flowers, and then without finishing that particular flower, it abandons it and moves on to the next one, but damage will have already been affected. It can potentially destroy 30 to 60 flowers in a single day.

Over the years, have you noticed any changes in the population of pests you encounter?

Yes, in the past they were fewer, and now they seem to have slightly increased in their population. In the past, the American was green, and now it is coming in two colours, a mixture of green and brown colours. But, this stripped one is still an American, its feeding mechanism is the same. But, it is not difficult to control because this American does not get inside the boll. So, as soon as you finish spraying, if it gets exposed to the pesticides it can easily die.

And in terms of pest types, are there any changes?

No, all the pest that were there in the past are the ones which we see now. However, the manner in which we are controlling them is the one that is disturbing. You see, what happens is, some pests are very fast to get destroyed. That is why you see that others are not being very clear when they talk. Farmers are not getting the pesticides on time, they get them late that is why there end up appearing a lot of pesticides in the fields. The pesticides are not available in time. The issue is, farmers do not have the money to buy pesticides, and they wait for the pesticides which they get on credit from the cotton companies. But, the distribution of these pesticides is not efficient, they arrive late. Therefore pests end up multiplying in the fields. In the past, when farmers signed for input loans with one company which was operating in the area then, they would receive all their inputs at once, before they started planting. When farmers received their pesticides before they even planted, they would control the pests once they appeared, and this managed pests to low levels. This time, farmers receive pesticides when the pests are already appearing and normally they would have already lost several bolls to the pests.

So, are you, effectively, saying that the problem is the management of the pests, and not the pests themselves?
Yes, that is the problem.
Are there any other concerns with regards to pests on your farm?
What happens is that if pesticides are used correctly, they control pests effectively. The problem is that we, the farmers do not have enough knowledge and have not received enough education about proper usage of pesticides. Personally, i lay my blame on our agricultural extension officers. There is not enough knowledge and education in those people.
I see!
They do not have enough knowledge about this crop, cotton! I wish they had also gone to Kadoma cotton research institute where i went to do my cotton training. I have often gone around my ward, ward 13, just to assist farmers and impart them with knowledge about this crop and the proper usage of pesticides. There are still people who are using wrong pesticide to water proportion mixtures. They have the belief that if they put more pesticides and make a stronger concentrate that will easily kill the pests. So, there is really need for educating all the farmers.
Do you also grow other crops apart from cotton?
In fact, there is also the issue that farmers ignore extension officers' advice. The extension worker may call for a meeting with farmers and educate them on certain issues, but then the farmers always have the attitude that they know better than the extension workers because they have many years of farming experience while the extension officer is often young and inexperienced in as far as farming is concerned. But, the farmers tend to overlook the fact that the extension workers will have come from agricultural colleges. Others say that they have been growing cotton for a very long time, so they know everything that needs to be known about it, yet they do not know that there are many changes that are happening due to changes in climate. You see? So, the agricultural extension officers are to blame and yet they are also sometimes not to blame because some farmers say they have been growing cotton for a very long time.
Okay, i see. Which other crops do you grow, in addition to cotton?
I also grow maize, *mhunga, mashava*, and sometimes i grow *mhunga* enough to supply the whole of ward 13. There was one season in which i harvested 60 bags of mhunga, and i gave to people in need in the area.
Is mashava in Shona the same as millet in English?
No, *mhunga* is millet. Mashava is sorghum.
Okay.
Maize! Each year i harvest 2 and half tonnes. I was lucky at one point that a local ngo working in the area wanted to build me a small domestic sile for the storage of my harvests, but the elders of the village became jealous; you know, if the king does not have it, you can't have it. So the money was diverted and the domestic sile was built at the chief's homestead instead. So, at the moment i am storing my harvest in the house. Despite the fact that my silo was diverted, that does not deter me from growing and producing. Each year i do dry planting of maize and harvest 2 and half bales. I never under achieve. I do not know what hunger is.
Do you use any pesticides on the other crops which you also grow?
For spraying?
Yes
No, i do not use any pesticides on other crops, except only in cotton.
Are there any changes in the acreage of cotton relative the acreage of the other crops?
Well, according with the tonnage that i harvest in cotton or maize, if i realise that it's better to increase my cotton acreage because i have a certain target, i just increase the cotton acreage. But the maize acreage remains the same because at times we harvest a lot of maize, much more than we can consume such that when we harvest we will still have plenty of maize from the previous season's harvest.
Which pesticides have you used since you started growing cotton?

Usually each season i first use Acetamark against aphids. After Acetamark, i use Carbaryl 85 so that i can boost my cotton and that its leaves may broaden so that it can photosynthesize at maximum potential. Then, I then use Gukurahundi, which is Fernkill and Fenvalerate. Sometimes Fernkill and Fenvalerate are ''used'' to our fields here in *Rushinga*. Sometimes you hear a person saying these pesticides are no longer killing pests. It's because there is no (longer) rotation with acaricides. So, there is need for the rotation and the introduction of other pesticides such as monochrotophos so that we may stop the use of Fenvalerate. The red bollworm and the American are now resisting Fenvalerate because all the years these are the same pesticides that are used against these worms.
So, is there no rotation?
Yes, there is no rotation of these two pesticides. There is always Fenvalerate and Fernkill. They are both Gukurahundi, in the same group as monochrotophos. But, Monochrotophos is good, maybe they are afraid because of that purple label, but farmers are ''now educated'' they know that those pesticides kill. They just need a little education, but i can say that most farmers know that there is need for protective clothes. Monochrotophos has a purple label, if it is introduced in the field it can cause a ''reaction'' of ''controlling'' the red boll worm and the American so that they do not just get used to Fernkill and Fenvalerate.
So, are these the main pesticides which you use?
There are also, among the aphids that i already talked about, there is also that other pest, this one that is controlled by acaricides; there are acaricides that control the red pest aphid, what is it called by the way?
Is it the beetle?
No, it's not the beetle. Okay, lets continue, i will remember it as we talk.
Alright, just say it when it comes to your mind.
Are there any changes, over the years, in the amounts of pesticides which you use on your farm?
Well, that now depends on knowledge. I can grow a small area and use less pesticides, depending on whether scouting will be happening or not. Scouting is the thing that is ''very very important''. You may walk into the field and see signs of feeding by pests on your crops. This does not necessarily mean that you should then get in with the pesticides and spray. Rather, you should then do the scouting. Sometimes scouting is good. In the past we did zig-zag, or steady traverse. This is the one that is currently used. How this is done is, you step left, then right, right. By so doing, you can quickly tell the extent of the presence of pests and then plan to return and spray. If you just decide to enter and spray without doing any scouting, there might only be one plant that would have been attacked by the pests while the rest of the field not scouted would have not been affected.
Are the pesticides which you currently use on your farm enough to control pests?
Eh, yea. All the pesticides that we have talked about, especially the Gukurahundi which control the worms are in the same group; they work, they are effective. There are no pesticides that are ineffective. What is needed is the ''reaction'' of the pesticides when mixed with water in the knapsack. That is the moment when the 'reaction' of killing changes. If you pour cup number 22 or cup number 30 in 15 litres of water, by the time the sound of the bubbles is heard, its indicative that the pesticide is effective and can kill. But, when we, farmers, add cup number 60 instead of cup number 30, the pesticide will be rendered ineffective. Let me give you a practical example. Have you ever seen, say, if we take a red bollworm and throw it in a container of Gukurahundi, say Fernkill? It will just move and go. Nothing will happen because there will not be any reaction that would have happened. But, if you add water, it will die.
Do you use any other forms of pest control other than the usage of chemical pesticides?
Yes. I, may do what is called ''acaricides'' in English whereby this week i may do scouting at the very beginning before i start using pesticides, i may confuse and control pests by using hot chillies. Even when the cotton has developed bolls, i may confuse the worms so that they do not get used and resistant to pesticides by using hot chillies. I just grind a lot of hot chillies and put in the knapsack and spray with it. When the pests get exposed to the hot chilly mixture they will not move and

simply stay in one place. That is the method that i often use by using our own indigenous way of pest control.
How do you use the acaricides? (Question asked by research assistant who interjected)
The pesticides?
Yes
I can use acaricides, remember i was saying there is red spider mite; oh, yes i have now remembered that pest which i was failing to recall earlier on.
Oh, okay, the red spider mite! Yes, the red spider mite. Red spider mite, also known as *kafuwe* in Shona, is often found invading the cotton fields. These are aphids which, once they invade a cotton field, they ''bomb'' till the cotton wilts and eventually just dries. These suck a lot of plant sap. So, against the red spider mite i use ''what is called acaricides''. It is mainly killed by using rogor. I first do spot spray, if i see that; okay, it's here and there and there and there. I leave markers where i spot them, and return with the acaricides and do ''spot spray''. If i spot it at more than five points in the field, i then do ''blanket spray'' whereby i just spray the whole field. So, for the control of acaricides, here in Rushinga we may be using Fernkill, while in another part of the country, say Gokwe, they may be using Fenvalerate Then, in Chiredzi they may use monochrotophos. Next year, in Chiredzi they may receive Fenvalerate, and then monochrotophos should be sent to Rushinga and Fernkill to Gokwe.
Okay!
That is what is called acaricides!
Oh yes; rotation!
So that by so doing this rotation the pests may not get resistant to the pesticides. For example, here its year after year Fernkill, Fernkill, Fernkill.
Okay, so here there is no defined rotation that is happening, is that what you are saying?
Yes, there is no proper acaricides here!
But, what is causing that? That there is no rotation? Is it because you just buy what is available?
What is causing this is that these cotton companies are going to request that in Rushinga the farmers are mainly interested in gukurahundi pesticides which are Fernkill and Fenvalerate. They do not have knowledge that there must be acaricides (rotation).
Have you ever received any training on integrated pest management?
No, i have never done a course on the management of pests alone. However, when i did my cotton growing course in Kadoma, the course covered issues about pest management. But, i have the desire to do such a course, if, in my ward there were to be chosen an individual to do such a course.
What was the name of the course which you attended in Kadoma? (Research assistant)
It was called Cotton Management and Selling.

Thank You for your Time

CZ 12
Since when have you been involved in cotton farming?
I have been growing cotton since 1994 when i completed my secondary education; but i actually started to be actively involved in activities such as spraying since 1989 when i was only fifteen years old.
What is the size of your farm?
The size of my farm, at the moment is if 5 hectares
On these 5 hectares, do you mix cotton with other crops?
Yes, i mix with other crops.
So, what percentage is taken up by cotton, on average?
Two hectares
And which other crops do you grow on the remaining 3 hectares?
I grow ground nuts on 1 hectare and maize on the remaining 2 hectares.

Do you use any chemical pesticides in growing maize and ground nuts?
Yes, i use herbicides in maize. I use Roundup and Gramaxon.
Alright, now, let's talk about cotton. Which pests do you encounter in your cotton farming?
The first thing when growing cotton, the pests that we encounter at the beginning of the season that attack our crop are aphids. After aphids, there come jassids. These are small flying insects that suck the sap out of the leaves. This results in leaves wilting and drying u. Then, again in cotton there is the red spider which is also found feeding on the plant.
So, red spider comes after these two?
Yes, the way it enters the cotton field is usually through being brought from the tomato fields when its present there. It sticks on the clothes and when you enter the cotton field it then moves onto the cotton plant. Where it is present you find the portion of the crop that is affected appears to have leaves that are burnt in appearance. The leaves will just be falling off, and the cotton will not grow. Then there is a worm that we call the American bollworm. This appears when the cotton is at the flowering stage. This worm attacks the flower, it feeds on the flowers. It's inside at the centre of the flower. Then when the cotton has developed bolls, there is the red bollworm.
So, just clarify this to me. The American bollworm appears when...
It appears when the plant has flowered, towards the stage of developing the bolls. That's when it appears and destroys.
And the red bollworm comes afterwards?
Yes, the red bollworm follows to enter inside the bolls and feed from within the bolls.
Oh, okay, that's the difference. I see.
Yes. These are the main worms found here in Rushinga.
Of these pests, with which one have you had the most problems over the past years since your initial involvement in cotton farming?
The most problematic are the aphids. Aphids interfere with the growth of cotton. When they are present, the cotton will not grow well. It will not grow at all. The leaves will appear dirty as if there is molasses. Aphids are mainly promoted by lack of rainfall. If there is a lengthy dry spell, for example three weeks, there will appear aphids. But, there are certain cotton varieties that are less susceptible to aphid attack because these have fewer leaves, for example the seed variety called Alba FQ 904. This variety has few leaves, so plant cannot harbour a lot of aphids.
Okay. I see. Yes, because the leaves are fewer. So, are they trying to come up with varieties with fewer leaves?
From when you were first involved in cotton farming till now, are there any changes in the pest population in cotton?
Yes. There are a lot of pests population.
Compared with the population in the past, are there any changes?
Yes, there are changes. The pest population has significantly increased because nowadays people are increasingly lacking money for buying seeds and are therefore resorting to ''regrowths'' of the old plants from the previous season. We are supposed to but slashing and burning these cotton stalks which others are now growing as their new plants in a new season. Others only destroy their crops too late after the deadline for destroying and burning the cotton stalks. Therefore, these pests are harboured for a long time and they do not completely die. Therefore they increase their population. When farmers favour regrowths, there is no longer crop rotation in the fields as they continue with the same crops in the same fields. It's now different from what the situation was like in the past when the agricultural extension officers would do inspections, encourage farmers to destroy cotton stalks and even fine those who flouted the regulations.
Still on pests, are there any changes that have happened in terms of pest types over the years?
Yes, there is a certain type of worm. I do not recall its name. It's rare, but it somehow resembles the American bollworm in some way. It attacks crops by destroying the shooting stem of the plant. When this worm has attacked, you just notice the plant by its wilting appearance. When you dig under the plant you also notice that it also attacks the root core of the plant as well. But, this worm

is rare, but it seems to be multiplying as a result of the regrowths which are increasingly becoming popular.
So, you often come across this worm?
Well, it's rare. What is often seen is its destructive effects. Only if you are lucky, that is when you can actually come across it. Since i started farming i have only come across it just once. I normally always see where it would have gone past by the destruction that it leaves. It is increasing its effect, and possibly population, as a result of the regrowers.
Which weeds are found on your farm?
The weeds that i come across are broad-leaves, for example *chimunzwa/chifeso,* and then there is *chaguduma, mhande* and bwerere. Bwerere has lateral roots which just spread on the ground such that when it rains after weeding it without shaking off the soil it will immediately grow again.
Do you use any herbicides against these?
Yes, i use Gramaxon. I may use in combination with paraquat. This, i do during planting because if i use Paraquat after planting i may destroy all the plants. One solution is to modify the nozzle of the sprayer by putting a small funnel made using the opening of a plastic drink bottle, and then spray pointing to the ground, not reaching the plant.
Okay. I see. Now, tell me. You said you also grow maize and groundnuts. Since you started growing cotton, have there been any changes in the acreage of cotton relative the acreage of the other crops?
My cotton acreage sometimes goes up, so i often rent land from others and grow up to 5 hectares of cotton in some seasons. I usually exceed 2 hectares when i have enough money to buy inputs. But, the other reason why i end up renting land from others is that i often do demo plots, usually with 10 to 12 different varieties. So, i then take out of these varieties and demonstrate to people during field days which varieties are most suited for our area of Rushinga. Then we explain to fellow farmers the advantages and disadvantages of different varieties. This is in the same line with the variety which i talked about earlier, the FQ904, which has fewer leaves.
This variety with fewer leaves; don't the fewer leaves affect the amount of chlorophyll for the plant and thus the size and amount of bolls per plant?
No. They actually develop good big balls. In addition, the quality of the cotton is good such that it fetches a higher price on the market per kilo.
But, then. I think this is a bit pricey to buy as seed?
Yes, it is a bit expensive. They differentiate the pricing. I even grew once seed cotton as foundation.
Of the cotton which you have grown in your demo-plots, are there varieties which you would strongly recommend to not be grown here in Rushinga?
Yes, particularly SZ94. This one is not good. It just develops a lot of attractive looking big leaves that the plants look like shrubs, but when it comes to boll production it develops very few bolls.
Okay, so when it develops many broad leaves it also implies more pesticides will have to be used, right?
Right, and the aphids themselves become fulltime resident because there will be a lot of leaves.
Are there many who grow it?
Yes, particularly in the past, many people used to grow it, but now the agricultural extension officers are discouraging farmers from growing it.
Just tell me, do those farmers who get their inputs on credit have any choice as far as choosing which seed to grow is concerned?
What happens is, it depends on when you want to borrow they ask you how much you produced the previous season. If you produced 1000kg you belong to the Gold Club. Those in the gold club are respected farmers, with their own files at the cotton company. They are different from those who produce just about 200kg. Those would not ask for the most expensive seed. But, those in the Gold club have the privilege of receiving the most expensive seed varieties. These are given demo plots which will be used for field days. Those who receive the seed, particularly to grow cotton seed, are not permitted to grow any other variety because this seed is not allowed to mix with the other

varieties. They extract the seed at the ginnery, then that seed variety will be sent back into production.
Which pesticides do you use against pests on your farm?
When we start spraying at the beginning of the season we use Oncol, or Marshall, or Dimethoate. These are the pesticides that we use against aphids and jassids which suck the chlorophyll out of the leaves. Thereafter, we use Carbaryl 85. This pesticide provides 'feeding' to the cotton. The leaves remain green, and the crop will be healthy. If the leaves are not healthy, then we do not have cotton. A tree without good leaves is not a tree. So, we use Carbaryl, it gives feeding. In addition, Carbaryl is used to control the worm which i talked about earlier on, the American bollworm. Here in Rushinga, the regulation is that we do not use the gukurahundi pesticides, which are Fenvalerate and Fernkill, before 1 February.
Oh, i see. So, there are dates when these can be used?
Yes, the reason being that if you start using Fernkill or Fenvalerate while your crop is still 15cm, 20cm, or 30cm it will not grow any taller than that. These gukurahundi pesticides are there to be used only for a short period. If they are used from the first of February, by the 10th of March, the bolls of our cotton will be opening. So, if you start to use it in November, October, November, December, January, February, March, April, using gukurahundi all these months, cotton will not grow. In addition, the worm will become very resistant. If you do not have Fernkill or Fenvalerate, as determined by the size of your cotton, you use Carbaryl-85. You will use measurements such as Number 30 if it has grown too tall. Say the 1st of February comes but the companies from which loans are applied for have not delivered, and you have Carbaryly-85, you take cup number 30 and mix 10 cups in 50 litres of water. This will work in the place for Fernkill or Fenvalerate.
Tell me, when gukurahundi is used, is there a rotation of these in a systematic manner?
No, we just use them both depending on which one they provide us with. There is Tamolin, which is also there, then Fenvalerate and Fernkill. Then, i had forgotten when i gave the names of the pesticides used against aphids. There is Acetamark, a sachet that is approximately 50 grams. That sachet will be enough to use on a 1 hectare plot till harvest. It has a number 3 cup, the smallest. You take this and mix in 500mls of water and shake and then use cup number 30 to measure from this and mix with 15 litres of water in the knap sack and get in the field to spray.
So, the gukurahundi, you say, is used against the American bollworm, right?
Yes, for the American bollworm. And also, for the red bollworm and the American bollworm. It can work. What is not allowed when using pesticides, is to mix powder and liquid pesticides. For example, say today i want to spray and then i take Carbaryl 85, Oncol, and Fenvalerate and mix in the same knapsack. It's not recommended because you will notice the plants developing black spots on the leaves. It’s not recommended. Let the powder be used on it’s on, and the liquid on its own.
So, explain this to me. Let's say this week i use Fenvalerate and the use another type next week, is it not discouraged?
It's possible to do that, but most farmers do not know much about how to grow cotton, otherwise if they do that their debts will continue spiralling. In cotton farming, there is what we call scouting. By so doing, a farmer will be checking for pests in the field, time and again the farmer moves on the edges, in the middle and again on the other edges, and see how many plants are affected by aphids. Put a marker there where there are aphids. Move again towards the centre, and see if there is something, in a squared area. Then if there are pests in just one squared area and the rest of the field is not affected, go get the knapsack and spray only that squared area which is affected by the pests. The problem with those farmers who do not know the technique of scouting is that once they see pests on one or two plants they spray the whole field. Wasting pesticides. It is important to know what is scouting. Just see where you have seen the American bollworm and spray only that area where you have observed the bollworm. If you do that, as a farmer, your pesticides loan will not go up. Loans go up due to lack of knowledge. That is when you find farmers spending a long time in the field spraying while there is nothing in the field. You hear farmers saying gentle this week i did not spray, so next week i want to spray. That is wrong. Farmers must see if there are pests in the

field and ask themselves if the amounts of pests are in tandem with the amount of pesticides which they use.
Can you say that over the past 20 or so years that you have been involved in cotton farming there have been any changes in the amount of pesticides used to control pests on your farm?
No, it's just the same. It's just that changes in pesticide names have changed. In the past, the pesticide which i referred to as Oncol, used to be Rogor. Then it was renamed Dimethoate, Thereafter Oncol, Marshall, and then they gave us that powdered one which i talked about Acetamark.
So, is it the same chemical called by different brand names?
Yes. It is the same. There is the one which we call Acetamark. This is meant for aphids.
So, you are saying what has changed are the names?
Yes, the amount
Yes, the amount of pesticides used has not changed. Because, the dimethoate which we are talking about, we use number 30, the Oncol, we use the same number 30 and then the powdered one, we just use number 30.
So, when other farmers say that the pesticides no longer have power, what do you think they really mean?
Well, it's nothing but ignorance. Not knowing the right pesticide to use. You see a farmer getting in the field to spray dimethoate because they may have spotted American bollworms. When they see that the worms are still there after a couple of days, they take the same Dimethoate and Fenvalerate and mix, to make a cocktail, with the belief that if they mix the pesticide will become stronger and kill the worms. They will be doing it wrongly.
So, you say you use cup number 30, do you think this is effective, or you wish there was a bigger cup?
No. This is good. This is 100 percent correct. Because, when i went to study the agronomy of cotton and when i went to do scouting for two years, that is the education that i got. If there are pests in the field, the cup to use is the recommended cup.
But, do you think that what is currently recommended is enough?
Yes, it is enough. It's similar to overdosing a goat that you want to treat for worms. If you overdose what do you expect from it?
I ask because when i speak with other farmers they tell me that when they see that the pests are not dying they increase the concentration of the chemical in the mix.
That does not work. They will actually be making mistakes.
Have you ever received education on how to protect your health from exposures to pesticides?
Yes. When i did my training we were taught that the pesticides are dangerous and they can kill. We were taught to always wear protective clothing and when handling pesticides. We were also taught how to safely store pesticides out of reach of children.

Thank You very much for your participation

CZ 13
Do you weed these crops manually or you use herbicides?
I use herbicides, but i started using herbicides only recently. I had first to consult with the agricultural extension officers, and they told me that it was perfectly alright to use the herbicides instead of manually weeding.
Which herbicide do you use?
I use Atrazine.
Which pesticides have you used in cotton to date?
I have used gukurahundi against the worms, then for aphids i have used Rogor, then carbaryl-85 and Thiodan.
Which pests do you come across on your farm?

There are aphids, and then there is the red boll worm and the American bollworm?
Which among these is the most problematic?
I can tell you that cotton is a crop that does not tolerate being affected by any pests, particularly aphids and that worm, the American bollworm. The effect of the aphids is that the cotton will appear as if its wilting, and this is so unpleasant, and then the American bollworm gets inside the boll, and when it does so it eats everything inside the boll.
Does cotton also have diseases which you have to deal with?
The aphids that i have talked about are the ones that result in the disease for the plant.
Alright, i see. Since you started growing cotton, have you observed any changes as far as pests populations are concerned on your farm?
The changes that i have seen is that even if you are spraying your crop very well, if there is an adjacent farmer who is not spraying his crop well, worms from his farm may migrate to your own crop. But if you are both spraying very well, the worms will be controlled.
That was in terms of population; how about in terms of pest types, are there any changes?
We have observed that sometimes the armyworm may attack our crop, even though it is most common in maize. If spotted in cotton we normally spray the field right round.
Which other crops do you grow?
I also grow maize, ground nuts and *mashava* also known as *mapfunde*.
Has the acreage of cotton changed relative to that of the other crops?
Since i started my acreage was high, until recently when i down-scaled.
Which pesticides did you say you use?
I use gukurahundi, 85, and Rogor, and Thiodan.
Is there another type that was used in the past, put is no longer being found?
Yes, there was Karate. This one was used as Thiodan. Now, it was banned because if it was used in excess amounts it had the effect of 'burning' the cotton. Additionally, it was examined and it was noted that it was not good for the farmers. Some farmers used it properly, but there are also those who used it improperly.
Has there been a change in the amount of pesticides that you use on your farm per unit area?
In the past we used to use a significantly higher amount of pesticides as compared to nowadays.
Why has the amount dropped?
It's mainly because in the past there were fewer cotton farmers, so we could get as much pesticides as we wanted from the cotton companies with whom we had contracts. Nowadays there are now too many of us. We are now being rationed when it comes to receiving pesticides being told that we do not have to always just spray anyhow, we must first survey the field and only spray when pests are spotted.
Are the pesticides you are receiving enough to control pests on your farm?
It's not enough because the strength of these pesticides is limited.
Do you use other methods of pest control other than just pesticides?
No. For insects i use only pesticides. For weeds i use herbicides as well as weeding manually.
How do you know that there are pests? Do you use a time table for spraying?
I first inspect my cotton, the leaves and the bolls to see if there are any pests feeding on my crop, or boring the leaves. Afterwards, i inspect the bolls to see if there are no worms that have bored them. And then i move from corner to corner of the field walking to see. If the field is alright, there is no need for spraying. But if there is evidence of pests attacking my crop i get in and spray. if they still continue eating, i also continue spraying until those worms are all finished.
Do you get education about safe usage of pesticides?
Yes, we have always been getting education from the chairperson of our cotton farming group. Then our agricultural extension officers teach us about the dangers of pesticides, and that they kill; that all containers must be burned, they must not be thrown into the toilet because they may destroy the beneficial worms that live in the toilet.
Thank You for your participation

CZ 14

When was the last season you grew cotton?

I want to go and burn the ratoon crops, just today or tomorrow. The ratoon crops have a problem with the American bollworm.

Are they still in the field?

No, they have been slashed; what's left is to burn them. But, with that, the side effect is that if you harvest ratoon crop it will be a problem on the international market. So, when i worked with field workers i always encouraged farmers to destock it and burn by the 31st of September. But, with the dollarization, if you go down to *Mzarabani or Mkumbura*, ratoon crops are now shrubs, almost trees. Ratoon crops produce more cotton than new plants, but with the problem of the American bollworm, at the international market the cotton will be condemned. That's a key point that should be addressed, but it's not possible to address this as long as the price for cotton is not raised. Farmers cannot afford to go and buy seeds. They would rather go for the ratoon crop. The ratoon crop actually leaves the farmer with more money.

Controlling the American bollworm is very difficult.

Which pesticide is best suited for its control.

It does not have a chemical agent to control it. Unless they discover a chemical.

But, which chemicals are farmers using?

Those guys are not interested. If i sell you my cotton and I'm done with you and i get my cash, and the business is done. When you take that cotton to make lint it will not be having any lint strand, because the cotton with lint strand should be long. But, after attack by the American bollworm, the lint will be chipped, such that you will have to join it for it to reach a certain length. It's not very good for the textile manufacture.

So, you have talked about the American bollworm, which other pests are found here in Rushinga?

Red bollworm, jassids, uhmm, American bollworm, and aphids. The effect of aphids is that the cotton will be discoloured and not clear white. At the same time, if the aphids suck a lot of sap, the strand length will be affected. Therefore, when you spray the aphids with Carbaryl that will help control it.

Jassids are grasshoppers. These eat the leaves. The leaf has chlorophyll, chlorophyll has green pigment, green pigment has photosynthesis, photosynthesis has starch, and starch will produce the strand. So, if the leaf is perforated, it means that the production of starch is affected and that affects everything, and the boll will become stunted.

Which pest is the deadliest?

The American bollworm is the deadliest because the cotton can be condemned on the international market. Cotton may actually be shipped from here to Brazil, but if the strand is found to have been affected by the American bollworm, the cotton will be shipped back or disposed of. You see the cost?

So, won't it have other uses?

They will tell you this it's useless. Then you use if for stock feed. That has serious financial implications on the company and the income of farmers.

During the twenty years that i worked for a cotton company, there used to be bonuses given to farmers. They would get these bonuses when their cotton fetched a good price on the international market, maybe an extra 20 or fifty cents per kg.

Over the years, have you observed any changes in the types of pests found in cotton?

There was a time when we used cruiser. This pesticide was effective against jassids and aphids. For the first 3 weeks the farmer would not get in the field with a knapsack. There is nothing as laborious and painful work as carrying the knapsack. So, if there are cruiser treated seeds, the seeds would have the pesticide that would kill the jassids and aphids?

Is it the same as bt cotton?

No, but they are similar. I'm really not that familiar with bt cotton. With cruiser treated cotton, farmers could dip the seeds in the cruiser and then plant the seed the following day. When it absorbs, and when it germinates the pesticide becomes effective. The moment the aphids attack they will be affected.
Was this cruiser ever used here in Rushinga? Yes it was, and its advantage was that it was effective for the first three weeks.
And was it discontinued?
I think it was discontinued because of the political or economic situation. People are no longer growing cotton as they used to. People have resorted to growing ground nuts and other small grains.
For how long was cruiser seed used in Rushinga district?
It was used during three to four seasons. Cruiser treated seeds were effective. Farmers were assured that three weeks after planting there was no need for any spraying on their crop.
Have there not been any seeds developed to be toxic to the American bollworm?
As far as i know there hasn't been much success in engineering seeds to be toxic to the American. But, since i am no longer working for the cotton company, and i no longer have access to magazines i cannot say this with certainty. But, all that i know is that the only way to deal with the American bollworm is to destock the cotton. What happens is, if you leave the cotton stalks, the American bollworm will come and hibernate in it. When it shoots it can easily attack the crop.
Is there any change in the types of pests in the area over the past thirty years?
No, there hasn’t been any change.
You talked about the issue that people are no longer growing cotton the way they used to in the past. What is the main reason for this change?
It's economical, rather political.
Which crops are people now adopting?
Well, speaking from myself, i now prefer to grow more ground nuts. Early cropping. If you grow ground nuts early, you can harvest something meaningful and be able to send children to school. When i was still working at Cottco, people used to get good returns from their cotton. If, for example a farmer harvested only three bales; one bale would send children to school, the second one would buy a plough, and the third one would buy a cow, and the fourth one would buy inputs for the next season. A farmer would have already paid off their loan with the cotton companies if they had taken a loan. Nowadays, it’s no longer the case. Those farmers who are taking loans to farm under contract have more to lose because the revenue is not enough to even pay off the initial loan, so at the end the cotton companies end up attaching these farmers' properties in order to recover their money.
Which pesticides have been used in the past years?
Agrithane, Sanvalerate, Fenvalerate,
did you say Sanvalerate?
No, when we talk of pesticides, we are talking about carbaryl, eh, there are companies involved here that deal with aphids. If we talk of aphids we are talking about other companies that are on their own end.
Dimethoate is administered against aphids, Acetamark is used against aphids.
There was Agrithane that was used against worms. After Agrithane then there came Sanvalerate, Fenvalerate. These were just names, but the same pesticides. For example, if you are the new manufacturer you may decide that everything will now be named after your company, while the composition remains the same. So, what we are saying is that there came Sanvalerate, Fenvalerate, but the origin was Agrithane.
Okay
That was the gukurahundi.
Then, there was dimethoate. Dimethoate was replaced by Acetamark. Acetamark was just a small powder packet that was enough to spray one hectare.
Karate was also used against worms.

Some farmers say that the pesticides have lost their power, you, as an expert, what is your take on that? Is it about the pesticides or about the pests themselves, or both?
It's to do with marketing. You see, if i was telling you this in business, i would tell you the truth, but not the whole truth, knowing that tomorrow you would return for the rest of the truth.
So, what are you saying?
It's a deliberate thing. They are making the pesticides ineffective, so that farmers would return to buy more. But, the standards association of Zimbabwe would have tested and endorsed something else.
Do you not think that it is the issue of farmers keeping ratoon crops?
Yes, i am talking about the standards association of Zimbabwe, if things are not standardised they are subject to manipulation.
But, pesticides are standardised.
Yes, but they are subject to manipulation.
It's just similar to the bottled water that you buy and drink in Harare. It's got a standards association of Zimbabwe logo, but it is bottled straight from *Mukuvisi* River. If you drink it you will be struck by diarrhoea before you even reach Mazowe. Isn't it standardised. This is Zimbabwe.. Chuckles.
Ratoon crop is just like tobacco. It needs to be destocked and burned. The worm will simply go and rattle on the stalks and lay its eggs. If left, it will become uncontrollable. When your crop grows it will go and affect the bolls. The lint strand will not be strong. When you send it to Brazil they will condemn it and simply tell you that it is not the right grade for our spinners. It is very expensive to ship cotton and then it gets condemned.
Thank You for your participation

CZ 15

Which are the common pests that you encounter?
I encounter the American bollworm and the caterpillars that cut the plant and leaves.
What are they like?
These are grasshoppers that just cut the leaves. And aphids.
There are three types of worms. These are the red bollworm, the American and the green one whose name i can't recall at the moment.
Which pest would you say is the most problematic?
It's the red bollworm. This one is deadly. It starts destroying from when the cotton starts flowering and then if it does not get the flower, it will still get inside the boll and eat from within. It is very difficult to control.
Since you started growing cotton, do you notice any changes in the population of pests that you encounter on your farm?
The change is that they resist if we use the same type of pesticides. So, what we ought to do is that if we use Fernkill for two seasons, for example, we must change to Fenvalerate. If we use the same type of pesticide they will resist and not die.
Are there any years that you just continue using the same pesticide simply because it’s the one that is available, even though you know that you ought to rotate these pesticides?
Yes, at other times due to our ignorance and also just using the pesticides that are available to our disposal; but the consequence is that the pests will grow in their population.
Would you say the population of pests has changed compared to the past?
Yes, there has been a big change; pests have increased in their population. This is because of the changes in rainfall patterns. People are no longer cutting and burning their cotton stumps. Those farmers who do not cut and burn these end up maintaining their ratoon crops which are pests infested. By the time the rains come these worms will already be in the plant. Worse still, the type of pesticides which we were using the previous years are so ineffective, they just do not work and the

cotton all gets eaten by the pests. Sometimes we do not get the pesticides in time, such that when we finally receive our pesticides it will be impossible to control the worms.
And, as far as the types of pests are concerned, are there any changes?
In the past we didn't have problems with the red spider. In the recent years, we have realised that the red spider is increasingly becoming common in the cotton fields.
In your opinion, what is causing the emergence of red spider in cotton?
I think it's because some farmers were mixing tomatoes and cotton, so that when they go to spray their cotton they would also spray their tomatoes. So, the movement of pests between the two crops has become possible.
You said that the way it is raining is making people prefer ratoon cotton. So, do you see there being a clear relationship between how it is raining now and farmers' practices in preferring ratoon crops?
Yes, definitely. Cotton requires six months. For instance, this October we should have already done dry planting. But the problem now is that the rains will only come in December. If we dry plant, by the time the rains come the crop would have dried. If we plant in December, the cotton will not really mature because the season will be short. It will then need to reach June/July for it to be a good crop. So, when people resort to ratoon, the crop will sync with the short season perfectly well. People now prefer to flout the regulations governing ratoon crops in cotton and risk facing the wrath of the law. If ratoon cotton is well fed with fertilisers it will give a very good harvest.
So you said you do not use any chemicals to control weeds, right?
Yes
How about for the control of insect pests?
Against insects i use chemicals yes.
Which ones do you use?
I use the likes of carbaryl-85, Acetamark, Fernkill, lamda, Fenvalerate. We once used Karate, but then it was banned because it was too harmful to human health if you touched it without gloves, or if it just touched your skin. At times people risked, just getting used to it saying that they are now used to it. Protective clothing was only found at the feet and some overalls. Hands would just be bare, and nothing to cover the nose and mouth. No hat either.
So, you say Karate was discontinued because it was harmful to health?
Yes, but all pesticides are all harmful to human health. But with karate you could easily feel its effects. Itching on the skin.
In terms of the quantity of pesticides which you use, would you say there have been any changes in the amount used per hectare?
Well, in the past, not knowing how pesticides are made; we used to use the likes of Thiodan and carbaryl-85. These liquid pesticides were not yet there. With the passage of time, we started using the liquid ones. We would mix Thiodan and Carbaryl 85. Later on the liquid ones were introduced, and we called these ones the Gukurahundi, the type of liquid pesticides.
Would you say there have been any changes in the amounts used?
We reached a point when we used a lot of pesticides, also depending on the size of cultivated land. But, it was also because of 'resistance' found in the worms. We started using a lot of pesticides.
After using a lot of pesticides, do you find the pesticides being effective in controlling the pests?
Like i said, now the worms are very resistant. It's due to such factors as ratooning the crops. The worms go deep in the ground to the root of the plant as a way of dodging the pesticides. When we plant to cotton the plant will already be diseased. Mainly because of these worms and other infestations. We notice that in the past we would spray fortnightly after scouting for pests. And even after the two weeks the pests would not be too much. Nowadays it's different, after spraying today within three/four days you find there are pests.
So, the pests you find just a few days after spraying are they new pests or they are the same pests?
It's the same pests that have just been there, only that they would be resisting.
When you grow cotton, do you get loan on contract or you buy your own inputs?

I have been getting loans in the recent years, but at the beginning in the 1980s we had to stand on our own.
But, with the passage of time Cottco, while it was still under the grain marketing board, started providing loans. Seed could not be bought anywhere except when it was bought via loans. In the past we could travel to *Bindura* to buy seed. But when the Cottco credit scheme, people started to get loans.
Right now, can people buy seed cash without necessarily getting it as a loan?
There is a bit of *kakubatirira* (injustice) there because you need to find a company to give you seed on credit. Then you get yourself into unwanted credits. You may then buy your own pesticides if you wish to have not a huge debt.
What other methods of pest control do you use other than pesticides?
There are some traditional methods that we use against aphids. We have, at times used Surf washing powder and red-hot chillies against aphids.
How do you prepare and administer these?
You crush the chillies and sieve then mix with water and put the water in your knap sack and spray as normal. If we do not have Rogor, we just use chillies to control aphids.
So, are you telling me that the washing powder that we use to wash clothes can work as a pesticide?
Yes, it is quite effective.
There is also another plant that is called *kambanda* that is like marijuana. We have also tried that one. It can be soaked and then use the water as a pesticide. That plant smells a lot.
Is it not the one that *Zezuru* people call *zumbane*?
That is the precise one. Zumbane is the name.
Have you ever received any training of the safe use of pesticides?
Yes, my wife was lucky to be the chairwoman of our cotton group for many years. So she always attended those trainings for chairpersons.
May i now ask these questions to your wife? What sort of training did you receive?
Every year before the beginning of the cotton growing season in October, we always received training from the cotton companies.
Did you receive any training on the management of pests?
Yes, we were taught how to mix pesticides such as Carbaryl for the control of aphids. We were taught so that we would also come back and teach our people. Then when the plants developed bolls we would receive further training on the use of Fernkill, mixing it etc. I would then return and demonstrate to my group. Even how to apply fertilisers, we were always taught.
Did you also receive any education on the dangers of pesticides to human health?
Yes, we received such education as well. We were always encouraged to wear overalls and safety shoes. But, the problem was that the companies only provided overalls and a pair of safety shoes to the chairperson so that i would be able to demonstrate to my group members. They never provided to every member.
They also provided me with a bicycle so that i would be able to travel to the fields of each and every member of my group and do assessments and provide advice. I would then go to the company and give my report, then if there were any issues arising the company would send their representative with a motorbike to the farmers.
Alright thank you.

CZ 16
Which pests are found in your cotton fields?
Here we mainly come across aphids, jassids, red bollworm and the American boll worm as well as certain white flies call bed backs.
Which of these pests is most problems?
The red boll worm is the one that really give us the most problems.

As far as your own observation is concerned, would you say there are any changes in pest populations?
I think pests are not increasing in their population. If you spray your cotton with lambda before the worms are present, they will die. But, if you delay and get in to spray after the red bollworm has entered the bolls, controlling it is very difficult. You will have to return next week and you might find it outside after it has finished consuming one plant. As long as it is inside the boll, it will not die because it will enclose itself inside.
Tell me. I have a question based on your previous reply. Do you spray after you have seen pests, or you spray in a preventive way?
No. What happens is, if we just spray anyhow, those pests will get addicted to the pesticides and in the long run they will not die. So, we first assess in the field for the presence of the eggs of the red bollworm. If we find them we then spray. If the eggs are sprayed they will be affected by the pesticides and they will not hatch.
Are there any changes in pest types?
No, there aren't any changes. The same types that we have always known are the ones that we see today present.
Which pesticides do you use against the pests that you encounter on your farm?
I use Carbaryl -85, Rogor, Acetamark, and Fernkill or lambda.
And how do you normally control weeds?
I have never used any herbicides. I always weed manually. I first use a cultivator, thereafter i use a hoe.
Are there any changes in the amount of pesticides used per hectare?
There have not been any changes; to me the amount i use per hectare has remained stable over the years. But, the cotton companies are the ones that tend to limit the amount of pesticides that they give to people.
So, do you find the amount of pesticides you receive from the cotton companies sufficient to control pests per hectare?
Yes, i always find it sufficient.
Do you sometimes re-grow your cotton stumps from the previous season?
Since i started farming i have never regrown my cotton stumps. If i do the regrowths, i may end up being the loser because those crops need a lot of pesticides. And i may lose a lot of money. Nevertheless, the regrowth crops are quicker; they develop before the new crops.
Do you use other methods of pest control other those pesticides?
Yes. I also kill the red bollworms manually when i find them. If it rains the pesticides may be washed off, so if the red bollworm leaves the boll and encounters no pesticides outside it may continue going and destroying the bolls. So sometimes i physically kill them.
The problem with the red bollworms is that they can cause so much devastation. One bollworm can destroy up to 70 bolls before it dies.
Have you ever attended any courses on pesticide uses?
I did training on cotton farming in general; about land preparation and the assessment for cotton pests and diseases, and identification of the moths that result in the Redboll worm.
Where were you trained?
I was trained just down the road at the Cargill depot.
Were you also trained on the safe usage of pesticides and how they affect your health?
Yes, we were trained, and advised that we must not spray against the wind direction as the wind will be blown towards you.
Thank You for your time.

CZ 17
Do you buy your own inputs for the 1 hectare, or you get on credit?

I get my inputs on credit. Last year i even paid 20 dollars for the preparation of my field because i do not have any cows.

How much did you harvest on your 1 hectare?

I managed just a single bale.

In the past, how much did you get on 1 hectare?

In the past, when we were receiving enough inputs, i could get as much as 3 and half bales on one hectare.

Why is it that the harvest has plummeted?

It's because of the ''supply'' of the companies, and the price at which they are buying from us. When they come, they try to woe us by saying they will support us with all the inputs. But when it comes to the supply of the inputs such as the chemicals and the fertilisers, they then say they do not have, they cannot supply us and then we start to look around for these.

Are you saying, the more the inputs, the better the harvest?

Yes, particularly the fertilisers, from compound L, and AN, and the chemicals.

The companies just come and make us sign contacts and then just give us seed. They then promise to supply the chemicals and fertilisers later, while the season is already going, and then you grow your crop hoping the companies would honour their contractual obligations and supply the said inputs timeously. But, then they just later on tell us that they cannot supply anything else.

Which pesticides are common on your farm?

In the beginning, there are jassids. These suck the sap of your crop from the leaves. If you do not control them you will see your leaves wilting and looking unhealthy. You should control these jassids with carbaryl-85. The cotton companies do not supply with this. So, this instantly affect the growth of the plant. So, when you plant your cotton you must already have your carbaryl-85 at hand. You must also have at hand Acetamark, which is used to control aphids. These aphids also suck from the leaves.

Which other pests do you encounter?

In addition to the aphids and the jassids, another pest is the red bollworm. The red bollworm feeds on the bolls of the cotton. It must be controlled before it gets inside the boll. Then, there is the American bollworm, which is green in colour. This one is easier to control that the red bollworm because it feeds from outside, unlike the American which feeds from within the bolls. When the red bollworm enters the boll, it seals its entrance, which makes it very difficult to control it. The best way to deal with the red bollworm is to control it while they are still eggs laid on the leaves. The eggs are quite visible.

Which pests do you use to control the red bollworm?

In the past when we were young, my father mentored me using Fernkill. After Fernkill, they introduced the one that we are now using, called Lambda. Before lambda, there was Ferncure. During that time of *Ferncure*, another brand was called Shasha and then another one was called moto. But, nowadays, since 2010, we are using lambda.

Which pest would you say you have had the most problems with?

The most problematic pest which severely affects our harvest is the red bollworm. This is so because once they eat, the boll is destroyed and lost.

Are there any changes in the amount of pesticides used now, compared to years in the past?

We now use less amounts of pesticides. The supply that is coming from the companies is now a bit crooked. In the past, once you are contracted, you knew that the company would give you all the inputs needed to produce on your hectare all at once. During those days, around this month of September was when those companies contacted people, and they would deliver the inputs, all the input requirements, including fertilisers. They would give complete inputs commensurate with the size of land for which you would be loaning.

So, if the amount of pesticides has declined, is it enough to control pests?

No, it’s not, particularly this one that they call lambda. It does not do anything to the worms.

Would you say it is to do with the pesticide, and not to do with the pests themselves?

No, it is something to do with the pesticide.
The increase in pest populations is because the pesticides are not able to kill.
Do you have any other concerns with regard to pests on your farm?
The concern that i have is that the pesticides are not controlling the pests, so the population of pests is increasing.
Are there any changes in as far as the types of pests found on your farm are concerned?
Here i have never noticed any new pests. However, those who grow cotton near the Mozambique border in the *Mafigu and Nyatsato* areas, that there are new types of moths that are destroying cotton. And these new moths are ''resistant'' to the pesticides.
Which other pesticides do you use?
What i know, in cotton farming is that, at first, you must use Acetamark, after Acetamark stage two, you use carbaryl-85, after carbaryl-85, we need what we need the purple triangle pesticides such as the likes of Lambda, Ferncure, Fernkill.
The way i see it. These purple triangle pesticides, such as Lambda, have effect on the health of people. The moment the pesticide has contact with the skin, or when spraying and the nozzle is tipped, and the pesticide makes contact with the face or the skin, it itches in a way that really hurts. As peasant farmers, we do not have the ''combat'' that should protect us from these pesticides. Just the way i am dressed right now, I am good for spraying any pesticides. My knapsack may just spill some pesticides, and i may suddenly get hurt. We sometimes request the companies to include personal protective equipment in their loans when they extend inputs to us, but they always make false promises. But, there is only one company that once responded; Cargill, by simply providing some breathing masks that just close the mouth and the nose.
Do you use pesticides on other crops?
No. With the rest of the crops, there is no need for any pesticide use. We only need to weed manually with hoes. But with cotton it’s different. The field must be very clear of any weeds.
Which weeds do you encounter on your farm?
Here we have problems with chaguduma and chinzungu.
How do you control it?
Here i have never seen anybody use Cotguard, and i do not have an idea what colour it is.
So, how do you control weeds on your farm?
I always weed manually.
Have you ever gotten training on safe use of pesticides and other methods of pest control?
No, i have never done anything as far as training is concerned.

Thank You for your time

CZ 18

Which pests are commonly found on your farm?
The main ones are red spider mite, the American bollworm and the red bollworm.
With which of these have you had most problems over the past years of your cotton growing?
It's the red spider mite because the pesticides to use against it are not readily available. We no longer find the pesticides to use against it. Once it have entered your field you are done.
Oh, so the main issue is that the pesticides are no longer found?
No. They are there, we can find them, but the problem is that they are no longer powerful enough to kill. In the past we would use three bottle of liquid pesticides and one bag in powdered form. Once spotted, we would just carry our knapsack and bomb it. Nowadays, it seems the population is increasing. It’s a real problem.
Have there been any changes in the amount of pests on your farm in the past 30 years?
Pests’ populations are increasing because people are no longer cutting their cotton stalks. Therefore, the pests are developing resistance because they stay there. They do not die because these stalks

will not have been cut and burned. The worms will have stayed in the stalk such that when you attempt to kill it using pesticides it will not die.

Are there any changes in the types of pests?

Yes, there are also new pests. It's only that i do not know some of the names. But, the ones that are known are the likes of red spider mite, red bollworm, and the American bollworm. Some of the newer pests, i don't know their names. There is one that is green-like with yellow stripes. One of the worms is called *kambasha* after the colours of the bus that used to ply our Rushinga route many years ago. There are also new types of aphids that are now colonising our fields whose specific names we are not sure of. Then there are other pests that are striped that cut the cotton, but the names are the problem for me.

What might be the cause of these changes?

Why pest types are increasing is that people are not taking care of their stalks. in the past we used to cut and burn our cotton stalks soon after harvest, and by September everyone would have burned their stalks. Nowadays, you find these stalks still standing in the fields. Therefore the pests are just there. When we spray them these pests would be very resistant. To say you have killed them, what sort of pesticide would you have used? The pesticides that we have these days we always say they no longer work; of course so, how would they if these pests are left to survive through summer growing season, winter and again another growing season?

Looking at weeds; what are the main types of weeds that are found on your farm?

There is a weed that is whitish in colour. We manually weed it and throw it away. Other common types of weeds that we encounter are *bwerere*, and chaguduma, and *moyochena*. Then there is another one called chinzungu, which is the most problematic for cotton farmers.

What other crops do you grow?

I also grow maize and groundnuts.

Do you ever mix your crops in the same fields?

No, we cannot mix cotton with any other crops because of the pesticides we use. If we mix with cucumbers, for example, children might go and pick them and eat unbeknown to them that there are pesticides on the cucumbers. Our agricultural extension officers always remind us that we should never mix crops.

Has the acreage of cotton changed relative to other crops?

The land needed for cotton is always larger, if you are to realise a good harvest. You need at least 3hectares.

How much do you normally harvest per hectare?

Well, in the past when we used to get enough fertilisers we used to realise as much as 6/7 bales per hectare. Nowadays, because we no longer receive enough fertilisers, our harvests have declined. We are now realising just enough to survive, just about 2/3 bales per hectare.

Which pesticides do you use on your farm?

We first use Dimethoate, or Mospilan. It's still the same pesticide, only that Mospilan is in powdered form. We use it against aphids.

When the plant starts to flower, we use Carbaryl 85. This is used against red bollworm.

Thereafter, we use karate, or this other one that is itchy. I do not remember the name, but it is in the same family as karate. It is very itchy.

Has the amount of pesticides used over the past thirty years changed?

The pesticides that we used in the past were very strong. We used to get 200ml, and we would use the same small bottle till we harvested. But, nowadays we have the types of Lambda, that's the pesticide whose name i had forgotten, the one that itches a lot; we get 500mls of it. But, just that one 500ml bottle is not enough. You need four/five for you to be able to harvest, which means the pesticides that we are getting nowadays are very weak, they do not have power.

Now that you are using more, would you say you are having sufficient to kill your pests?

No, it's not sufficient. We really need more pesticides. At times when we scout and see that there are pests, we just ignore and pretend as if we do not see anything so that we conserve our pesticides

so that they can last us the season. However, under normal circumstances, cotton should always be sprayed at the sighting of pests. This is because pests do not stop eating our crop, day and night. But, because the pesticides are not that plenty, we have no other choice than to just pretend as if we do not see that there are pests.
Do you use any pesticides to control weeds on your farm?
No, i do not. I always weed manually. I once tried using herbicides, but i realised that i have little knowledge about them.
Do you use any other forms of pest control?
No. I only use pesticides because there is no other way that the pests would die except by the use of pesticides. They are only killed by pesticides.
Have you ever received education of the usage of pesticides?
We receive education from our agricultural extension officers.

Thank You

CZ 19

Which pests do you encounter on your farm?
There are many pests. There are worms, there are red ones which are not long, and then there are green ones which are long, and those that are yellowish which are long. The red ones are the ones that bore the bolls and get inside the cotton bolls. The long ones feed from outside.
Of these, which one would you say gives you the most problems on your farm?
The red worm
Would you say that since you started growing cotton there have been changes in as far as the population of pests are concerned on your farm?
Yes, these have been increasing steadily over the years. The past two seasons have been the worst. They even do not respond to the pesticides.
What do you think is the reason why they no longer seem to respond to the pesticides?
I think it's because the pesticides no longer have power.
I also think it's because these cotton companies when they give input credits to us they also give us expired pesticides as they cannot afford to lose money by throwing them away. When we use these pesticides they will not work because they would have expired.
In terms of pests types, are there any changes that you notice with regards to the types?
The long ones, the green ones and the yellowish ones are the ones that seem to be increasing in their numbers.
Which weeds do you find on your farm?
There is chaguduma, or chidhongi, and then there is also one called *chinzungu*.
How do you control these weeds?
We always exclusively weed physically using hoes. We never use any chemicals.
Which other crop do you grow?
We also grow maize, and ground nuts, and mhunga, mapfunde and cow peas.
Has the acreage of cotton changed relative that of the other crops over the years?
Well, the acreage always varies each year depending on how much we desire to grow.
Alright. So, tell me which pesticides do you use?
There are Carbaryl 85, Rogor and the gukurahundi.
Do you buy these pesticides or you get them on credit?
We normally get them on credit
Looking at the amount of pesticides you use, over the years would you say there are any changes in the amount of pesticides you use?
These days we use more pesticides than in the past. Usually the amount of pesticides that they give us are not enough, so we end up going to buy extra bottles. In the past where we used three bottles

we are now using double that amount, but the worms are still not getting finished. We end up following them and identify where they are and then kill them physically.
Why is it that there is such a big problem with these worms?
I think the pesticides have lost their power.
Other than pesticides, do you use other methods of pest control?
We only use pesticides
Have you ever trained or received education on pesticides?
No, we have never received any education. But we only know about the effects of the pesticides, for example we know that if there is a spill on your skin you feel it itching. When that happens we get a piece of soap and quickly wash it off so that the itchiness can quickly end.

Thank you for your participation

CZ 20

Which pests do you encounter on your farm?
We come across jassids. These cause cotton leaves to become wilting. Then there are aphids. And green worms, and then there are red worms that we see when the cotton has developed bolls. This type stays inside the bolls and start to feed from within. If you break open the boll you can see it.
Which pest is most problematic for you?
I find aphids most problematic because this area is a very hot area. So, if there is a two week dry spell we normally start to notice aphids. But once there are bolls we then also start to have serious problems with a lot of the red worms.
Are there any changes in the types of pests?
Yes, there is a new type, a black flying insect. That one stays in the forest but i have noticed that it is increasingly visiting cotton. Its effect is that it cuts the cotton stem, just the top part that grows going up. It can cut several plants. Its' rare to find it in the act, you only see your cotton cut. But i was lucky one day when i saw it. Its black in colour, but it also has a yellow pigment. I wonder which pesticide would affect it.
Which weeds do you come across on your fields?
Here there are several types of weed that are present. There is chidhongi and bwerere. Bwerere grows tall and can pose stiff competition for nutrients with the crops. There is also another type called chinzungu which grows laterally, as opposed to bwerere which grows vertically.
Do you use any herbicides?
No, we normally use hoes and weed manually. But, our agricultural extension officers always encourage us to use herbicides. They say the advantage of herbicides is that it lessens our burden in the field as we will not have to go and spend several days in the fields weeding. They say the use of herbicides will slow us growing old. But, despite their encouragement, we have never used herbicides. They cost money.
Which pesticides do you use against pests?
We use Acetamark, carbaryl-85, and in the past we used to spray with Fernkill. It's the same pesticides, but these days they use ''*zhing-zhong*'' pesticides. The smell is the same, but the names have changed, and the power of the pesticides has also changed. Nowadays these pesticides do not have power.
Has the amount of pesticides you use per hectare changed over time?
We are still using the same amount because they are still giving us the same number of bottles per hectare, but we observe that in the rate at which the pests are dying is now different. In the past the pests died completely, but with the types that we are now using we still struggle because the pests always remain on the fields. That is why we often ask whether the new pesticides are now less powerful compared to the ones that we used in the past. No one has ever told us openly that these days they are making pesticides that are less powerful for us. But, the measurements that we use

for the concentration of pesticides in our knapsacks are the same as those which we used many years ago, nothing has changed.
Do you use any other methods of pest control, other than the usage of pesticides?
No, i always and exclusively control with pesticides.
Have you ever attended training on pest management?
No, i have never, but we have agricultural extension officers here who always teach us about proper farming.
If you go and get the extension officers and bring them to your field they always come and offer you practical advice.
Have you ever received any education on the dangers of pesticides?
No, we have never had such education

Thank You for your participation

CZ 21

Which pests do you come across in your cotton fields?
There are aphids and worms. Some of the worms are green while others are red.
Which one gives you the most problems?
The red one is the one that is really problematic
Have there been any changes in the population of pests?
In the past they were much fewer. In the recent years the pests have been increasing in population.
What has been the cause of such an increase?
It’s mainly because we haven't been receiving enough pesticides from the cotton companies. We have then resorted to moving in the field to physically capture the worms and then kill them using a wire. If you do not see the worm it can eat all the cotton bolls in the vicinity.
Which other crops do you also grow?
I grow maize. I also intend to grow groundnuts as my main cash crop.
Which weeds do you come across?
There is chaguduma and another type which has spikes. If you do not weed that one out then be assured that you will not harvest any cotton where it exists.
Do you use any herbicides?
No, i always weed manually.
Which pesticides have you used for pests on your farm over the years?
It’s mainly Acetamark for aphids and Fernkill for worms.
Has the amount of pesticides used been changing?
Yes. We have used less amounts over the years because we have also been receiving less. That is why i have resorted to killing the worms physically.
Have you ever been educated on cotton farming?
Yes, i once attended training at Cottco.
Did they also educate you on the dangers of pesticides?
No, they did not.
You already talked about the effects of pesticides, i think i have exhausted my questions.

Thank you very much.

CZ 22
Which cotton pests are commonly found on your farm?
There are red worms and then the green ones. But the ones that cause the greatest destruction are those that eat the bolls.
Have there been any changes in terms of pest populations in the past 30 years?

It seems there have been increasing numbers of the red ones.
Are there any changes in terms of the types of pests observed over the past 30 years?
No, i have not seen any changes
Do you have any other concerns?
No concerns about pests. It's just that the cotton is not paying well. How can a woman of my age carry a pump on her back only to produce for a cotton company?
Which weeds are found on your farm?
There is chinzungu, bwerere, and then this one which started appearing recently, here we call it chenamoyo. Then there is also chaguduma.
Which other crops do you grow?
I also grow maize, groundnuts, *mapfunde, and mashava*
Which cotton pesticides have you been using all these years?
We have used Gukurahundi, Acetamark, and 85 Carbaryl.
Have there been changes in terms of pesticide requirements on your farm over the past 30 years?
In the past when we got input loans, we would get enough pesticides. If i got a loan for 2 hectares and then decided to grow three, then it would not be enough. In the past they would give us enough. Nowadays they no longer give enough pesticides.
Are the pesticides used these days enough?
No. The pesticides no longer have power. We had the pesticide called Thiodan, that one had so much power.
Which herbicides do you use?
We have never used any herbicides. We always weed manually.
Do you use other methods of pest control other than the use of pesticides?
No. We have always used pesticides.
Have you ever received education on other methods of pest control?
No
Do you receive personal protective clothing from cotton companies?
No
How do pesticides affect your health?
When you spray, if the pesticides blows on your face, it feel burning on the face and the eyes.

Thank you for participating

CZ 23

Which pests do you come across on your farm?
There are several. We first encounter aphids. Then there follow, these ones, what are they called, by the way!
Which pest would say is most problematic?
It's the red bollworm
Are there any changes, in the past 30 years, in the types of pests found on your farm?
Yes, in the beginning the red bollworm was not as much as it is now. The population numbers have significantly increased in the recent years. And there has also been changes in the types of pesticides used against it.
And which weeds do you find in your fields?
There are broadleaves, such as *chidhongi, chinzungu and chenamoyo or feso.*
Let me ask something about the red bollworms, when do you spray them? Is it when you see them or when you see the eggs?
We spray when we see the eggs. The eggs won't take time to hatch, so if we spray on the eggs the eggs will die, of if they hatch they hatch and immediately encounter pesticides. If you do not find the

eggs you will have to spray as the worms. But, the disadvantage is once it becomes a worm it gets inside the boll. When it leaves one boll it gets inside another one.

Which pesticides do you use against pests in your field?

At the beginning we use 85. This is used before worms start appearing. 85 is also good for the plant, it seems it also contains a fertiliser; plants grow well when they are sprayed with 85. This pesticides is applied to control aphids. Thereafter, there are the likes of Lambdar, the Rogor. Rogor itself is no longer used, Lambda is now the one that is in use. Then there are the Fenkill, used against worms. Then there is Acetamark which is also used against aphids.

Okay, so are these the main pesticides which you use?

Yes, these are the ones which we use, but there are indications that things are not well.

Alright, so please would you tell me. In the past thirty years, are there any changes in the amount of pesticides used over the years?

Yes. We are now using more pesticides.

What is the reason for the increase in pesticide use?

I think its because people are now doing regrows. And these keep hosting pests. If the neighbour's farm has regrows, pests from their farm will cross over to my own farm. It's because when they do not destroy their cotton stalks, pests will keep surviving in these stalks. Last year but one, for example, we ended up observing that some plants that had already developed several bolls started to develop a disease that appeared like mutation of plant cells. After five days to a weak the plant would wilt and eventually just dry. Some say it is caused by the worms which would have survived in the ground. When we cut the plants right where there appeared to be diseased, we found that there were some small white worms.

Okay, so are these some kind of new worms different from the ones which you have always had to deal with in cotton over the years?

Yes. They are new, they started only last year but one.

Is this a problem that you have heard other farmers also talk about, or its just peculiar to your own field?

Other farmers have also talked about sighting these same worms affecting their cotton crops as well.

Have you informed your agricultural extension officers about these worms?

Yes, we informed somebody.

Okay.

This problem started only recently. If you inspect the plant, you will see that there will not be any pests on it, but if you look down where the plant meets with the soil, that's where you notice that the plant will have developed some kind of roughness. If it were a human being, we would say they would have been affected by ringworm *(chisasa)*. If you pluck the plant and break it from the point where it is affected, that is when you discover that there would be small white worms, and the appearance of the plant stem would resemble that which would have been affected by termites. They will just be localised at that point only and not all over the plant. But, despite that they affect the plant in such a way that we end up losing plants which would have developed a lot of bolls.

When that happens, do you harvest these bolls?

No, they will be just bolls which would have not matured at all.

Have you used any pesticides to try and target controlling these worms?

No, we haven't yet since this problem appeared just recently.

Even a very big plant, if it's affected by these worms you start noticing it giving in by starting to wilt.

Have you ever been educated on the harmful effects of pesticides?

Yes, when i did my cotton farming training we were educated on the dangers of pesticide exposures. And the effects may result in bearing a disabled child. That's when you find people trying to find a witch after having born a disabled child and yet the cause would have been the pesticides which we use.

Are there other methods of pest control that you use apart from the use of pesticides?

No, there are any other methods. Instead, we were actually encouraged to use herbicides instead of manually weeding so that the work becomes less physically strenuous. We were also taught about the different types of sprayers; some have advantages of being light weight, others have advantages associated with being strong, while others have the advantage of the ability to spray a large area, for example spraying four lines when going in one direction, especially those that came from Brazil. Then there are those who operate large commercial farms who spray using planes, then those who use what appears to be a bike.

Thank You for your participation

CZ 24

Which cotton pests are found on your farm?

There are aphids, then the red bollworm, which is most problematic. There is also a green worm which feeds on the flowers before they develop into the boll. The red one eats the bolls, then it lays its eggs inside the boll and then they can instantly multiply.

Among these, which one has grown in population?

In my experience as a cotton farmer, in the past we used to have a pesticide called rogor, then 85, then Thiodan, and the other one the fourth one whose name i can no longer recall. These worms are sprayed at following certain stages. We used rogor in the past. After rogor and the rest of the pesticides that i have talked about, there then was a powdered one called Acetamark. This one was different from rogor. With rogor, we would measure the lid of a bottle and mix with 15 litres of water in the knapsack. With the Acetamark, we measure a teaspoon and mix with 15 litres of water in the knapsack. Then we spray. The pesticide we used in the past seems to be outdone by the Acetamark in killing aphids.

So, is this Acetamark powder a recent pesticide?

Yes it’s a recent one. We no longer buy rogor.

Then 87 is used to control the green worm. If we spray with Acetamark, we then spray with 85 so that the leaves would open up so that the green worm cannot get a change to attack the crop. Thereafter, we use what we call the gukurahundi to kill the red bollworm.

Which of these pests would you say is the most problematic for you?

For me the most problematic are the aphids as well as the red bollworm.

Over the years, have you noticed any changes in pest populations on your farm?

Let me take you back, in answering you. In the past when we were growing cotton, there was no talk of having to make cotton cuttings. Which they are also calling ''regrowths''. In the past, it was less prevalent. We would harvest our cotton, then cut the cotton stalks and burn them. Nowadays, people no longer cut their cotton stalks. They are now just having regrowths. The worms are now remaining in the soil. When it rains, the cotton stalks immediately shoot, and the worm would be there, already having laid some eggs. That is the reason why the red bollworm is increasing in its population. It is no longer responding to pesticides. It is now surviving during both the planting season and the off season.

Have there been any changes as far as the types of pests found on your field are concerned?

Yes, in the past we used to apply a lot of pesticides compared to nowadays. So, there are some changes that are happening, in the past we used to labour a lot carrying the sprayers. There are now also new sprayers that are less physical; you simply insert some batteries and start to spray.

So, tell me, why were you suing more pesticides in the past?

In the past the pesticides were powerful, but the pests and diseases were also very few. Nowadays, the pesticides are powerful because they are changing the pesticides each year. The pesticides which we use this year are not the same ones that we will use next year.

Are there any other concerns about pests on your farm?

I think we really need new pesticides that can effectively the red bollworm because the pesticides that we currently have are no match to this worm. When we spray, and then when we return to the field to scout, we find it, and then we end up looking for its presence and killing it manually.
Which pesticides have you used on your farm?
The pesticides that we used in the past are Rogor, Carbaryl 85, Gukurahundi and another one which i do not recall. But these are the four pesticides which we received from the company Agricura. Nowadays people just use the Acetamark which i referred to earlier and gukurahundi and 85. The three of them are the ones that we are now using.
Would you say that the amount of pesticides you use have changed over the years?
Yes, in the past we used to use many pesticides. Now, we know better. We now know at this stage i use that pesticide, at that stage i use this pesticide, and then towards the end i use Gukurahundi and again that Acetamark again. The reason why we use it towards the end again is that those aphids get into the cotton. If you examine it, it may be discoloured, or have a black colour; that is the cotton whose bolls opened up while there were some aphids. Then there are also some pests that are red, which multiply very fast, that are also found in cotton. We also spray with Acetamark in order to kill these so that they do not affect the quality of the cotton.
Would you please describe these pests?
These pests are red in colour. We call then by the name *vana Rudo* (ladybird beetles) because they travel in a group. They can be 50 grouped together.
Are the pesticides which you are currently using enough to control pests on your farm?
Yes, but if used properly. The problem that we face is that we grow cotton on 2 hectares, and then receive only two or three bottles of pesticides which are not sufficient.
If they are not sufficient, what do you do?
That's when you see then your crop being attacked by the pests
Are you not then buying extra bottles with your own cash resources?
No. But, if you follow instructions and use the pesticides properly everything will go well because on 1 hectare you must use 2 packs of Acetamark, then 2kgs or Carbaryl 85, and three bottles of guku
Other than using pesticides, are there any other methods of pest control that you use?
No, there aren't other methods which i use except using pesticides. Other people, when they talk, say that there are other ways, especially against aphids. Do you know the plant called *nhundurwa*?
Yes, *nhundurwa*, the yellow plant?
Yes, they say, you take it and crush it, and then you mix it with Surf washing powder in water, and then use it for spraying. Aphids will die.
Alright. Does this work?
Yes, it works. If you spray vegetables with it, aphids will die.
You may also take the urine of cows. It also works to kill aphids.
But these are our own community science, kkkkk
Yes, it's this comminity science that we actually want, kkkk
Yes, that is what we do. Others say you take; you know right we peel maize from the chaff. If we burn the chaff into ash, we take that ash and put in water and then sieve and use the water to spray. It itches when it gets into contact with the aphids.
Have you ever received training in the ways of managing pests on your farm?
Yes, we have received trained by the agricultural extension officers.
There is another question that we did not ask earlier on. Which weeds are found on your cotton fields?
There are several types of weed that are found on the cotton fields. There is *chinzungu*, there is *chidhongi*, and there is *sawi*.
How do you control weeds?
I do not use any herbicides, i only weed manually. We use a cultivator, thereafter we get into the field and weed with hoes. So, the reason why we do not want to use any herbicides is just one or two; we will not be able to get any pumpkin leaves, we will not be able to eat ay cucumbers, we will

not be able to eat any pumpkins because once you spray with any herbicides it means there will not grow anything else except cotton or maize. But, us wanting cucumbers, pumpkins and pumpkin leaves, we do not spray.
Thank you very much for your time.

CZ 25

Which are the pests commonly found on your farm?
The first ones to attack the crop are the aphids, which are then followed by the red bollworm and the American.
Are these all?
Yes, they will be mixed, the leaf eaters and the spy(inaudible)
Which among these pests would you say have changed in their population over the past 30 years?
The American seems to have significantly increased.
Have you observed any changes, over the past 30 years in as far as the types of pests found on your farm is concerned?
The types are always the same. There have not been any changes.
Are there any other concerns concern pests on your farm?
All that i can say is that, you see. If you take a drum of red sea and pour one shovel of sugar in it, you will not be able to taste the sugar. What i have observed is that farmers are a bit too ignorant; when the agricultural extension officers tell them to mix a certain amount of pesticides with 15 litres of water, they always make mistakes. But, if a farmer makes the right mixture, the pests will die.
Which weeds are commonly found on your farm?
There is one that we call *chinzungu*, then *bwerere, and chaguduma*.
Which other crops do you grow on your farm?
I also grow maize, sunflowers, *mashava* and groundnuts
Has there been any change in as far as your cotton acreage is concerned relative the other crops?
Yes, i have actually decided that i should completely stop growing cotton, and concentrate on the other crops. There have also been major changes in harvest per acreage mainly because of the changes in weather conditions. The harvests that we used to have in the past when we had more rains are now so different from the harvests that we are currently having per hectare.
Which pesticides do you use on your cotton farm?
There is Acetamark and Dimethoate, these are all Rogor. Then there is carbaryl-85, then there are likes of fernkill, these are now the gukurahundi pesticides and fenvalerate.
Has the amount of pesticides used on your farm changed?
No, there haven't been any changes. It's basically the same amount.
Are the pesticides used sufficient to control the pests?
Yes, they are enough, if scouting is done well. There are actually even some left overs. Pesticides are not to be sprayed weekly even when there are no pests.
Which herbicides have you used on your farm?
I have never used any herbicides, i always weed manually.
Are there any other methods of pest control that you use?
No, there are no other methods that i use. I only use the pesticides.
Have you ever received any training or education on the safe use of pesticides?
No, i haven't
Have you ever received any ppe from cotton companies?
No, i have never been given any. But, they should give together with the input loans, and then farmers will just pay together with the inputs when they deliver their crops.
Do you use pesticides on the other crops that you grow?
Yes, in maize i use a pesticide called dicteret against worms, and then in mashava i use Acetamark against aphids, and in cow peas i use 85.

When you use these pesticides, do you use the same pesticide proportions in your knapsack as those used when spraying cotton?
No, the concentrations vary with the crops. I consult with the agricultural extension officers and ask what sort of mixtures to use on various crops.

Thank you for your participation

TM 01
Pests found in the fields
There are Red spider, aphids, red bollworm, and American boll worm.
The aphids stay on the leaves and the suck the sap out of the leaves. The red spider damages cotton in that the cotton plant will not be healthy because it sucks and that leads to change of colour; the red bollworm is brown in colour. It enters inside the bolls. When it enters it will be very small, and it will grow big whilst inside the cotton boll if there are no pesticides to destroy it. The American bollworm is a worm with zebra-like colours. It feeds from outside. If there are no pesticides to control it, it can cause the loss of many bolls, which may result in the farmer losing his crop. Then there are also flying insects, they are small and white in colour. They also suck on the plant. Then in the ground, there is a white worm that goes onto the root stem and then interferes with the flow of nutrients up the stem of the plant. If the cotton stalks are not destroyed and burnt, this is the worm that can become a problem.
Which of these would you say have increased in population over the years?
The red spider, the American bollworm, and the red bollworm have increased their population.
Any changes observed over the past thirty years?
I have observed that diseases are increasing due to the fact that the chemicals are now very weak on aphids.
Changes in relation to pests?
There have not been any observed changes because the pests were eating from the ground.
Do you have any other concerns with regard to pests on your farm?
Yes. In as far as i have observed, i am convinced that the chemicals that we are now using have lost their power. The chemicals are no longer suitable for the worms that are there. Could it be resistance? I wonder! But, i have observed that even when i tried to grow cotton in a field that i had never before used for cotton, i realised that when i sprayed for pests expecting to return after 14 days, within three days i would notice the presence of pests, clearly meaning that they never died. And, that means that the pesticides are weak, they are not effective in killing these pests at all.
Which weeds are found in your cotton fields
There is *chidhongi, and chinzungu.* These are really tough.
Do you also grow other crops apart from cotton?
I also grow ground nuts, and maize, and cow peas.
Have there been any changes in the acreage of cotton relative the acreage of these other crop over the years?
Let me say that five years and back, cotton was of so much significance to me. My household was supported by cotton. Even my family survived on cotton. I bought my livestock using income earned from cotton. But, in the recent years the fact that the pesticides have become so weak, i cannot count on cotton any longer for survival.
Which pesticides have you used over the past 30 years?
When i started cotton, before we started using Fernkill, we had a very powerful pesticide whose name i am forgetting. It was so powerful that if it dropped on your feet you would itch. We then used Fernkill for many years from the year 2001. Then recently, after 2008, we then changed the pesticides. But the names are just far from my memory at the moment.
Have there been any changes in the amount of pesticides used?
Yes, there have been changes? The pesticides are now so weak.

Are the pesticides enough/ sufficient?
No, they are not.
Which herbicides do you use?
I have used Paraquat. But, i normally do not use herbicides, i always weed manually.
Do you use any other methods of integrated pest management?
No, there is no other remedy for cotton except pesticides.
Have you ever received any education on pesticides?
Yes! The first one is that after harvesting, we should destroy and burn all cotton stalks as a way of controlling pests.
Secondly, we were taught that we should not grow cotton in the same field for two consecutive seasons. We must rotate.
Thank You for your participation

TM 02
Which cotton pests found in your fields
There are plenty of pests. But, the problem is I'm not educated, i wouldn't know these pests by their names. There are those that damage the stem, and i use 85, to kill them. When the cotton starts to build bolls, i start to mix 85 with other chemicals.
There are also aphids, which i kill with Acetamark. Then around January i use Gukurahundi.
Which pests have increased in population over the past thirty years?
In my opinion aphids have increased in population. They damage the crop even before it has developed bolls. They also attack the bolls.
Are there any changes you have seen in terms of population?
I have not really noticed any changes
Have there been any changes in the types of pests?
Those with enough pesticides are able to kill and destroy pests. if you do not have enough pesticides you will not win the battle against the pests.
Which pesticides have you used over the years?
We started with Carbaryl 85 since the 1980s,
When we saw aphids we would use Acetamark, and then later gukurahundi, in the past there was still Thiodan. It's not available anymore. We are now using gukurahundi and other chemicals that we are using to kill these pests.
Has the amount of pesticides changed?
I think the pesticides are the same; it's only the names that have changed. The pesticides are still strong, it only calls for one's knowledge to use the pesticides.
Are the pesticides that you are currently using enough to destroy?
Yes, they are. They are very strong. If you, as a farmer, think that the chemicals are weaker or not enough, then do not grow your crop on a large scale so that your chemicals can be sufficient.
Do you use any herbicides?
I have never used any herbicides. I have always used my cultivator to weed and then i would also use the hoe.
Do you use other methods of pest control?
No. I only use pesticides. I do not know other methods. If i do not have money for pesticides i would rather sell a goat and use the money earned to buy pesticides.
Thank You for your participation

TM 04
Which Cotton pests are found on your farm?
For me the most problematic pest is the red bollworm. These destroy my cotton because we always receive Fernkill very late. They say we should use Fernkill after March. But, the problem is that I grow my cotton early, and so my cotton flowers and develops bolls much early. The problem is that I have no defence against worms which feed on the bolls. So I just administer cabaryl-85 and

Acetamark, which are inappropriate pesticides to use against the worms. So worms are the most detrimental pests for me.
Which pests have increased their population over the past 30 years?
There are ladybird beetles, and then there are also the red bollworms and the other ones whose names I don't remember; but they are worms. They are the ones that are destroying my crop.
Have you noticed any changes in terms of population?
Yes, they are growing in their population. In the past they used to accuse us of destroying cotton stalks late, but in the recent years, I am always destroying my stalks even before I have sold my cotton, but I do not see any decline in population sizes. In fact, they are actually multiplying their numbers. They are now used to the pesticides that we use.
Have you observed any changes in the types of these pests?
No, I haven't noticed any changes in the types. They are the same pests that we have always been dealing with over the past decades.
Are there any other concerns with pests?
I wish we could receive our inputs timeously so that we can see if these pests are just there because they are not being targeted for destruction timeously, or because they have developed resistance.
Which pesticides have you been using since the 80s?
In the beginning when I started, I was using Rogor, cabryl-85, and gukurahundi. Thereafter, things changed and then we started using Rogor, Acetamark, Carbaryl, and Fernkill.
So, there days Fernkill is the one that is mainly used?
Yes, but it causes severe itching such that when you use it you must wear plastics to prevent any skin contact. If you work without any protective clothing, you will not be able to sleep well in the night because the whole body will be ''burning''.
In terms of the quantity of pesticides used, are there any changes?
Pesticides have been reduced. These days we are given only two bottles of Fernkill per hectare. But, the proper way to do it is that they should give us three to four bottles per hectare.
And in terms of types of pesticides, have they changed?
No, they have not changed. Our only request is that they should make them stronger so that we can be able to kill pests.
Which herbicides do you use?
No, I don't use any herbicides; I use my hoe to weed with the help of my wife. I do not even need the herbicides because they damage the soil and make it infertile.
Do you have other methods of pest control that you use?
No, I do not use any other methods. The only method that we were taught was to slash and burn cotton stalks at the end of each harvest as a way of controlling pests. We are doing all that, but we do not see any effect that that is having on pest populations.

Thank You for your participation

TM 05
Which cotton pests are found on his farm?
There are aphids *(inda and nhata/zanguku)*... Then there is the American, and then the red bollworm, and then those that are yellowish. Then there is the pink bollworm which we used to see in the past. We haven't seen it in the recent years.
Which of these pests have increased in population size?
The red and the American bollworms have increased. These two are not declining, they seem to be increasing.
Have there been any changes with regards to pests in the past thirty years?
Well, the only change that i notice is that they are increasing in population. If you spray, it takes only two days to see them again in the field. In the past we would go for a week or two after spraying before we started spotting them again. At times after two weeks you would scout and find nothing. But, these days, no matter how much you spray, these pests are always present.

Are there any changes in pest types, in addition to population numbers?
I do not see any changes. The same types that were there in the past are still the ones that i see today. No new pests.
Are there any other concerns regarding pests on your farm?
Well, it's only that the American and the red bollworms are becoming more problematic.
Which weeds?
There is chaguduma or chidhongi, and then there is also a type of grass called mhande and then another one called *nyabvari.* These are the types that we have problems with.
Which other crops do you grow?
Maize and ground nuts
Have there been any changes in the acreage of cotton relative the acreage of these other crops?
I used to grow more cotton in the past. Things were alright then. Nowadays, cotton doesn't pay very well. Not only that, the types of the cotton are also different. We get as low as only 3 bolls per plant, but in the past we could get as high as 60 bolls per plant. We are now growing on a small scale because we get input loans; if we get a lot of inputs we will not be able to pay our loans.
Which pesticides have you used over the years?
In the past we used 75 and DDT. Then we were later told that there is no longer 75, there is now Thiodan, then we were again told that there is now cabaryl-85. Then Thiodan is no longer available, *(inaudible Thiamex, ndiyo yakuwanika)*, and 85 is still available. We have also used several other pesticides including Agrithane, and several others that i forget, and then there are the Fernkill and Fenvalerate.
Would you say you are now using more or less pesticides now, compared to the past?
These days there seem to be more pesticides, but these have no power to kill pests. We may spend the whole week spraying, but the pests will always remain. If i spray today and go back to my field tomorrow i will still find the worms present.
These days there are more pesticides, but they are weak, yet in the past we had fewer pesticides, but they were more powerful.
Are the pesticides you are using now enough to kill pests?
We are receiving sufficient pesticides in terms of quantity, but the pesticides are insufficient when it comes to their effectiveness to kill pests. They just don't have power at all.
Which herbicides do you use?
In the past we never used any herbicides. We used to weed manually. The use of herbicides is a recent phenomenon. We are still learning to use these herbicides about how they work.
Do you know their names?
Yes, there is roundup, then paraquat and then more recently atrazine. I only started using herbicides a few years ago.
Have you ever been trained in the management of pests?
No, i have never had any education. The only way of killing these pests is by spraying them with pesticides.
Thank You for your participation
TM 06
Which cotton pests are found on your farm?
I come across red bollworms and aphids that suck. There are also American bollworm. There are also some pests that cut the 'head' of the plant as it will be growing. There are also small worms that eat the leaves.
Which of these would you say have grown in population?
I would say the Redboll worms. These are always present from the time cotton starts to have bolls till the time it becomes white. These are the most problematic.
Have pests changed over the past 30 years?
No, they are basically the same pests that were there in the past.
Are there any noticeable changes in terms of pest populations?

There aren't any significant changes as long as pesticides are available to kill them. But, if there are fewer pesticides the pests can increase.
Which types of weeds do you find in your fields?
The most problematic is chinzungu. The other types are not problematic, we just weed them manually without any problems that they give us.
Which pesticides have you used over the years?
I normally use carbryl-85, then mosbran against aphids, and Fernkill. In the 80s we used malvek (inaudible) and karate.
Has the amount pesticides used changed?
We are now using fewer pesticides. These days they are giving us just one bottle per hectare, for instance.
Are the pesticides enough?
No, we are not having enough.
What do you use for weeds?
I never use herbicides, i always weed manually.
Do you use other methods of pest control, besides pesticide use?
No. I only use pesticides.
Have you ever received any inputs on credit?
Yes, even now i still receive inputs on credit. But sometimes they do not give enough for a hectare. To be able to produce enough from the hectare is always impossible because the inputs will be insufficient.

Thank You for your time

TM 07
Which pests are found on your farm?
I normally come across worms, aphids and other pests that do not necessarily stay in the field, they just come and eat and go.
Which pest has been most problematic for you?
I would say aphids are the most problematic. These are the ones that mostly damage our plants. Worms are not normally that problematic.
Are there any noticed changes in the population of these pests?
I think they are getting fewer.
Why do you think they are getting fewer?
I think it's because of the pesticides that we are receiving.
What about them?
They are stronger, they are trying.
And in terms of the pests? Are there any changes?
Well, it depends on the field. For one farmers there may only be found aphids, while in another farmer there may be found worms.
What is found in your own field?
In my field the main pests are worms and aphids.
So, are these the same pests that you were seeing in the past?
No, in the past they were much fewer, and i would always spray them and they would all go.
Which weeds are found in your field?
In my field there is *kapure-pure*. It's white in colour. This is the main weed that i find in my field.
What is its effect?
Kanovhunjira donje
What do you do to it?
I always weed manually.
Do you not use any herbicides against weeds?

No, i do not use any herbicides, i always weed manually.
Which pesticides have you used over the years that you have been growing cotton?
There is rogor, carbary-85, then the one meant for red spider, and then, ummmm. These are the ones we have been using.
Do you recall the names of the other ones?
No, i do not recall.
Which ones are you now using?
We are using 85, rogor, then the one that kills the red spider. These are the ones that we are using these days.
Are there any changes in the amount of pesticides used now compared to those used in the past?
Yes, in the past we used more pesticides. The companies gave us our pesticides all at once, and there was no need to go and look for extra pesticides. Nowadays we are receiving fewer pesticides.
Are the amount of pesticides you are using these days sufficient to control pests on your farm?
Well, it depends on the acreage and the company giving the credit.
How do you control weeds?
I always weed manually; i have never used any herbicides. If i have a little cash i look for people to help and then i pay them.
Who is involved in the handling of pesticides at your home?
I mix, spray, wash my knap sack and even my own clothes that i wear while spraying. I do everything myself. I carry my soap and my towel. I wash myself and my clothes, then i wear my clean clothes and return home.
Do you use any other methods of pest control?
No. I only use pesticides. I receive them from the companies.
Which companies are these?
I have taken loans under contract farming from Cottco, then Grafax, which i am using these recent years.
Have you ever received any training on other methods of pesticide management?
No, i only know to use pesticides.
Thank You for your participation

TM 08
Which pests do you come across on your farm?
The first is aphids. These interfere with the health of our plants. Then there is a worm, called red bollworm that bores into, and stays in the bolls of our cotton. This damages our cotton.
Which of these would you say has increased since you started growing cotton?
I see that the aphids have increased. The red bollworm has also increased. It's because there are some, those of us who do not cut and burn their cotton stalks. The bollworm goes down that stalks and lays its eggs in the roots of the plant. When it rains these eggs will hatch, and it will become impossible for us to control it once it hatches.
Would you say there are any changes in terms of population?
Right now it's not easy to tell whether it is more or less because we have been scaling down on cotton production because of low prices.
Have there been any changes in the types of pests?
The types are still the same as from many years ago
Which weed is found in your fields?
There is the type called chinzungu, this one is really affecting our crops. There is also chaguduma. This one has spikes. This type is also affecting the health of our crops. These are the twoo types that are affecting the health of our crops.
Which pesticides have you used thus far?
i only know one type from the past, the rest i cannot recall. I know Cotguard.
Would you say there have been any changes in the amount of pesticides used over the years?

There is a change, in the past we used Rogor, they say it's now called what, by the way, i don't know, then there is 85, and also Thiamex. It's not there anymore. There is now gukurahundi. Thiamex was used to kill the American bollworm. But i don't know why it was stopped. We do not see it any more. Are the pesticides you are using nowadays enough to control pests on your farm? No, they are not sufficient because i limit my spraying. I try to skip a week between my spray intervals. But, when i skip a week maybe it rains, and then when it rains my pesticide is washed off, and then the worms will eat my crop.

Do you use any herbicides for the control of weeds?

I have not used.

Do you use any other ways of controlling pests?

No, i use only pesticides.

Have you ever received any education of pesticide risks and other forms of non-chemical pesticides?

I have received education concerning scouting for pests in the field.

Thank You for your participation

TM 09

Which pests are found on your farm?

I know heliothis, then there is the red bollworm, and then there are also some other butterflies (moths)

Which of these pests are problematic?

It's the red bollworm

Since you started, have there been any changes in pest population?

The pests are actually increasing in population size.

Are there any changes in types of pests?

The types have not changed. I still see the same pest types that were there since i started farming cotton. There are no new ones, and there aren't any that have disappeared.

Are there any other concerns concerning pests on your farm?

Yes, it appears these pests are now resistant to the type of pesticides that we use to control them.

What do you think might be the cause of this resistance?

I used to think that the type of pesticides that we are receiving to control them do not have power any longer.

So, what are you doing about it?

I am just persevering on as cotton is a crop that i have experience in growing.

Which pesticides have you used over the years in cotton farming?

I have used Dimethoate, Carbaryl 85, Fernkill and Fenvalerate. I am having problems recalling some of the pesticides.

Would you say the amount of pesticides that you use in your cotton farming changed over the years?

No, there is no change. I only see as if it's the names that are changing.

How come you see as if there is no change?

I see that it appears as if Fernkill, for example is simply changing the name, of its changing the percentage of how it kills.

Are the pesticides you use enough to kill the pests that you encounter on your farm?

Since i usually get my inputs on credit under contract farming arrangements, sometimes the pesticides are not enough because the company giving the credit may give not enough pesticides and it may also give after long intervals

Do you use any herbicides?

I do not use herbicides, i always weed manually.

What other methods of pest control do you use?

I often handpick the worms, especially when there are clear signs of their presence. I hand pick and kill them manually. As far as weeds are concerned, i always weed manually.

Have you ever had training on alternative ways of pest control?

No, i have never.

Thank You for your time

TM 10

Which pests found on your cotton fields?

There are worms and aphids that affect the leaves. The worms affect the bolls.

Which pest is giving you the most problems?

It's the red bollworm

Do you notice any changes in pest populations?

Since i am no longer involved in cotton farming as my husband was the one who was active, i would not really know. Never the less, red bollworm is the one that seems to be increasing in population.

Have there any changes in pest types?

No, it's basically the same old types.

Which weeds did you come across when you were actively growing cotton?

We had problems with *bwerere, and kablakwasha*, now there is chinzungu and chaguduma>

Which other crops do you grow?

I grow maize and mhunga. But, its mainly maize.

I no longer grow cotton because i do not have a pesticide sprayer since my husband died. He is the one who used to do the spraying.

Which pesticides did you use during your time?

We would always start with Rogor, against aphids, and then later on as the worms emerged we would switch to gukurahundi until the cotton became white.

Do people now use more pesticides?

The people now say they no longer use rogor and they now use Acetamark.

Did you use any herbicides?

No, we always did our weeding manually.

Did you use any other methods of pesticides?

No, we only used pesticides.

Did these pesticides affect the health of anyone in the household?

No, they didn't.

Thank You for your participation

TM 11

Which are the cotton pests that are found on farm?

There are ladybird beetles that come later on. But, there are those green worms that feed on the leaves, then there follow those red worms that affect the bolls, and then the beetles that come when the cotton has budded. When these ladybirds pee on the cotton, the colour of the cotton will be affected, it will no longer remain untainted white.

Which of these pests would you say have increased in population?

I see the green worms as having increased in population. These also eat the leaves of our crop.

Which weeds are found in your fields?

The one that gives us problems is chinzungu.

Which other crops do you grow?

I also grow maize and ground nuts.

Have there been any changes in the acreage of cotton relative to the other crops?

I am now growing more groundnuts, and i have cut down the cotton acreage.

Why?

At my age, i cannot continue growing cotton because it i s labour intensive. I now find it very difficult for me to carry the knapsack sprayer.

Do you have a knapsack sprayer?

No, i don't. I borrow from those who have.

Which pesticides have you used over the years?

In the past we used to use 85 and Rogor. When we found that it was being infested by a lot of worms i would then use *kurura*.
Do you see any changes in the amount of pesticides used now compared to those used in the past?
I see that there is a change. In the past we would receive all types of pesticides, and we would use and not finish it the whole season. I observe that the pesticides that we used in the past had power. The current pesticides are less powerful.
Therefore, the pesticides used now are not enough to control pests on the farm. We end up selling chickens or goats in order to buy extra bottles of pesticides.
Do you use any herbicides for weeds?
I sometimes buy, but i can't recall the name of the herbicide.
Do you use any other methods of pest control?
I always use pesticides.
Have you ever received any education on alternative methods?
No. Never
Have you ever received protective clothing from cotton companies?
No. Since i started growing, i have never received any protective equipment from the cotton companies.
Thank You for your participation
TM 12
Which pests are found on your cotton farm?
There are certain worms that come behind there, and then there are certain flies that just fly around. Then the worms with green and yellow stripes. Then there is another fly that just flies that just cuts the shooting stem of the plant. This fly has a hard coating. And it just cuts the stem. Then, when the cotton has developed bolls, there comes a worm that feeds on the cotton from outside, and then another one that is red in colour that feed feeds from inside the bolls. The one that feeds from outside is whitish. Then there are also some aphids.
Of these pests, are there any changes in their population that you have seen over the years that you have been involved in the farming of cotton?
My observation is that there is an increase in the population of the worms.
And in terms of general population of pests?
Yes, these days there are generally more pests in cotton than what it used to be like in the beginning.
Then, in terms of types of pests? Are there any new types? Or are there any types that seem to have disappeared?
Yes, there are those that appear like flies. I never used to see them in the past, but nowadays i see them.
Which weeds do you come across in your field?
There is a type that we call chinzungu. This weed is very bad. It just spreads easily, and its effect is that the cotton may easily lose its green colour and start showing a yellowish colour.
Which other crops do you also grow together with cotton?
Nowadays i am increasing my groundnuts acreage. In the past i did not use to grow a large acreage of groundnuts, but we used to grow more cotton. But i have realised that these days it's now better to grow more groundnuts.
Would you say that there have been any changes in cotton acreage relative the acreage of cotton?
Yes, i see that in the past we would grow a small portion of cotton and harvest a lot. Nowadays we grow a bigger portion, but the harvest is much smaller.
And in terms of the other crops, are there changes in acreage?
Yes, it's now different because we are now growing groundnuts, maize, mashava, mhunga, and everything, we are planting. Our acreage will not be very big, but we try to make sure that we diversify and grow a bit of everything so that we can harvest something.
Which pesticides have you used on your farm?

I have used rogor, 85, and gukurahundi. These are the pesticides that we used in the past.
Are these the ones you are still using?
Nowadays there is Acetamark which is used against aphids, and fernkill. And ... i can't recall the other names.
Okay, so has there been any changes in the amount of pesticides used over the years?
Yes, we are now using more pesticides than we used in the past.
Okay, and in terms of how these pesticides work, are there any changes?
It's difficult really to know. In the past there were few worms, so maybe they were easier to control. So for us to really see if the pesticides are working or not is difficult.
And, among the pesticides that you are currently using, are they sufficient to control pests on your farm?
They are sufficient if you get a loan contract with a company that supplies enough pesticides. If you get a loan with a company that supplies pesticides that are not enough you end up selling livestock in order to buy extra pesticides.
Do you use any herbicides?
No, i have never used any herbicides.
Do you use other methods of pest control except pesticides?
No. There are no other ways of managing pesticides.
How do you manage weeds?
We kill weeds by physically weeding them out.
Have you ever received any education or information of other ways of managing pests without using pesticides?
No, i have never.
Thank You for your participation
TM 13
Which pests are commonly found on your fields?
There are green ones, then there are also red ones, then there are... There are several of them.
Can you please identify them?
I don't know their names, can't recall their names
Can you describe them by what they do?
There are those that eat the flowers and bolls, then others that feed on the leaves and others that eat the bolls.
Which, among the pests that you have talked about, have increased in their quantity since you started growing cotton?
Those that have significantly increased are those that are green. These have increased and they do not even respond to the chemical pesticides when sprayed on.
Which part of the plant do they attack?
They eat the actual bolls. Even when a boll has developed into a generous sized one, they just eat.
Do they get inside the bolls?
They get inside.
Has the population of pests increased since you started growing cotton till now?
They have significantly increased, and they do not respond to the pesticides. They don't.
And in terms of the types, are the pesticides you see now the same as those seen in the past?
There have been changes. There is now the green one that i have just talked about.
Wasn't it there in the past?
No, it wasn't there.
Which type of herbs do you have on your fields?
There are plenty of weed types. There is *bwerere, nyabvedzi, goso, chinzungu*.
Which, among these, is most problematic for you?
Chinzungu and goso
How do these affect your agriculture?

Chinzungu is very stubborn, when you weed it out in no time it will have regrown. Even goso, does the same as well. Even chaguduma.
Do you use any chemicals to kill these weeds?
No, we don't
So, what do you do?
We just weed them manually. We just do our best till we harvest.
Which other crops do you also grow?
Maize and mashava and groundnuts
Have there been any changes, over the years, in the acreage of cotton relative the acreage of these other crops which you also grow?
We are now prioritising food crops over cotton. Ground nuts are now giving better returns (compared to cotton, as commercial crops). This season we grew cotton and harvested 3 bales, but we did not realise any income. Cotton is not paying any more. We are dying for nothing. Cotton has a lot of labour.
And, coming to pesticides. Which pesticides do you use?
Pesticides! We start with Acetamark, then followed by 85, and then lastly the Gukurahundi.
Alright.
Compared to the years in the past, are there any changes in the amount of pesticides that you are now using?
Yes, there is a change. We are now using fewer pesticides, and these no longer have power. They absolutely have power.
But, the ones that you use on your farm, are they enough to control pests?
No, they are not. We actually have to buy extra pesticides because the amounts that we receive are not sufficient.
Are there any people who give you?
We get loans
From whom?
We have loaned from Cottco
Do you use any herbicides?
No, we do not use any herbicides, we just ween manually.
Alright. Do you use any other methods of pest control?
No
Have you ever received any education about how to do this?
No, i have never.
And weed, do you have any other means besides weeding manually?
No. We heard that there are chemicals, but these are to be bought. We have not yet had the money to buy such herbicides.

Thank you for your participation
TM 14
Which pests are found on your farm?
There are green worms that start affecting the crop. When the crop start developing bolls, there are worms that get inside the bolls, they are grey in colour. Afterwards, there are aphids. These are the deadliest.
How come?
They sap all the juice of the plant from the leaves leaving the plant looking unhealthy.
When it's the boll?
The grey ones that i have talked about; they are the ones that affect the boll once they get inside the boll. The side of the field where they are spotted usually will not have good cotton yields. When time comes for the cotton to open up the bolls that's when you will realise that there may actually be a whole side that will not have any bolls opening because they will have been eaten by the worms.

Which pest would you say has increased since you started?
It's mainly the worms that affect the bolls?
Since you started, are there any changes that you see in terms of the population of the pests?
Yes, there are changes. I see that the aphid population is becoming lower. But, the worms that affect the bolls, i have realised that if you find the pesticides for them, they can be controlled, but if there is not enough pesticides, there can be a serious problem, cotton may not reach good harvests.
Which weeds are mainly found in your fields?
There is this new pest that appeared recently, in the past it wasn't there. This weed we call it chinzungu. This weed gives us serious problems. Its roots grow deeper into the soil such that if you try to just uproot it you will realise that it will be not so easy to just simply uproot it effortlessly. Even when it is found in the maize field, its roots are also very deep.
Which other crops do you grow?
In the past we tried to grow water melons and *ipwa* in the same field with cotton, but we later realised that it was a health risk, because the pesticides can remain residue it the crops to eat.
Do you also grow other crops, not necessarily in the same field as cotton?
Yes, i also grow groundnuts, sunflowers, maize and cow peas, and mhunga.
Has the acreage of cotton changed over the years, relative the acreage of the other crops?
It has changed. In the past we didn't grow many crops. We mainly grew cotton, and we would grow cotton on the best soils during that time when it gave us good financial returns.
And now?
Now, we are now growing more crops depending on the system of the rainfall. We also now have to reserve a portion for mashava, and mhunga and maize which means the cotton acreage is no longer that big.
Which pesticides have you used in cotton over the years?
The names are always changing. When we started we had, uhhmm, what is the name? Before lambda, we used fenvalerate, and 85, we are still using it, and lambda, and i can't recall all.
Are there changes in the quantity of pesticides used?
Yes, there are changes because we now have more types of pesticides, in the past they were fewer.
Are the pesticides which you use enough to control pests?
Yes, they can be sufficient, particularly if you have enough pesticides. Only that, what is happening now is that the companies from which we get inputs on contract are the ones who are messing up because of the way they are giving us pesticides. We are not able to destroy the pests because they are giving pesticides that are not in tandem with the length of the season. The pesticides are finished before the cotton bolls have even opened up. That is when the worms attack our crops. We must mainly protect against the worms that attack the bolls because the bolls are the cotton. So, we end up selling goats or chickens so that we can buy extra pesticides. Otherwise, if companies gave us enough pesticides we would have good harvests.
Do you use any herbicides?
I have never used herbicides, but i have had education about them and i have seen demonstrations and appreciated how they work and what they are good for.
Other than the use of pesticides, do you use any other methods to control pests?
No.
Have you ever received education on alternative pest management?
No, i have only received education on the usage of pesticides.
Thank you very much for your participation

TM 15
Which pests are found in your fields?
There are some worms that are found. There is a red one, the red bollworm, then there is a green one that feeds on the sap of the leaves. Then there is another one that is found in the soil underneath the plant and it feeds on the base stem of the plant.
Which, among these gives you the most problems?

The red one which feeds on the bolls.
Have there been any changes in pest population since you started growing cotton?
There has been an increase in the population of the red ones
Have there been any changes in the population of all other pests in general?
From the way i see it, i think they have not increased in population because there are now a lot of people who are now growing cotton.
Have there been any changes in the types of pests?
Yes, now there are fewer types.
Which weeds do you come across in your cotton fields?
We mainly have issues with *bwerere and chinzungu*.
And how do you deal with the weeds? Do you use any herbicides?
No, we do not use any herbicides. We only weed manually. So, even when we plant we make sure that we plant the acreage that we will be able to weed.
Which other crops do you grow?
We also grow groundnuts and maize and mhunga.
Have there been any changes in cotton acreage relative the other crops?
There is a slight change because the cotton acreage is slightly larger. The problem that we face is in our inability to weed all the crops in time and have good harvests. If we put most of our efforts in cotton weeding, we also have better cotton harvests. Maize is good in that we will have food at home.
So, is your cotton acreage the same as the past?
No, it's no longer the same acreage, there is a change.
How is that so?
Because some of my children are no longer staying with me at home, they are grown-ups and they have left. So, with the children that i now have at home my acreage is now smaller, which they can manage to work.
Which pesticides do you use on your farm against pest?
We use lambda, and then carbaryl-85. The other ones i just can't recall them right now by their correct names.
Are these the same ones which you used in the past?
In the past we had another one which was called Rogor, but it was discontinued.
Has the amount of pesticides which you use changed over the years?
Yes, because we use the amount of pesticides that equates to the size of land grown.
And, as far as the types of pesticides are concerned, are there any changes?
There is a difference. The pesticides which you used in the past are different from the ones which we are using now.
Why?
Because they design specific pesticides that kill specific pesticides found in the area.
And, are the pesticides which you use sufficient to kill the pests that you encounter on your farm
No. We usually get our pesticides on credit from the cotton companies; so when i get once the companies may just tell me that the pesticides which i collected are already enough, even though i would still need more pesticides to control the pests on my farm. That is the problem that we face which makes the amount of pesticides used on the fields become insufficient.
And in terms of effectiveness in controlling the pesticides, would you say there is a change or not?
There is a difference, the current pesticides are effective, but not as much as the ones which we used in the past.
Do you use other methods of pest control apart from pesticides?
No, there is no other way that i know
Have you ever received any education on alternative methods of pest control and proper handling of pesticides?
No

Thank you for your participation

TM 16

Which pests do you commonly encounter on your farm?

The first one are the aphids which are found if you flip under the leaves of the cotton plant. These aphids suck the sap out of the leaves resulting in stunted growth of the plants. Some aphids feed from the shooting stem of the plant such that the plant will not be able to grow in height. Secondly, there is another pest called the red spider. The effect of this one is that once it is found in the field, the cotton plants will not be any productive no matter how much the plant grows. Then when the cotton has developed bolls, there are types of worms that feed on the bolls. There is the red bollworm which feeds from inside the cotton bolls. Once it enters inside the bolls it becomes uncontrollable. You can't control it. This one should best be controlled while it's still in its egg stage of development. Then there is another one called the American bollworm. In our language we often call it *madyirapazhe* (the one who feeds from outside) because it feeds from outside the bolls. When it feeds, if you fail to control it, it can easily destroy all the bolls in a matter of days. Then there is another worm called heliothis. This is the worm that feeds on the leaves such that when there are a lot of these in the field feeding on the plants, the cotton plants will be left with only the stems without any leaves. There is yet another type whose name i do not really know. We only started noticing it very recently. It's a type of *(inda)* aphid which is white in colour. When you see it you may think that it's just ash, but if you touch it you see it moving. This one once it attacks the cotton plant the whole plant becomes dry. We were informed by the agricultural extension officers that since this is a new pest they do not yet have any specific pesticide that they could recommend to use against it. So, what we normally do is that once we encounter it, we pluck out the affected plant and destroy it.

Of these pests, which ones would you say have increased in population over the past 30 or so years?

The red bollworms, the American and the red spiders are increasing in population every year.

And in terms of population increases in these pests, not just the types, but the population numbers; have there been any changes over the same period?

Yes, i would say over the past twenty years the pest populations have increased, and if we use the type of pesticides that we are receiving to control them they are not getting killed by these pesticides.

Over the same 30 year period, are there any other concerns regarding pests on your farm?

Well, only that the new pest which i have talked about is proving to be really deadly. If it's found in the field for two or three seasons it would be better to stop growing cotton all together. It would be better to put another crop in the same field, such as maize, because if we continue putting cotton they will be increasing in their numbers each year.

Which weeds are found in your field?

There are several types. There is bwerere, then what we call *tsangadzi*, and the other one called goso. On my farm, these are the main weed types that give me most problems.

Which other crops do you grow, apart from cotton?

I also grow maize, mhunga, mashava and ground nuts.

Are there any changes that have happened over the years in the proportion of land dedicated to cotton relative the other crops?

Well, that varies from season to season. There are some season when i place more value on maize and dedicate more land to it. I prefer to dedicate more land to maize and mhunga because these are food crops that give guarantee as far as food security is concerned when i get good harvests.

Which pesticides do you use on your farm?

I use Acetamark to control aphids, then i also use carbaryl 85. Then i also use gukurahundi because the cotton will have started developing flowers and bolls. In controlling worms, i use fernkill, fenvalerate and lambda. Lambdar is the pesticide which we are receiving these days, and my

personal assessment as far as its effectiveness is concerned it that it is failing to control worms. But, if you make efforts to look for another type you see changes.

Are there any changes in the amount of pesticides used over the past 30 years?

It's changing. In the past we used strong powerful pesticides such as Thiodan. The pesticides which we are now using are less powerful, and they are failing to effectively control pest. In order to control the pests, it is best to control the eggs. If we fail to control the worms while they are still eggs, we may control to a certain extent, but not 100 percent.

Are the pesticides which you are currently using enough to control pests?

No, they are not enough because at times we lack the financial means to buy sufficient amounts of pesticides. In fact, that is the biggest problem.

Alright, do you use any herbicides?

In the past we did not use any herbicides. Here we only started using herbicides about 5 or so years ago. Personally i have used an herbicide called Cotguard. I noticed the results that it is a very effective herbicide.

So, have you received education on the other methods of controlling weeds?

Yes. We were educated on these herbicides by the agricultural extension officers.

Do you use any other methods of pest control apart from the use of pesticides?

Yes, sometimes we get into the field as a family and scout line by line, manually killing all the worms that we come across.

Did you receive any education about this method of pest control?

No, it's just an idea that came into my mind and then i just decided to implement it.

Thank You for your participation

TM 17

Which types of weeds do you come across on your farm?

There is the type which we call Nyabvedzi. This type is such that if it receives rain water for just two consecutive days, it will be very difficult to manage it. Then there is Goso. This is another type which we find in our cotton fields. And another type called Chaguduma.

Which cotton pests are commonly found on your farm?

In the beginning, when our cotton is two weeks, some green grasshoppers that feed on the leaves start to appear, and we spray these with Acetamark, or Rogor. When the cotton leaves are showing signs of wilting it means that there will be some aphids, so we use Acetamark. After a week, you get into the field in the second week to see if there would have arrived another wave of pests. If there are, you spray again, but if there aren't, there is no need to spray weekly, you can skip a week and spray the second week. When the cotton starts to develop bolls, there start to appear some worms called red boll worm, which are green in colour, if you see them in the field around December or January, we control them by spraying a pesticide called 85 and Acetamark. We continue using this until February when we may start using Fernkill, as from the 4th of February. Then, the worms would have grown bigger and it will be evident that 85 would no longer be capable of fully controlling them that is why we then introduce Fernkill.

Of these pests, which, as afar as your observation is concerned, have increased in population over the past 30 years?

I have observed that there are more Red boll worms because people are not slashing and burning their cotton stalks as they should. That's the problem. If a worm is left alive this season, it will be impossible to control it next season because it would have developed resistance. It would not die when sprayed on. What i would want to encourage all farmers is to destroy their cotton stalks just the way tobacco farmers destroy theirs and burn them. Just the way our agricultural extension officers encourage.

Have there been any changes in the amount of pests?

Yes, there are some seasons when there are some grasshoppers which invade the fields. When these invade the field, there is very little to harvest from that field. All the leaves will be eaten. In 1987,

there were certain insects called *mamunye*. These insects aren't selective of crops. They can affect any crop apart from cotton. But, these are not resident pests.
What is your observation concerning the amount of pest? Have they increased, remained the same or declined in their numbers?
There are now more pests as compared to in the past. They are actually keeping on growing in their numbers.
And, in terms of types? Have the types of pests remained the same or they have changed?
The types are also increasing because people no longer cut their cotton stalks these days.
Because of that, there are more pests.
Do you have other concerns regarding pests on your farm?
Yes. Particularly with regards to the pesticides. Others have resorted to using the pesticide called Termic as a way of trying to kill the worms. This is so because Lambdar is no longer effective in controlling these pests. It's because we are using the same Lambdar on the same worms over many year that is why it is now resistant.
Which other crops do you also grow?
I also grow ground nuts, maize, mashava.
Have there been any changes in the acreage of cotton relative the acreage of cotton?
Yes, i have cut down on my cotton acreage and increased the maize acreage.
Why did you do that?
Because i realised that cotton is no longer fetching a favourable price on the market. In addition, it is very difficult to manage a large acreage of cotton because it is both a pesticide and labour intensive crop.
Which pesticides have you used over the past 30 years?
We have used Rogor, there wasn't the Acetamark which we are now using. Then Karate, and gukurahundi. These are the pesticides which we used in the past during the 1980s. These were given to us in small metal gallons.
Which ones are you using now?
Nowadays we are using Carbaryl, Lambdar and Acetamark.
How do you compare the pesticides which you were using in the past to the ones which you are using now?
In the past the pesticides had so much strength. Those were real pesticides. If you sprayed with Karate, nothing would be left alive in the field. Karate was deadly.
Are the pesticides which you are currently using enough to control pests on your farm?
Well, the type we are getting such as Lambdar are delivered to us already expired.
Do you use any herbicides?
No, i do not use any herbicides. It's because of my poverty that i cannot afford to buy any herbicides. They are there in the shops but i cannot afford, so i am spent using the hoe for weeding.
Do you use any other methods of pest control?
No, there are no other methods apart from these chemical pesticides.
Have you ever received any training on pest management and the safe handling of pesticides?
Yes, the agricultural extension officers always teach us about that.
Thank You for your participation

TM 19

Which pests are found on your farm?
I normally come across several pests which feed on the crops, but i cannot recall their names.
Which pests have changed?
I now notice that nowadays even *mazingizi* are now coming to attack our crops.
Have there been any changes in pest populations since you started growing cotton?
Yes, nowadays there are more pests
Which weeds do you come across on your farm?

There is *chaguduma, bwerere* and *mhandi*.
Which other crops do you grow?
I also grow maize, ground nuts, mhunga mashava, round nuts, and cow peas.
Have there been changes in the acreage of cotton relative the acreage of the other crops?
In the past my cotton acreage was smaller. Now i grow a larger acreage. I used to also work in the past, so i had a second source of income. Now that i don't have another job, my cotton acreage has increased than it was in the past.
Which pesticides have you used on your farm?
I have used Carbaryl 85, Lambdar. Lambdar is much deadlier on the skin than the type we had in the past.
Are there changes in the pesticides compared in the past?
There are now more pesticides. But they are not effective in controlling the pests. It's mainly because we are receiving our pesticides late after pests have already attacked our crops.
Do you use any herbicides?
No, i always weed manually.
Do you use any other methods of controlling pests other than the use of pesticides?
No, i only use pesticides.
In what ways do the pesticides which you use affect your health?
I have no idea.

Thank You for your participation.

TM 20
Which pests are found in cotton on your farm?
I may not be able to tell you the exact names, but let me explain or describe them. There are worms which are green and red. They eat the boll of the cotton. Then there are aphids which damage the leaves.
Since the time you started growing cotton, are there any changes in the population of these pests?
These green and red worms are the ones which are increasing in their population.
Which weeds are found in your cotton field?
There is a red weed that affects the crop.
Which other crops do you grow?
I also grow maize, round nuts and ground nuts.
Over the years have there been any changes in the relative acreage of cotton compared with the other crops?
I normally make my cotton size bigger because it is from cotton that I get my money. The maize acreage is slightly smaller. My groundnuts is also larger because that is my other cash crop from which i get money for school fess.
Which pesticides do you use to control pests?
I use Carbaryl 85. This pesticide makes the cotton grow healthy and it makes the plant bear a lot of cotton bolls and to make the bolls stay on the plant without falling off to the ground. Then i also use Acetamark which kills aphids and then Fernkill which kills the worms, those green and red ones which i talked about.
Are these the same pesticides which you have been using in the past?
In the past we used Fernkill, but Acetamark and Fernkill are fairly recent ones which we didn't use in the past. In the past there was Karate which we used against the worms, and Rogor was used against aphids.
Are there any changes in terms of pesticides used in the past compared to those used now?
Yes, there are differences. We now use more pesticides than we used in the past. In the past we used only two types of pesticides which were very strong. Nowadays there are more pesticides which are no powerful at all.

From your observations, are these pesticides which you use now enough to control pests on your farm?
Yes, they are killing, but they are not as effective in killing worms. The pesticides have not enough power to kill the worms.
Do you use any herbicides?
No. I have never used any herbicides, i always weed manually.
Do you use any other methods of pest control?
No, i only use pesticides.
Have you ever received any training on alternative ways of controlling pests?
No. i only know that these pests can only be controlled by using pesticides on them.

Thank You for your time.

TM 21
Which pests do you come across on your farm?
The red bollworms, the red spider and *kafuwe.*
What does this kafuwe do?
It makes the plants get dry
Since you started growing cotton, have there been any changes in pests?
Yes, since we started i am noting that the pests are increasing. This is because the pesticides which they are giving to us do not have power.
Are there any changes as far as the particular types of pests themselves are concerned?
No, there are no changes. There are the same pests that have always been there.
Which weeds do you find in your fields?
There is *tsangadzi*, then *san'ombe*, and what we call *chidyahumba*
Are there any other concerns regarding pests?
Yes, these pests feed on the crops a lot. If you spray, say today, and go back to inspect tomorrow, you will still find them alive. They don't die.
Which other crops do you grow?
Maize, ground nuts, cow peas and round nuts
Are there any changes in the size of land dedicated to cotton relative the acreage of the other crops?
Yes. The acreage of cotton has declined while that of maize and groundnuts has increased.
Which pesticides have you used on your farm?
I have used Karate, Rogor, Fernkill and Lambda and Acetamark and Carbaryl 85.
Have there been any changes in the amount of pesticides used over the years?
They have been limiting the amount they have been giving us.
Nowadays we are now receiving fewer pesticides and they are not enough. By the time we harvest all our pesticides would have been finished.
How do you control weeds?
I normally just weed. I have used herbicides, but because of cost limitations, i normally just weed manually.
Are there any other ways of controlling pests apart from using pesticides?
Yes, in the past when pesticides were problematic we would use hot chillies and *mukonde*. We would mix these and use them as pesticides. They can be effective. I have done this as a way of testing, and i noticed that the worms can be severely affected, even die.
Thank You for your time
TM 22
Which cotton pests are found on your farm?
I do not know the actual names of some of them, but i know that they affect the plant when it starts to develop flowers. If you go for two weeks without spraying with chemicals you may lose your

plant. There are then those that eat on the bolls. Then there are some that eat the leaves. The one that damages the bolls is called the American bollworm.

Of these pests, which one has increased in population over the past 30 years?

The one that i am calling the American boll worm which damages a lot of the bolls.

In the past 30 years, have there been any changes in the types and populations of these pests?

There have been a lot of the red ones the ladybird beetles in the recent years. They appear at the end, when the cotton bolls start opening up. They discolour the cotton and make it become brown in colour. But we spray them with Carbaryl 85 and manage to control them. These ones die quickly.

Have there been changes in types of pests?

There hasn't really been a change in types, just the population of the existing ones. But these are easily controlled if you work hard, like what i used to do. I would just spray pesticides on them and they would die. Nothing would enter the field. I was encouraged top control these because my cotton would be very clean at the end and it would give me a lot of money because of its grade.

Which weeds are found on your farm?

There are several types. There is chaguduma, then there is another one called chinzungu which really damages the crop. Then another one that does not die which continues feeding on your plant's nutrients till you harvest. We call this one *karoi*.

Which other crops do you grow?

I also grow maize and groundnuts. I do not grow a lot over the past two years because my wife died, and this has affected my farming activities and production.

In the past 30 years, has the acreage of cotton changed relative the acreage of the other crops?

Yes. I now grow cotton on a very small scale, and i have given part of my land to my sons.

Which pesticides do you use on cotton?

In the beginning we used Carbaryl, then after it we would use Thiodan. Thiodan was very effective in controlling pest, but they stopped giving us because women were dying. When they were cutting the cotton stalks and working on the cotton fields, they would inhale the dust and this dust affected their health such that many would go to the hospitals. That is when they realised that the pesticide was dangerous as it had damaging effects on health. Those who are not very well experienced in cotton farming do not use carbaryl, they use the liquid one which we call gukurahundi. If you are using gukurahundi, without Carbaryl, your cotton will not give you a give harvest. Carbaryl builds the plant together with Acetamark, which we call Rogor. These are the things which work on cotton if you are to have good plants.

Have there been any changes in the amount of pesticides used over the years?

No. I don’t see any changes. What makes changes in cotton, particularly with regards to the yields is because people are planting their cotton late. They plant ever other crop first and then plant cotton at the end.

Are the amount of pesticides which you are using per hectare enough to control the pests on your farm?

No, they are not enough, particularly if you get your inputs on credit. You must have some inputs which you must buy on your own as a farmer. If you wait till the company gives you the pesticides you will work for nothing. That is where we have serious problems with the companies such as Cottco when we ask them to give us loans, but when they do so what they give us is not enough and sufficient. It is wise to always buy something on your own

Do you use other methods of pest control?

No, i only use pesticides.

Do you use herbicides?

Yes. I use herbicides because there is no way that one can spend all their time weeding in the field. I have often used herbicides on maize and then used the hoe on the cotton field.

Which herbicides do you use?

I can’t recall the name, but we mix a bluish coloured herbicide and a white one and spray.

Have you ever received any education about the control of pests?

I have always been close to the agricultural extension officers who have always been giving me advice.
Thank You for Your Participation

TM 23

Which pests are found on your farm?

In the beginning we come across jassids and aphids, then there follows some green worms which eat the leaves, then there are those red in colour which get inside the bolls.

Which of these pests have been most problematic?

The red ones are the most problematic. The green ones can be controlled with the chemicals when sprayed on, unlike the red ones which are difficult.

Have the population sizes of any of these pesticides increased?

The amount of pesticides is increasing because there are some farmers who are not taking care of their cotton stalks. The worms that result as a result of the ratoon crops are very deadly. They easily destroy the bolls.

Have there been any changes in the types of pests in the past 30 years?

I could say that there are those pests such as aphids which appear at the beginning of the cotton season and at the end. At the end they are usually worse.

Are the types of pests you came across around 30 years ago the same as the ones you come across now?

No. there is a difference. In the past there were fewer red bollworms. These days there are now more of such because of the increasing practice of people not cutting the cotton stalks.

Which weeds are you coming across?

In the past we had issues with some weeds that were quite soft and easy to control. Nowadays we have problems with weeds such as chidhongi or chidhongi, and another type that is so hard that affects the health of the plants.

Which other crops do you grow?

I also grow maize, mhunga and mashava because our season is changing.

Has the acreage of cotton changed relative the acreage of the other crops?

Yes, there have been changes. In the past cotton was doing very well, but nowadays it is not growing well, and there are now a lot of pests, that is why i am just increasing the acreage of ground nuts and maize.

Which pesticides do you use on your farm?

In the beginning we were getting good pesticides. They would give us Rogor, carbaryl, Thiodan and gukurahundi. They would give us about four or 5 different types. These days Rogor is no longer there. They are now giving us this powdered one.

Over the past 30 years, has the amount of pesticides you use on your farm changed?

In the past we used to receive enough, but these day there is just more but they are not working.

Are the pesticides which you are receiving enough to control pests on your farm?

No. The problem is that we are not getting enough pesticides. They are giving us the pesticides too late when the pests have already attacked our crops. In addition, sometimes they don't even give us at all.

Do you use any herbicides?

No. I have always used manual weeding

Do you use any other methods of pest control?

No. But what i often do is that i burn the grass in the area that surrounds my field so that it remains clean and this kills the pests in this area. When i plant my cotton there will not be any creeping insects getting into my field.

Thank You for your time.

TM 24

Which pests are found on your farm?

The pest that first affects cotton are the aphids. These are followed by the American bollworm. After this then there comes the Red bollworm. After the red bollworm the cotton is then affected by the ladybird beetles. This beetle appears when the cotton buds open, and it affects the quality and colour of the cotton.
Are there any changes in the pests' population over the years?
I can say these pests have not changed. They are the ones which affect pests, including some grasshoppers (jassids)
In terms of the types of pests, are there any changes that have been noticed over the years?
Well, in the past there were fewer pests, but there were many people who were growing cotton. Nowadays there are fewer farmers still growing cotton, and those growing are now growing on a smaller scale, but the pests are always appearing in larger quantities as soon as cotton is planted.
Why are there fewer cotton farmers now?
It's all about monetary reasons.
Which weeds are found on your farm?
Nowadays it's better because there are now herbicides. In the past we had serious problems with the weed call chinzungu. That was a bad one. Then there was also serious problems with chaguduma, also known as chidhongi. These were very difficult to control without any herbicides in the past.
You said earlier on that you also grow other crops such as maize, groundnuts and mashava. So tell me, have there been any changes over the years in the acreage of cotton relative the acreage of these other crops?
Nowadays it's different. I am now old, i no longer grow a large scale. I used to grow cotton on a larger scale.
Which pests did you use then?
We used to use Rogor, Carbaryl. Thiodan and Gukurahundi. These are the four pesticides which we used to use.
Are they still used?
These days Thiodan is no longer there. Carbaryl is still there. Gukurahundi is there but the pesticides we call gukurahundi are now being called by other names. But Thiodan was very strong.
Do you still use the amount of pesticides as you used in the past?
Nowadays there are more pesticide varieties, but the way they work or their effectiveness is different.
Would you say these are enough?
The pesticides may be enough, but their problem is they ineffectiveness when it comes to killing the pests. Some pests die, others do not.
Why do some pests not die?
Because the pesticides will be without any power; they will be weak.
Which other ways of controlling pests other than using pesticides may be used?
If you keep your field clean, the area that surrounds the field, if you weed it and spray to control the pests before them get into the cotton field, it takes longer for them to affect the cotton.
Which other ways do you control weeds?
Well, there is only manually weeding using a hole and using an ox-drawn cultivator.
Which herbicides have you used?
I have used Stella-star, and i am using this in maize.
Do you have any other concerns regarding cotton?
Yes. There are some farmers who are now banking on ratoons, these ratoon crops. When we started growing cotton, no farmer was permitted to leave their cotton stalks in the fields. It was a statutory offense. There were inspections. But nowadays it's all different.

Thank you for participation

TM 25

Which pests are found on your cotton farm?
There are aphids, then worms that feed on the bolls, then there are some worms which are hairy which feed on the leaves before the flower buds have developed
Which of these pests would you say have increased over the past 30 years?
Aphids and red bollworms have increased in population.
Have the types of pests changed over the past years?
Yes, they are increasing.
What do you think is causing this change in the types of pests?
i think it is because of the changes that are happening in the climate which are causing there to be more types of pests.
Which types of weeds do you come across on your farm?
There is chidhongi and chinzungu
Which other crops do you grow?
I also grow ground nuts and maize
Which pesticides do you use on your farm?
In the past we used Rogor, which was in liquid for; now we use a powdered one called Acetamark on aphids. Then, against worms those which feed from outside we use Carbaryl, and them against those worms which enter the bolls they often change the type of pesticides they bring.
Has the amount of pesticides used changed over time?
Yes. Nowadays there are fewer pesticides as compared to the past.
Are the pesticides you are using now enough to control pests on your farm?
No they are not enough. For those of us who are on contact farming who receive inputs on credit, we always get pesticides that do not match the acreage. Sometimes we end up having to buy on our own or risk losing the crop to worms.
Do you use any other methods of pest control apart from using pesticides?
Yes, sometimes i use a sharp object, such as a wire to pierce the worm while it is inside the boll.
Do you use any herbicides?
No. I always weed manually.

Thank You for your Participation

TM 26
Which cotton pests are found on your farm?
There are those which feed on the cotton leaves. They are green in colour. They call them leaf-eaters. Then there are the other ones which are striped which eat from the time when there are flowers, we call them American Bollworms. Then there is another one which has very sharp teeth, like a cutter, which once it chews on the plant it wilts and dries.
Of these, over the past 30 years, are there any which have increased in population?
Yes, there are those which have increased in their populations over the past couple of years. They appear like grasshoppers, but they do not fly, they feed on the cotton (jassids).
Have the types of the pests changed over the years?
Yes, there are different types of pests. Worms are found in different varieties. There is the worm that feeds on leaves only, then there is the worm that eats the boll, and then there is the one that eats the flowers, which starts to eat inside the flower before the flower develops into the boll.
Are these worms the same as those which were there in the past?
Yes, they are still the same. We use pesticides against them, but we never eradicate them.
Have the pests changed in terms of their population numbers?
Yes. Nowadays there are more pests because there is too much sun, and it is too warm.
Which weeds do you find on your fields?
There is chaguduma, nyabvedzi and *ka brakwasha* which when it blooms it develops a red flower.
Apart from cotton, which other crops do you grow?
I also grow maize and mashava.

Has the acreage of cotton changed over the years, relative the acreage of the other crops?
I decided to cut my acreage because of low returns; in the past when we grew cotton we would have good returns. With the passage of time i decided to downscale my cotton acreage.
Which pesticides do you use on your farm?
In the past we used to use Rogor, Carbaryl 85 and Thiodan. When we stopped using Thiodan, we started using the likes of Fernkill which were introduced recently.
Are the pesticides which you use enough?
No, they are not enough. Under normal circumstances, per hectare i should use two bottles of Rogor, two bottles of Fernkill, and two bottles of Carbaryl. But, nowadays they give us only one bottle, and this is not enough, resulting in our cotton becoming susceptible to pest attacks.
Do you use any other methods of pest control apart from chemical pesticides?
Yes, chillies can work. They should be crushed and soaked in water for one week. After one week if you spray on aphids with the chillies concoction the aphids will die.
Do you use any herbicides?
Yes, but if there are no herbicides i weed manually or i use an ox-drawn cultivator.
Thank You for your Time

TM 27
During the 1980s we didn't have problems with inputs because we were being supplied from Agricura. This company used to supply enough inputs, including chemical pesticides such as Carbaryl, dimethoate, gukurahundi, and all, for us to use. Thereafter, when Agricura left there came another company which gave us all our input requirements in time. Our agriculture extension officers used to teach us on the proper ways of farming. But, we also listened to farming programs over the radio. Nowadays we no longer have radio programs, but in the past such programs used to give us guidance.
Which pests are found on your farm?
There are red bollworms, the American bollworm, then the cotton strainers (*twunana Rudo*), the leaf eaters and another one called *chinungu*, i do not remember what it's called in English, then there is a yellow one that hides behind the leaf, and then there are jassids. When jassids attack, they suck sap from the plant, and you notice your plant wilting. Then there are also aphids, some white flies, which are also found in cotton. Then there is also another one which has a hard shell. Then there are also some cut worms which attack the plant from the bottom of the plant.
When it comes to spraying, we were taught by our extension officers to always scout before spraying so that we would know what kind of pest is present, so that we would also know the type of pesticides to use.
Have pests changed in population?
Yes, there are more pests now than there used to be in the past.
Are there any changes in the types of pests?
Yes, there seem to be some changes.
In the past we would only spray with Rogor after our cotton had grown in height by several centimetres. But, nowadays, we need to spray it while it will still be very short near the ground.
Which weeds are found in cotton in this area?
Here there is *gezi*, then *chaguduma*, also known as *chidhongi*, then there is *tsangadzi,* and then another one called chenamoyo, and another one called *chimbiya*, also known as *chinzungu.*
Which other crops do you grow?
I also grow maize, ground nuts and sunflowers
Have there been changes in your cotton acreage, relative the acreage of the other crops?
Yes, in the past my acreage was larger than it is now. It's mainly because nowadays i do not get enough inputs.
Which pesticides do you use?
There is Acetamark, Lambda, Fernkill, and Fenvalerate, and Carbaryl. In the past we had Thiodan, and Rogor called Shasha, and Dimethoate. Nowadays we use Acetamark.

Have there been any changes in the amount of pesticides used?
Nowadays there are fewer pesticides. In the past the pesticides had a lot of power.
Are the pesticides which you are using enough to control pests on your farm?
No, they aren't enough. You may spray but soon after spraying you will still find pests.
Do you use any other methods of controlling pests apart from pesticides?
No. Only pesticides are effective.
Do you use herbicides?
Yes, i have used Roundup, and bradex and other herbicides.
Have you ever received education on alternative methods of pest control?
Yes, we were taught that after harvesting we should destroy all cotton stalks by burning so that all remnants of pests would be destroyed.

Thank You for your participation
